# Utility of Exome Sequencing for Diagnosis in Unexplained Pediatric-Onset Epilepsy

**DOI:** 10.1001/jamanetworkopen.2023.24380

**Published:** 2023-07-20

**Authors:** Hyun Yong Koh, Lacey Smith, Kimberly N. Wiltrout, Archana Podury, Nitish Chourasia, Alissa M. D’Gama, Meredith Park, Devon Knight, Emma L. Sexton, Julia J. Koh, Brandon Oby, Rebecca Pinsky, Diane D. Shao, Courtney E. French, Wanqing Shao, Shira Rockowitz, Piotr Sliz, Bo Zhang, Sonal Mahida, Christelle Moufawad El Achkar, Christopher J. Yuskaitis, Heather E. Olson, Beth Rosen Sheidley, Annapurna H. Poduri

**Affiliations:** 1 Epilepsy Genetics Program, Boston Children’s Hospital, Boston, Massachusetts; 2 Department of Neurology, Boston Children’s Hospital, Boston, Massachusetts; 3 F.M. Kirby Neurobiology Center, Boston Children’s Hospital, Harvard Medical School, Boston, Massachusetts; 4 The Manton Center for Orphan Disease Research, Boston Children’s Hospital, Boston, Massachusetts; 5 Department of Neurology, Harvard Medical School, Boston, Massachusetts; 6 Harvard Medical School, Boston, Massachusetts; 7 Department of Pediatrics and Neurology, University of Tennessee Health Science Center, Memphis; 8 Division of Newborn Medicine, Department of Pediatrics, Boston Children’s Hospital, Boston, Massachusetts; 9 Research Computing, Department of Information Technology, Boston Children’s Hospital, Boston, Massachusetts; 10 Division of Molecular Medicine, Boston Children’s Hospital, Boston, Massachusetts; 11 Biostatistics and Research Design Center, Institutional Centers for Clinical and Translational Research, Boston Children’s Hospital, Boston, Massachusetts; 12 Broad Institute of MIT and Harvard, Cambridge, Massachusetts

## Abstract

**Importance:**

Genomic advances inform our understanding of epilepsy and can be translated to patients as precision diagnoses that influence clinical treatment, prognosis, and counseling.

**Objective:**

To delineate the genetic landscape of pediatric epilepsy and clinical utility of genetic diagnoses for patients with epilepsy.

**Design, Setting, and Participants:**

This cohort study used phenotypic data from medical records and treating clinicians at a pediatric hospital to identify patients with unexplained pediatric-onset epilepsy. Exome sequencing was performed for 522 patients and available biological parents, and sequencing data were analyzed for single nucleotide variants (SNVs) and copy number variants (CNVs). Variant pathogenicity was assessed, patients were provided with their diagnostic results, and clinical utility was evaluated. Patients were enrolled from August 2018 to October 2021, and data were analyzed through December 2022.

**Exposures:**

Phenotypic features associated with diagnostic genetic results.

**Main Outcomes and Measures:**

Main outcomes included diagnostic yield and clinical utility. Diagnostic findings included variants curated as pathogenic, likely pathogenic (PLP), or diagnostic variants of uncertain significance (VUS) with clinical features consistent with the involved gene’s associated phenotype. The proportion of the cohort with diagnostic findings, the genes involved, and their clinical utility, defined as impact on clinical treatment, prognosis, or surveillance, are reported.

**Results:**

A total of 522 children (269 [51.5%] male; mean [SD] age at seizure onset, 1.2 [1.4] years) were enrolled, including 142 children (27%) with developmental epileptic encephalopathy and 263 children (50.4%) with intellectual disability. Of these, 100 participants (19.2%) had identifiable genetic explanations for their seizures: 89 participants had SNVs (87 germline, 2 somatic mosaic) involving 69 genes, and 11 participants had CNVs. The likelihood of identifying a genetic diagnosis was highest in patients with intellectual disability (adjusted odds ratio [aOR], 2.44; 95% CI, 1.40-4.26), early onset seizures (aOR, 0.93; 95% CI, 0.88-0.98), and motor impairment (aOR, 2.19; 95% CI 1.34-3.58). Among 43 patients with apparently de novo variants, 2 were subsequently determined to have asymptomatic parents harboring mosaic variants. Of 71 patients who received diagnostic results and were followed clinically, 29 (41%) had documented clinical utility resulting from their genetic diagnoses.

**Conclusions and Relevance:**

These findings suggest that pediatric-onset epilepsy is genetically heterogeneous and that some patients with previously unexplained pediatric-onset epilepsy had genetic diagnoses with direct clinical implications.

## Key Points

**Question:**

What are the diagnostic yield and clinical utility of genetic sequencing for patients with unexplained pediatric epilepsy?

**Findings:**

This cohort study of 522 children with previously unexplained epilepsy used exome sequencing to identify and clinically confirm diagnostic results for 100 children, including 89 with single nucleotide variants and 11 with copy number variants. Individuals with earlier seizure onset, intellectual disability, and motor impairment were more likely to have diagnostic results, and at least 29 patients had changes in treatment, surveillance, or prognosis based on their genetic diagnoses.

**Meaning:**

These findings suggest that for children with unexplained epilepsy, genetic evaluation yielded precise diagnoses with direct clinical implications.

## Teaser

This cohort study examines the genetic landscape of pediatric epilepsy and clinical utility of genetic diagnoses for patients with epilepsy.

## Introduction

Epilepsy, defined by recurrent unprovoked seizures or a single seizure with risk factors for developing others,^1^ is a common disorder often presenting in infancy or childhood^2,3^ and associated with comorbid conditions, including intellectual disability and autism spectrum disorder (ASD). Approximately 1 in 3 individuals with epilepsy have medically refractory seizures.^4^ Accordingly, patients, families, and clinicians seek underlying explanations and potentially etiologically specific treatments. Recent studies have demonstrated that a substantial proportion of nonacquired epilepsy is caused by inherited and de novo variants in several brain-expressed genes,^5,6^ providing insight into developmental and epileptic encephalopathies (DEE), genetic generalized epilepsy (GGE), and nonacquired focal epilepsy (NAFE), sometimes involving the same genes.^5,7,8,9,10^

Even in the research setting, only 30% to 50% of individuals with presumed genetic epilepsy have known genetic explanations.^11^ The discrepancy between presumed vs identified molecular diagnoses highlights a gap in understanding of the genetic causes of epilepsies. Furthermore, millions of individuals with presumed genetic epilepsy do not have identified genetic conditions, in part due to limited access to sequencing and challenges in interpretation of findings in many settings.^12^

Increasing potential for precision diagnosis has fueled a growing focus on precision medicine for the epilepsies.^13,14,15^ A genetic diagnosis provides an end to the diagnostic odyssey for patients and families and may inform prognosis, recurrence risk, and screening for additional clinical features.^12^ These latter aspects, and the knowledge that a search for a cause of the epilepsy has been attempted, reflect the potential for clinical and personal utility, which has not been systematically studied, to our knowledge.^16,17^

Leveraging a prospectively ascertained, single-institution cohort of 522 individuals with a range of pediatric-onset epilepsy phenotypes and performing exome sequencing (ES), we report diagnostic results and their clinical utility.

## Methods

### Study Cohort

This cohort study was approved by the Boston Children’s Hospital (BCH) institutional review board. All participants provided consent and assent when able. We enrolled biological parents and affected siblings whenever possible. Data were analyzed using descriptive statistics and reported according to the Strengthening the Reporting of Observational Studies in Epidemiology (STROBE) reporting guideline.

Between August 2018 and October 2021, we recruited individuals from the BCH Department of Neurology and Division of Epilepsy and Clinical Neurophysiology inpatient and outpatient units. Patients with nonacquired epilepsy with unknown genetic etiology were eligible. We did not exclude patients with nonspecific brain magnetic resonance imaging abnormalities, focal cortical dysplasia, or nodular heterotopia. We included patients with abnormal electroencephalogram findings without clinical seizures (eg, DEE with spike-wave activation in sleep), as their genetic causes are expected to overlap with clinical epilepsy. We excluded patients with events suspicious for seizure without definitive epilepsy. DNA was collected as previously described,^18,19^ with details provided in the eMethods in Supplement 1).

### Phenotypic Assessment

We reviewed clinical data from the BCH electronic medical record (EMR) and referring clinicians. We categorized age of seizure onset as neonatal (<1 month), infantile (1 month to <12 months), early childhood (1 year to <6 years), school-aged (6 years to <14 years), or adolescent (≥14 years). Seizure and epilepsy types were classified according to the International League Against Epilepsy classification by treating physicians and confirmed or reclassified by study epileptologists (K.N.W., N.C., C.M.E.A., H.E.O., and A.H.P.).^20,21,22,23,24^ Each patient was categorized as DEE vs non-DEE, and the non-DEE group divided into GGE (with specific idiopathic generalized epilepsy [IGE] syndromes noted), NAFE, or combined generalized and focal epilepsy. We assessed for the presence of intellectual disability, classified intellectual disability as borderline, mild, moderate, severe, or profound, based on reported IQ in neuropsychological evaluations (when available) or documentation by neurologists of developmental skills and supports needed, classified using standardized published criteria.^25^ We reviewed the description of the motor portion of the neurological examination and descriptions of motor function (eg, motor milestones, activities of daily living). We assessed for evidence of abnormalities in tone (hypotonia, hypertonia), movement disorder, cerebral palsy, and other diagnoses. We noted relevant neurological family history (including febrile seizures). As variants were identified, we reassessed clinical data relevant to the specific gene.

### Variant Identification and Classification

We identified rare, predicted damaging, and clinically relevant variants using standard variant calling and analyses (eMethods in Supplement 1).^19^ Variants were reviewed by a multidisciplinary team of pediatric neurologists, epileptologists (C.M.E.A., C.J.Y., H.E.O., and A.H.P.), genetic counselors (L.S., S.M. and B.R.S.), and additional researchers (H.Y.K. and A.M.D.) with expertise in epilepsy genetics. We classified variants as pathogenic (P), likely pathogenic (LP), or variants of uncertain significance (VUS) according to the American College of Medical Genetics and Genomics/Association for Molecular Pathology guidelines.^26^ Variants were deemed diagnostic if they were P or LP in a gene associated with the patient’s phenotype or VUS in a gene associated with the phenotype but with unavailable parental segregation data.

### Return of Results

All families opted to receive results. Clinical confirmation was conducted using original samples maintained at GeneDx’s Clinical Laboratory Improvement Amendments–certified laboratory. Clinical reports were issued and families notified of results by treating neurologists and/or qualified clinicians from the study team through the BCH Epilepsy Genetics Clinic.

### Assessment of Clinical Utility

For participants receiving diagnostic results at BCH, we evaluated notes for data regarding clinical utility^16^: impact on treatment or clinical management and/or change in prognosis. We noted mention of personal utility (eg, relief, referral to gene-specific advocacy organizations). For participants with results communicated outside BCH, we assessed whether management recommendations would have been warranted based on the genes involved.

### Statistical Analysis

To identify phenotypic factors associated with diagnostic findings, we performed a bivariate analysis for each variable, including sex, age at seizure onset, DEE or intellectual disability, ASD, attention deficit hyperactivity disorder (ADHD), motor impairment (eg, cerebral palsy, hypertonia, hypotonia), and history of afebrile seizure in a parent. We included these variables in a multivariable logistic regression model using R statistical software version 3.2.3 (R Project for Statistical Computing) and SPSS statistical software version 27.0 (IBM) with 2-sided *P* < .05 as the statistical significance threshold. Data were analyzed on a rolling basis through December 2022.

## Results

### Cohort Characterization

We enrolled 522 individuals, including 269 (51.5%) male patients, with a mean (SD) age at epilepsy onset of 1.2 (1.4) years and a mean (SD) age at assessment of 9.6 (6.7) years (Table 1). We classified 142 individuals (27.2%) as DEE. Individuals without DEE included 127 individuals (24.3%) with GGE, 53 individuals (10.2%) with specific IGE syndromes, 152 individuals (29.1%) with NAFE, and 48 individuals (9.2%) with combined generalized and focal epilepsy. The most frequently observed syndrome was infantile epileptic spasms syndrome (IESS), reported in 46 individuals (8.8%). Other diagnoses included childhood absence epilepsy (34 individuals [6.5%]), Lennox-Gastaut syndrome (24 individuals [4.6%]), self-limited epilepsy with centrotemporal spike (16 individuals [3.1%]), juvenile myoclonic epilepsy (14 individuals [2.7%]), and epilepsy with myoclonic–atonic seizures (11 individuals [2.1%]). Individuals with seizure onset in early childhood represented the largest subset (229 individuals [43.9%]). Seizures were reported to be refractory to antiseizure medications at last follow-up in 281 participants (53.5%). Comorbidities were common, including intellectual disability in 263 individuals (50.4%). In addition, 75 individuals (14.4%) had ASD and 71 individuals (13.6%) had ADHD. A total of 99 individuals (18.9%) had had previous nondiagnostic clinical genetic testing (ie, panel or chromosomal microarray analysis).

**Table 1.** Demographic Characteristics and Epilepsy Phenotypes

Characteristic	No. (%) (N = 522)
Sex	
Male	269 (51.5)
Female	253 (48.5)
Age at seizure onset	
Mean (SD), y	1.2 (1.4)
Neonatal (<1 mo)	20 (3.8)
Infantile (1 to <12 mo)	105 (20.1)
Early childhood (1 to <6 y)	229 (43.9)
School-aged (6 to <14 y)	135 (25.9)
Adolescent (≥14 y)	33 (6.3)
Epilepsy type	
DEE	142 (27.2)
Non-DEE	
GGE	127 (24.3)
IGE	53 (10.2)
NAFE	152 (29.1)
Combined generalized and focal	48 (9.2)
Epilepsy syndrome diagnoses	
Syndromes associated with refractory seizures or developmental comorbidities	
Any	118 (22.8)
Infantile epileptic spasms syndrome	46 (8.8)
Lennox-Gastaut syndrome	24 (4.6)
Epilepsy with myoclonic-atonic seizures	11 (2.1)
Epilepsy with eyelid myoclonia	9 (1.7)
Spike-and-wave activation in sleep	7 (1.3)
Landau-Kleffner syndrome	5 (1)
Sleep-related hypermotor epilepsy	4 (0.8)
Myoclonic epilepsy in infancy	4 (0.8)
Dravet syndrome	3 (0.6)
Epilepsy of infancy with migrating focal seizures	2 (0.4)
Febrile infection-related epilepsy syndrome	2 (0.4)
Hemiconvulsion-hemiplegia epilepsy syndrome	1 (0.2)
Syndromes associated with milder prognosis	
Any	75 (14.4)
Childhood absence epilepsy	34 (6.5)
Self-limited epilepsy with centrotemporal spikes	16 (3.1)
Juvenile myoclonic epilepsy	14 (2.7)
Juvenile absence epilepsy	3 (0.6)
Self-limited infantile epilepsy	3 (0.6)
Self-limited epilepsy with autonomic seizures	2 (0.4)
Epilepsy with generalized tonic-clonic seizures alone	1 (0.2)
Self-limited focal epilepsy	1 (0.2)
Photosensitive occipital lobe epilepsy	1 (0.2)
Responsive to ASMs (seizure-free)	222 (46.4)
Intellectual disability	
None	259 (49.6)
Borderline	74 (14.2)
Mild	79 (15.1)
Moderate	59 (11.3)
Severe	38 (7.3)
Profound	13 (2.5)
Other neurodevelopmental diagnoses	
Presence of ASD	75 (14.4)
Presence of ADHD	71 (13.6)

Abbreviations: ADHD, attention deficit hyperactivity disorder; ASD, autism spectrum disorder; ASM, antiseizure medication; DEE, developmental and epileptic encephalopathy; GGE, genetic generalized epilepsy; IGE, idiopathic generalized epilepsy; NAFE, nonacquired focal epilepsy.

### Summary of Genetic Diagnoses

**Figure 1.**
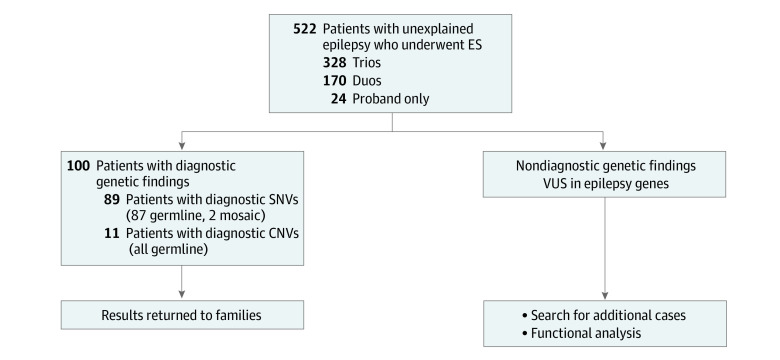
Summary of Diagnostic Yield From Exome Sequencing (ES) Patients With Unexplained Epilepsy A total of 522 patients with previously unexplained epilepsy were enrolled and underwent ES, with 1 or both parents as available. We identified diagnostic single nucleotide variants (SNVs) in 89 individuals. These pathogenic or likely pathogenic variants and diagnostic variants of uncertain significance (VUS) were clinically confirmed and returned to patients and families. Dedicated copy number variant (CNV) analysis of the ES data identified an additional 11 diagnostic CNVs, which were also returned to patients and families. Candidate gene findings and VUS in epilepsy-associated genes that were not determined to be diagnostic were not returned to families but will be reevaluated as additional data emerges or in the eventual emergence of functional data supporting pathogenesis.

Sequencing was conducted on 328 trios (17 individuals with siblings), 170 duos with only 1 biological parent available (8 individuals with siblings), and 24 singletons (4 individuals with siblings) (Figure 1). We identified diagnostic genetic etiologies in 100 of 522 individuals (19.2%): 89 single nucleotide variants (SNVs) (17.0%) and 11 CNVs (2.1%). Among 142 individuals with DEE, we identified genetic etiologies in 45 individuals (31.7%). Of 180 individuals with GGE including IGE, we identified genetic etiologies in 26 individuals (14.4%); genetic etiologies were also identified in 22 of 152 individuals (14.5%) with NAFE and 7 of 48 individuals (14.6%) with combined focal and generalized epilepsy (eFigure 1 in Supplement 1). There was genetic heterogeneity in all groups, and some genes were identified in multiple groups (eFigure 1 in Supplement 1). Diagnostic yield among individuals with prior clinic testing was 18 of 99 individuals (18.2%), similar to those who had no prior testing (82 of 423 individuals [19.4%]; *P* = .79).

### Diagnostic SNVs

**Figure 2.**
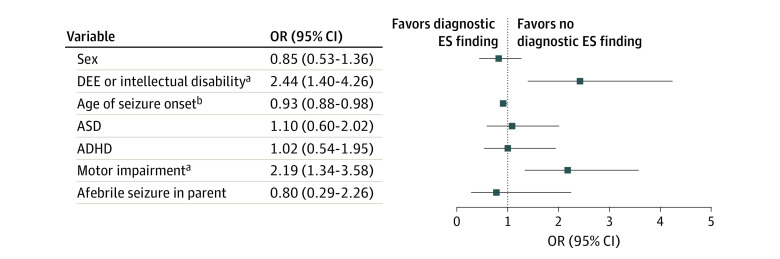
Clinical Features in Patients With Diagnostic Variants Multiple logistic regression analysis of 7 phenotypic variables found that developmental epileptic encephalopathy (DEE) or diagnosis of intellectual disability and history of motor impairment were the strongest factors associated with identifying a diagnostic Exome Sequencing (ES) finding. ADHD indicates attention deficit hyperactivity disorder; ASD, autism spectrum disorder; and OR, odds ratio. ^a^*P* < .001. ^b^*P* < .05.

We initially identified 317 individuals with rare, potentially damaging SNVs. Manual filtering resulted in 89 individuals (17.0%) with a total of 96 variants (including compound heterozygous or homozygous variants) that we classified as diagnostic and returned to families (81 P or LP variants, 15 VUS) (Figure 2 and Table 2). These 89 participants harbored variants (including 43 previously reported) in 69 genes established as associated with epilepsy and neurodevelopmental disorders (eFigure 2 and eTable 1 in Supplement 1). Nine patients had diagnostic variants in *SCN1A* (OMIM: 182389), 3 patients each in *DEPDC5* (OMIM: 614191) and *PRRT2* (OMIM: 614386), 2 patients each in *ANKRD11* (OMIM: 611192), *CHD2* (OMIM: 602119), *GABRG2* (OMIM: 137164), *KCNMA1* (OMIM: 600150), *PCDH19* (OMIM: 300460), *SCN1B* (OMIM: 600235), *STXBP1* (OMIM: 602926), and *SYNGAP1* (OMIM: 603384), and 1 patient each in 58 other genes. Variant types included missense (54 variants [50.5%]), nonsense (16 variants [15.0%]), frameshift (13 variants [12.2%]), and splice site affecting (9 variants [8.4%]) (eFigure 3 in Supplement 1). Two variants were present in mosaic form, 1 in *SCN1A* and 1 in *NEXMIF*. Heterozygous apparently de novo variants in genes associated with autosomal dominant conditions and mechanisms comprised 39 variants (40.2%) of our diagnostic variants (eFigure 3 in Supplement 1); 2 of these variants were subsequently identified to be mosaic in a parent. We identified inherited variants in 24 individuals, including 14 autosomal dominant conditions and 15 autosomal recessive conditions.

**Table 2.** Diagnostic Genetic SNVs and CNVs and Their Associated Phenotypes, From a Cohort of 522 Patients With Unexplained Pediatric Epilepsy^a^

Participant ID	Gene or coordinate	Variant or size/type of CNV	Inheritance	ACMG/AMP for SNVs or syndrome/involved genes for CNVs	Age at seizure onset	Sex	Epilepsy type	Syndrome
**SNVs**								
1	*ANKRD11*	c.3039_3045del, p.Asp1013GlufsTer303	De novo	P	14 y	Female	Combined	NR
2		c.7535G>A, p.Arg2512Gln	Unknown	P	1 y	Female	DEE	LGS
3	*ARHGEF9*	c.1285del, p.Glu429LysfsTer19	Unknown	LP	4 mo	Female	DEE	NR
4	*ARID1B*	c.3586C>T, p.Gln1196Ter	De novo	P	8 y	Female	NAFE	NR
5	*ATN1*	c.3193C>T, p.Gln1065Ter	De novo	VUS	10 y	Male	GGE	NR
6	*BCL11A*	c.198C>G, p.His66Gln	Unknown	LP	2 y	Female	GGE	EEM
7	*BRAF*	c.770A>G, p.Gln257Arg	De novo	P	1 y 6 mo	Male	DEE	NR
8	*BRAT1*	c.1925C>A, p.Ala642Glu	Paternal	LP	6 wk	Female	DEE	NR
		c.294dupA, p.Leu99ThrfsTer92	Maternal	P				
9	*BRWD3*	c.4080+1G>A	Maternal	LP	2 y	Male	NAFE	NR
10	*CACNA1A*	c.601C>T, p.Arg201Trp	Unknown	LP	4 y	Female	IGE	CAE
11	*CACNA1G*	c.3568C>T, p.Arg1190Ter	De novo	VUS	5 y	Female	IGE	CAE
12	*CHD2*	c.2876+3_2876+6delAAGT	De novo	LP	1 y	Female	DEE	NR
13		c.3895_3896insC, p.Val1299AlafsTer5	Unknown	P	1 y 10 mo	Male	DEE	NR
14	*CLN8*	c.784G>A, p.Asp262Asn	Unknown	LP	5 y 6 mo	Female	DEE	NR
		c.610C>T, p.Arg204Cys	Maternal	P				
15	*CREBBP*	c.5315T>A, p.Ile1772Asn	Unknown	LP	Infantile	Male	DEE	NR
16	*CSNK2A1*	c.921T>G, p.Tyr307Ter	De novo	LP	9 m	Female	DEE	NR
17	*CSNK2B*	c.557+1G>A	Unknown	LP	2 m	Male	DEE	NR
18	*CYFIP2*	c.2542A>G, p.Met848Val	De novo	LP	4 m	Male	DEE	IESS
19	*DEPDC5*	c.363+1G>A	Paternal	LP	3 y	Male	NAFE	NR
20		c.667A>G, p.Arg223Gly	Maternal	VUS	8 y	Male	NAFE	NR
21		c.1459C>T, p.Arg487Ter	Unknown	P	10 y	Female	GGE	NR
22	*DYNC1H1*	c.5864G>T, p.Gly1955Val	De novo	LP	2 mo	Male	DEE	IESS
23	*EEF1A2*	c.364G>A, p.Glu122Lys	De novo	P	Infantile	Male	DEE	NR
24	*FRRS1L*	c.737_739del, p.Gly246del	Unknown	P	11 mo	Female	DEE	NR
25	*FOXP1*	c.-448G>C	Unknown	VUS	4 y	Male	DEE	LGS
26	*GABRA5*	c.902C>T, p.Thr301Met	De novo	LP	11 y	Female	Combined	NR
27	*GABRG2*	c.1087C>T, p.Arg363Trp	Unknown	LP	1 y	Male	GGE	NR
28		c.542C>A, p.Thr181Asn	Maternal	LP	7 y	Female	NAFE	NR
29	*GFAP*	c.882C>A, p.Cys294Ter	Unknown	VUS	2 y	Male	GGE	NR
30	*GPHN*	c.1471A>T, p.Arg491Ter	Unknown	P	1 y	Male	GGE	NR
31	*GRIA3*	c.1580C>A, p.Ser527Arg	Maternal	VUS	1 y 6 mo	Male	GGE	NR
32	*GRIN2A*	c.1122+1G>C	Unknown	LP	3 y	Male	NAFE	NR
33	*GRIN2B*	c.1843A>T, p.Asn615Tyr	De novo	P	2 mo	Female	DEE	IESS
34	*KCNA2*	c.217C>T, p.Arg73Ter	Unknown	LP	9 y	Male	Combined	NR
35	*KCNMA1*	c.1918C>T, p.Arg640Ter	Paternal	LP	8 y	Female	Combined	NR
36		c.3199A>G, p.Lys1067Glu	De novo	LP	14 y	Female	IGE	JME
37	*KCNQ2*	c.365C>T, p.Ser122Leu	De novo	P	3 d	Female	DEE	NR
38	*KCNQ3*	c.688C>T, p.Arg230Cys	De novo	P	5 y	Female	DEE	NR
39	*KDM4B*	c.719G>A, p.Arg240Gln	De novo	LP	1 y 6 mo	Male	DEE	NR
40	*KDM6B*	c.40C>G, p.Arg14Gly	De novo	VUS	4 mo	Female	GGE	NR
41	*KMT2E*	c.1097_1116del20, p.Glu366ValfsTer4	De novo	P	4 y	Female	IGE	CAE
42	*LGI1*	c.757G>A, p.Ala253Thr	Maternal	VUS	4 y 6 mo	Male	NAFE	SELECTS
43	*MECP2*	c.1200_1243del, p.Pro401Ter	De novo	P	3 y	Female	NAFE	SHE
44	*MTR*	c.2411T>C, p.Ile804Thr	Paternal	VUS	5 mo	Female	DEE	IESS
		c.2472A>T, p.Ala824=	Maternal	VUS				
45	*NBEA*	c.4702dup, p.Val1568GlyfsTer14	De novo	P	1 y 6 mo	Male	DEE	EMATS
46	*NEXMIF*	c.846_849delTGTC, p.V283tfsX20	De novo mosaic	P	5 y	Female	GGE	NR
47	*NPRL2*	c.323_339+19del	Paternal	P	3 mo	Male	NAFE	IESS
48	*OTUD6B*	c.433C>T, p.Arg145Ter	Both	P	9 mo	Male	DEE	IESS
49	*PCDH19*	c.811_825del, p.Gly271_Tyr275del	De novo	LP	9 mo	Female	DEE	NR
50		c.1335C>A, p.Asp445Glu	Maternal	LP	6 mo	Female	DEE	NR
51	*POLR2A*	c.3281C>T, p.Ser1094Phe	De novo	LP	2 y	Male	DEE	NR
52	*PGAP2*	c.823A>G, p.Met275Val	Unknown	LP	5 mo	Male	DEE	IESS
		c.1040C>T, p.Ala347Val	Maternal	LP				
53	*POLG*	c.1760C>T/c.752C>T, p.Pro587Leu/p.Thr251Ile	Maternal	P	5 y	Male	GGE	NR
		c.1703G>C, p.Gly568Ala	Paternal	VUS				
54	*PPP2R5D*	c.592G>A, p.Glu198Lys	Unknown	P	2 y	Male	DEE	NR
55	*PRRT2*	c.870delT, p.Tyr290Ter	De novo	P	5 mo	Female	NAFE	SELIE
56		c.649dup, p.Arg217ProfsTer8	Paternal	P	3 mo	Female	NAFE	NR
57		c.649dup, p.Arg217ProfsTer8	Maternal	P	5 mo	Male	GGE	SELIE
58	*RORA*	c.680del, p.Thr227ArgfsTer80	De novo	P	6 y	Male	Combined	NR
59	*SCN1A*	c.5066T>C, p.Met1689Thr	De novo	LP	3 y	Male	Combined	NR
60		c.5495C>A, p.Ala1832Glu	Maternal mosaic	P	6 mo	Male	DEE	DS
61		c.3429G>C, p.Glu1143Asp	De novo	LP	6 mo	Male	DEE	DS
62		c.664C>T, p.Arg222Ter	Maternal mosaic	P	9 mo	Male	DEE	DS
63		c.4634T>G, p.Ile1545Arg	De novo	P	3 mo	Male	GGE	NR
64		c.2955T>G, p.Asn985Lys	Unknown	LP	6 mo	Female	NAFE	NR
65		c.332T>A, p.Leu111Ter	De novo mosaic	P	11 mo	Male	NAFE	GEFS+
66		c.4057G>A, p.Val1353Ile	Unknown	P	6 mo	Male	DEE	NR
67		c.5606T>C, p.Phe1869Ser	Maternal	VUS	2 y	Male	GGE	NR
68	*SCN1B*	c.363C>G, p.Cys121Trp	Maternal	LP	1 y 6 mo	Female	NAFE	NR
69		c.1A>C, p.Met1?	Paternal	LP	2 y	Female	GGE	NR
70	*SCN8A*	c.3955G>T, p.Ala1319Ser	De novo	P	3 wk	Male	DEE	NR
71	*SETD1A*	c.4268A>G, p.Gln1423Arg	De novo	LP	11 mo	Female	DEE	HHE
72	*SETD1B*	c.5726T>C, p.Ile1909Thr	De novo	LP	5 y	Female	IGE	CAE
73	*SHANK3*	c.3949dupG, p.Val1317GlyfsX28	De novo	P	8 y	Female	DEE	NR
74	*SLC12A5*	c.1052A>G, p.Asn351Ser	Both	LP	3 m	Female	DEE	EIMFS
75	*SON*	c.6888T>G, p.Asp2296Glu	De novo	LP	8 y	Male	DEE	NR
76	*SPATA5*	c.2045C>T, p.Ala682Val	Maternal	LP	3.5 y	Male	DEE	NR
		c.1883A>G, p.Asp628Gly	Paternal	LP				
77	*SPTAN1*	c.6589_6594dupGAGCT, p.Glu2197_Leu2198dup	De novo	P	2 y	Male	DEE	NR
78	*SRCAP*	c.8919del, p.Leu2975Ter	Unknown	P	5 y	Female	GGE	NR
79	*STAG1*	c.1145C>T, p.Thr382Ile	De novo	VUS	1 y 3 mo	Female	NAFE	NR
80	*STXBP1*	c.1652G>A, p.Arg551His	Unknown	P	1 m	Female	DEE	IESS
81		c.847G>A, p.Glu283Lys	Unknown	P	1 y 6 mo	Male	NAFE	NR
82	*SYNGAP1*	c.403C>T, p.Arg135Ter	De novo	P	3 y	Female	DEE	NR
83		c.1630C>T, p.Arg544Ter	Unknown	P	2 y	Male	DEE	NR
84	*TANC2*	c.2326G>T, p.Glu776Ter	De novo	P	8 mo	Female	DEE	IESS, LGS
85	*TCF4*	c.1486+5delG	De novo	LP	11 y	Female	DEE	NR
86	*TRIT1*	c.967C>T, p.Arg323Trp	Both	LP	1 y 6 mo	Female	DEE	NR
87	*UBA5*	c.829G>A, p.Gly277Ser	Paternal	LP	1 y 11 mo	Male	NAFE	NR
		c.1111G>A, p.Ala371Thr	Maternal	P				
88	*WDR26*	c.706C>G, p.Leu236Val	Unknown	VUS	3 y	Male	DEE	NR
89	*ZEB2*	c.3135C>G, p.His1045Gln	Unknown	VUS	2 y 6 mo	Female	GGE	NR
**CNVs**								
90	chr2:166847505-167334456	487 kb deletion	De novo	*SCN1A*	1 y	Male	Combined	NR
91	chr3:11058648-11060634	2 kb deletion	Unknown^27^	*SLC6A1*	1 y 10 mo	Female	GGE	NR
92	chr16:138446-140150	1.7 kb deletion	Unknown	*NPRL3*	4 mo	Male	NAFE	IESS
93	chr22:32121274-32302733	181 kb deletion	Maternal	*DEPDC5*	6 y	Male	NAFE	NR
94	chr22:32193336-32194893	1.5 kb deletion	Maternal	*DEPDC5*	3 y	Male	NAFE	NR
95	chr1:146630894-147415874	785 kb deletion	Paternal	1q21.1 recurrent microdeletion	5 y	Female	IGE	CAE
96	chr15:30896079-32404350	1.5 Mb deletion	De novo	15q13.3 recurrent microdeletion	6 y	Female	GGE	EMA
97	chr16:29674800-30199626	525 kb duplication	De novo	16p11.2 recurrent microduplication	8 mo	Male	NAFE	NR
98	chr16:21964495-22385880	421 kb deletion	Maternal	16p12.1 recurrent microdeletion	4 y	Male	GGE	NR
99	chr16:14960162-16297720	1.3 Mb deletion	Maternal	16p13.11 recurrent microdeletion	14 y	Male	NAFE	NR
100	chr22:18893638-21386351	2.5 Mb deletion	De novo	22q11 deletion syndrome	6 y	Female	GGE	NR

Abbreviations: ACMG/AMP, American College of Medical Genetics and Genomics/Association for Molecular Pathology; ASM, antiseizure medication; CAE, childhood absence epilepsy; CNV, copy number variant; DEE, developmental and epileptic encephalopathy; DS, Dravet syndrome; EEM, epilepsy with eyelid myoclonia; EIMFS, epilepsy of infancy with migrating focal seizures; EMA, epilepsy with myoclonic absences; EMATS, epilepsy with myoclonic-atonic seizures; GEFS+, generalized epilepsy with febrile seizure plus; GGE, genetic generalized epilepsy; HHE, hemiconvulsion-hemiplegia epilepsy syndrome; ID, identification number; IESS, infantile epileptic spasm syndrome; IGE, idiopathic generalized epilepsy; JME, juvenile myoclonic epilepsy; LGS, Lennox-Gastaut syndrome; LP, likely pathogenic; NAFE, nonacquired focal epilepsy; NR, not reported; P, pathogenic; SELECTS, self-limited epilepsy with centrotemporal spikes; SELIE, self-limited infantile epilepsy; SHE, sleep-related hypermotor epilepsy; SNV, single nucleotide variant; VUS, variants of uncertain significance.

^a^ Gene, variant, and ACMG/AMP classification are given for SNVs. Coordinate, size/type of CNV, and syndrome or genes involved are given for CNVs.

#### Nondiagnostic SNVs

An additional 161 individuals (30.8%) had VUS in known epilepsy genes (eTable 2 in Supplement 1) not considered diagnostic due to phenotypic or disease mechanism inconsistency (eTable 3 in Supplement 1). A total of 93 individuals had variants (72 de novo variants) in 101 candidate genes (eFigure 2 and eTable 4 in Supplement 1) not yet implicated in epilepsy but with experimental evidence suggesting a role in brain development (eg, neuronal migration, signaling, or hyperexcitability).^28^

#### Genetics Related to Syndromes

We identified genetic diagnoses in 9 of 46 individuals (20%) with IESS. Of 9 individuals with *SCN1A* P, LP, or VUS variants, 3 had clinical Dravet syndrome; notably, 2 parents harbored these variants in mosaic form. The others had DEE (1 individual), GGE (2 individuals), NAFE (1 individual with a germline variant and 1 individual with a mosaic variant in the proband), and combined epilepsy (1 individuals), all with seizures in the setting of fever or illness. Notably, we observed a range of epilepsy phenotypes for those genes responsible for more than 1 condition, with *SCN1A* associated with all 4 of the aforementioned categories, *DEPDC5* with GGE and NAFE and *PRRT2* with GGE and NAFE.

We iteratively interrogated phenotypic data in our interpretation of VUS, accounting for clinical features relevant to the implicated genes. For example, following detection of compound heterozygous VUS in *PGAP2*, we confirmed hyperphosphatasia through clinical biochemical testing. A homozygous VUS in *SLC12A5* was identified in a patient with epilepsy of infancy with migrating focal seizures.^29,30^ Finally, a VUS in *CLN8* provided an early diagnosis of neuronal ceroid lipofuscinosis, allowing for anticipatory guidance. We designated these VUS as likely diagnostic, given phenotypic features closely associated with the relevant genes.

### Diagnostic CNVs

We identified diagnostic CNVs in 11 individuals (2.1%) (Table 2; eTable 5 in Supplement 1), none of whom had a diagnostic SNV. Four variants were de novo, 5 variants were inherited, and 2 variants were unknown. In a child with refractory epilepsy and intellectual disability, we identified an 181 kb deletion in *DEPDC5* (chromosome 22) inherited from a parent with well-controlled epilepsy without intellectual disability. For the 4 other inherited CNVs, the parents bearing the CNVs were unaffected, consistent with variable penetrance associated with many CNVs.^27,31,32^

### Phenotypes Associated With Genetic Diagnoses

Multivariable analysis demonstrated higher diagnostic yield among individuals with DEE or intellectual disability (adjusted odds ratio [aOR], 2.44 [95% CI, 1.40-4.26]) and motor impairment (aOR, 2.19 [95% CI, 1.34-3.58]) (Figure 2; eTable 6 in Supplement 1). Patients with younger age at onset had more genetic diagnoses: each year of increasing age conferred a 7% reduction in the likelihood of identifying a genetic cause (aOR per 1-year, 0.93 [95% CI, 0.88-0.98]).

### Clinical Utility of Genetic Diagnoses

Data were available to assess clinical utility for 71 of 100 participants with genetic diagnoses (Table 3). For 29 of these patients (40.8%), we observed evidence of impact in a change in treatment or management or a change in prognosis. Genetic diagnosis led to either a discussion of or actual change in treatment, including change in antiseizure medication or implementation of the ketogenic diet, in 27 patients (38.0%). Other management changes included referrals to other specialties due to risk of nonneurological manifestations and weaning of an antiseizure medication after genetic diagnosis suggested a self-resolving epilepsy (eg, *PRRT2*). Five patients (7%) had a striking change in prognosis, including an early and unsuspected diagnosis of neuronal ceroid lipofuscinosis (*CLN8*).

**Table 3.** Clinical Utility of Genetic Diagnoses for Individuals With Pediatric-Onset Epilepsy^a^

Gene	Actions	Epilepsy type
**Change in treatment and management**		
*ANKRD11*	Referred to cardiovascular genetics, who recommended ECHO with follow-up in 1 y	DEE
	Referred to audiology for hearing evaluation	
	Referred to endocrinology for short stature and bone mineralization seen in KBG syndrome	
	Referred to orthopedic surgery for evaluation for potential vertebral abnormalities and scoliosis	
*ARID1B*	Referred for renal ultrasound and hypothyroidism screening	NAFE
	Referred for evaluation for ASD	
*BCL11A*	Referred to hematology for blood smear to assess for possible *BCL11A*-associated bone marrow abnormalities	GGE
*BRAF*	Referred to cardiology for evaluation due to high rate of cardiac abnormalities associated with *BRAF*	DEE
*CREBBP*	Recommended screening for cataracts, kidney, and thyroid abnormalities	DEE
*CSNK2A1, CSNK2B*	Referred to cardiology for baseline evaluation	DEE
*DEPDC5*	Referred for epilepsy surgical evaluation due to high success rate with focal epilepsy in the setting of mTORopathies	NAFE
	Counseled regarding increased risk for SUDEP and discussed monitoring devices	
*KCNMA1*	Monitoring for symptoms of movement disorders (paroxysmal dyskinesia and ataxia)	Combined
*MECP2*	Annual EKG to evaluate for long QT	NAFE
	Regular spine examinations for scoliosis	
	Monitoring for GERD, constipation, signs and symptoms of gallstones	
	Regular cholesterol screening	
*NPRL2*	Referred for epilepsy surgical evaluation due to high success rate with focal epilepsy in the setting of mTORopathies	NAFE
*PGAP2*	Discussed reports of patients who benefit with vitamin B6 and recommended to monitoring for worsening symptoms if vitamin B6 were discontinued; referred to cardiology for EKG	DEE
	Referred to endocrinology	
*SCN1A*	Started cannabidiol and fenfluramine	DEE
	Recommended temperature management (eg, cooling vest when playing outside)	
	Counseled regarding increased risk for SUDEP; family obtained a monitor for sleeping	
*SCN1B*	Referred to cardiology for evaluation for arrhythmias (due to prior association with Brugada syndrome)	NAFE
*SHANK3*	Referred to cardiology for baseline evaluation	DEE
	Referred to nephrology for baseline evaluation	
	Recommended routine ophthalmology evaluations	
*SLC6A1*	Discussion of medications reported effective in this condition for seizures (ie, valproic acid)	GGE
*TCF4*	Discussion of medications reported effective in this condition for seizures (ie, lamotrigine).	DEE
*TRIT1*	Discussion of treatments reported effective in this condition for seizures (ie, ketogenic diet)	DEE
	Referred to cardiology for evaluation.	
**Change in prognosis**		
*CLN8*	Provided diagnosis and counseling regarding the presence of a neurodegenerative disorder.	DEE
*PPP2R5D*	Patient is substantially delayed and not yet walking; counseled that individuals with this diagnosis can develop skills much later than typical	NAFE
*PRRT2*	Change in prognosis: confidence regarding weaning seizure medication and anticipatory guidance regarding possible movement disorder	NAFE
	Explains family history of paroxysmal kinesigenic dyskinesia that was previously undiagnosed/unexplained	

Abbreviations: ASD, autism spectrum disorder; DEE, developmental and epileptic encephalopathy; ECHO, echocardiography; EKG, electrocardiography; GGE, genetic generalized epilepsy; GERD, gastroesophageal reflux disease; NAFE, nonacquired focal epilepsy; SUDEP, sudden unexpected death in epilepsy.

^a^ Includes only patients for whom such discussion is explicitly documented.

Genetic counseling, including reproductive risk counseling, was offered to all families seen at BCH with diagnostic results. This was particularly relevant for families with inherited variants and for the 2 unaffected parents with low-level mosaic variants.

For the 29 participants for whom follow-up data after genetic diagnosis were not available, we noted that 8 (27.6%) had diagnoses across 5 genes associated with clinical management recommendations: *SCN1A* (3 patients), *DEPDC5* (2 patients), *POLG*, *STXBP1*, and *MTR*.

## Discussion

In this cohort study, we report the genetic results for a large, clinically ascertained cohort with previously unexplained pediatric-onset epilepsy. Our research-based ES data analysis included evaluation for both SNVs and CNVs, the latter from ES data, and evaluation for somatic mosaic variants. The overall yield of 19% (17% SNVs, 2% CNVs) encompasses patients with diverse epilepsy syndromes and varying severity. Equally diverse are the 69 genes identified and the pathways implicated for both early and later onset epilepsies, demonstrating the strength of genomewide approaches.^33,34^ *SCN1A* represented the most commonly identified gene, accounting for 9% of our diagnostic findings and associated with a range of phenotypes.

Consistent with prior reports,^16,35^ our diagnostic yield was highest in patients with higher severity, with earlier age at onset, DEE or intellectual disability, and motor impairment. Specifically, diagnostic yield for DEE (32%) was more than twice that for the other groups (14%-15%). IESS was the most common DEE syndrome in our cohort, which may reflect hospitalization rates^16,36,37^ or referral bias. We also demonstrate genetic diagnoses in patients with non-DEE epilepsies, for whom there may be lower suspicion of genetic epilepsy and less clinical urgency. While non-DEE epilepsies (eg, GGE, NAGE) are considered influenced by polygenic factors,^10,38^ we found single-gene explanations for some patients and overlap of genes implicated in the DEE and non-DEE groups.

The ability to return clinically significant results allowed direct translation of our research into the clinical realm and allowed clinicians to conduct follow-up biochemical and imaging studies, as needed, to provide evidence supporting variant pathogenicity. This is imperative, particularly in the interpretation of VUS, which can benefit from additional phenotypic information. We highlight the importance of including all clinically relevant variants, including VUS that may warrant formal reclassification, and the important dynamic aspect of variant classification that incorporates emerging phenotypic, segregation, and functional data.^39,40^

Our continued access to enrolled patients and longitudinal EMR data enabled us to evaluate clinical utility. Delineation of clinical utility of genetic diagnoses in epilepsy is projected to increase testing by clinicians and support reimbursement from payers, thus increasing access to testing for patients. In contrast, we included a patient population with broad epilepsy phenotypes, some of whose insurance had denied coverage for clinical testing and some with low suspicion of genetic etiology. Our evidence of clinical utility in treatment, clinical management, and prognosis support clinical testing for a broad range of epilepsies.^16^ Beyond clinical utility, we noted anecdotally that several families expressed reduced guilt or shame after genetic diagnosis, relief at the end of a diagnostic odyssey that in some cases had lasted several years, and hope for still undiagnosed families that answers were still being explored through research. We advocate for prospective studies of the clinical and personal utility of genetic diagnoses among cohorts with epilepsy to more comprehensively demonstrate their impact.

We enrolled individuals from an academic hospital where patients seek care for new-onset epilepsy as well as long-term care for refractory epilepsy. While there were no overtly unusual features for patients with IGE or self-limited focal epilepsy, it is possible that patients with refractory seizures, who are seen more frequently, had more opportunities to enroll or more questions raised regarding etiology. This may bias our sample toward individuals with identifiable genetic diagnoses, although conversely, our overall yield may have been diminished by inclusion of patients with milder epilepsy phenotypes, such as GGE and NAFE.^7,10^ During the course of this study, we observed variability in genetic testing approval by insurance payers. Increased access to clinical ES, especially for DEE, may have reduced the seizure severity for patients referred for research ES, possibly accounting for our diagnostic yield of 19% being lower than some previous reports.^41^

We recognize the importance of continued evaluation of patients for whom genetic causes were not found. Exome reanalysis should include evaluation for novel genes and mosaic variants. For some patients, particularly those with syndromes suggesting 1 or more specific genes, genetic diagnoses may be identifiable through targeted deep sequencing of specific genes to assess for mosaic variants, or trio genome sequencing to evaluate for intronic, structural, or other types of variants undetectable with standard ES.^42^ Identification of additional patients and ongoing functional studies may ultimately lead to increased certainty regarding candidate genes. While the time required to scrutinize each variant of potential interest may be prohibitive in some settings, we demonstrate the merits of this approach, with referrals to neurogenetics specialists or subspecialty clinics as needed for variant assessment and explanation of results and their implications to patients and families. As technologies continue to evolve, we advocate for continued harmonization between the research and clinical realms for variant interpretation and translation of research findings to achieve diagnostic precision and clinical utility for all patients with unexplained epilepsy. Finally, future research concerning the psychological effects of these sometimes early genetic diagnoses on families will be important to inform future neurogenetics practice.

### Limitations

This study has some limitations. We used exome capture and undertook CNV analysis of the resulting data to identify deletions or duplications. We acknowledge that exome capture may not detect all CNVs and that genome sequencing might be needed to detect variants beyond SNVs. We were also limited in our interpretation by lack of parental data for some patients, but we chose to include all individuals regardless of parental availability. Furthermore, as with a recent study reporting clinical utility of epilepsy panels,^43^ our EMR-based assessment could not accurately determine impact on hospitalization rates, morbidity or mortality, clinical trials eligibility, and avoidance of testing procedures (such as lumbar puncture, magnetic resonance imaging, or electroencephalography).

## Conclusions

In this cohort study, we illustrated the diverse genetic landscape of pediatric-onset epilepsy in a hospital-based cohort, leveraging research-clinical partnerships to incorporate evolving clinical data in phenotyping, implement the most current guidelines with expertise in genomic analysis and variant interpretation, and increase diagnostic yield and clinical utility.

## Notes

Supplement 1.
eMethods.
**eTable 1.** Clinical and Diagnostic Genetic Findings in 89 Patients With SNVs **eTable 2.** A Phenotype-Driven Epilepsy Gene List **eTable 3.** Nondiagnostic VUS in Epilepsy-Related Genes **eTable 4.** Novel Candidate Epilepsy Genes and Their Associated Phenotypes **eTable 5.** Clinical and Diagnostic Genetic Findings in 11 Cases With CNVs **eTable 6.** Bivariate and Multivariate Regression Model of Phenotypic Variables to Diagnostic Yield by ES **eFigure 1.** Diagnostic Yield and Age of Seizure Onset According to Epilepsy Type **eFigure 2.** Established and Candidate Epilepsy Genes in the Study Cohort **eFigure 2.** Variant Types Among the Diagnostic SNVs and CNVs **eFigure 3.** Clinical Features in Patients With Epilepsy Associated With Diagnostic Variants

Supplement 2.
Members of the BCH Neurology Referral and Phenotyping Group

Supplement 3.
Data Sharing Statement
