# Supplementary material for: Utility of Exome Sequencing for Diagnosis in Unexplained Pediatric-Onset Epilepsy
Source: JAMA Netw Open. 2023 Jul 20;6(7):e2324380. doi: 10.1001/jamanetworkopen.2023.24380 (PMC10359957; doi:10.1001/jamanetworkopen.2023.24380)
Supplement: Supplement 1. — eMethods. eTable 1. Clinical and Diagnostic Genetic Findings in 89 Patients With SNVs eTable 2. A Phenotype-Driven Epilepsy Gene List eTable 3. Nondiagnostic VUS in Epilepsy-Related Genes eTable 4. Novel Candidate Epilepsy Genes and Their Associated Phenotypes eTable 5. Clinical and Diagnostic Genetic Findings in 11 Cases With CNVs eTable 6. Bivariate and Multivariate Regression Model of Phenotypic Variables to Diagnostic Yield by ES eFigure 1. Diagnostic Yield and Age of Seizure Onset According to Epilepsy Type eFigure 2. Established and Candidate Epilepsy Genes in the Study Cohort eFigure 2. Variant Types Among the Diagnostic SNVs and CNVs eFigure 3. Clinical Features in Patients With Epilepsy Associated With Diagnostic Variants [file jamanetwopen-e2324380-s001.pdf]

Supplemental Online Content

Koh HY, Smith L, Wiltrout KN, et al; BCH Neurology Referral and Phenotyping Group. Utility of exome sequencing for diagnosis in unexplained pediatric-onset epilepsy. *JAMA Netw Open*. 2023;6(7):e2324380. doi:10.1001/jamanetworkopen.2023.24380

eMethods.
eTable 1. Clinical and Diagnostic Genetic Findings in 89 Patients With SNVs
eTable 2. A Phenotype-Driven Epilepsy Gene List
eTable 3. Nondiagnostic VUS in Epilepsy-Related Genes
eTable 4. Novel Candidate Epilepsy Genes and Their Associated Phenotypes
eTable 5. Clinical and Diagnostic Genetic Findings in 11 Cases With CNVs
eTable 6. Bivariate and Multivariate Regression Model of Phenotypic Variables to Diagnostic Yield by ES
eFigure 1. Diagnostic Yield and Age of Seizure Onset According to Epilepsy Type
eFigure 2. Established and Candidate Epilepsy Genes in the Study Cohort
eFigure 2. Variant Types Among the Diagnostic SNVs and CNVs
eFigure 3. Clinical Features in Patients With Epilepsy Associated With Diagnostic Variants

This supplemental material has been provided by the authors to give readers additional information about their work.

© 2023 Koh HY et al. *JAMA Network Open.*

eMethods

Sample processing and exome sequencing

Proband DNA and, whenever available, parents' DNA from whole blood or buccal swab were extracted as previously described.[1] Samples were sent to GeneDx (Gaithersburg, MD), a Clinical Laboratory Improvement Amendments (CLIA)-certified testing facility, where DNA was extracted and sequenced. Exomes were captured to produce libraries using xGen probes (Integrated DNA Technologies, IO). The coverage, depth, and quality control requirement are described previously[2]: average coverage across the whole exome sequencing (ES) was 100X and >95% of targets were covered at 20X. The exome by GeneDx medical had >99% of targets covered at 20X and an average depth of ~110X. All sequencing data passed specific minimal quality control requirements: thresholds for mapping percent to hg19 (>95%), target coverage at 10X (90%, 97–98% typical), mean target coverage (50X, average 100–120X), duplicate read percentage (<30%, <10% typical), and read-quality metrics (80% Q30). The remaining samples were maintained in the CLIA-certified facility to ensure DNA availability for future clinical confirmation (i.e., Sanger sequencing and targeted amplicon). Whole ES raw data in the form of FASTQ files were generated.

Bioinformatics pipeline

FASTQ files were aligned into hg19 as previously described[2]: once sequencing was completed, the raw whole ES data were returned to Boston Children's Hospital's Amazon Web Services account. The exome datasets were uniformly processed through a standard variant calling pipeline managed by NextCODE in a research environment.[3] Adapters were trimmed using Skewer v0.2.1,[4] FASTQ analysis was performed by FastQC v0.11.7[5] and base quality was calculated using BBMap v37.97.[6] Read alignment, read depth calculation, realignment, recalibration, and variant calling were performed by Sentieon v201808.03[7]: BWA, HSMetricsAlgo, WGSMetricsAlgo, markduplication and Realigner, QualCal, Haplotyper and GVCFtyper. Verifybamid 1.1.3[8] was used to check contamination and GATK 4.1.2.093 to count reads in bins. NextCODE GORpipe 4.3.0[3] converted other variant data to genomically ordered relational (GOR) format and annotated variants with Variant Effect Predictor (VEP) 96.2 and custom tools.[9]

Variant identification

Single nucleotide variants (SNVs) were annotated using Sequence Miner and Gregor (Genuity Science, Ireland). For those with parental segregation data available, we filtered data for *de novo* heterozygous variants and homozygous/compound heterozygous variants (inherited and/or *de novo*) across all genes (Figure 1). Additionally, we curated a list of established epilepsy-associated genes from Online Mendelian Inheritance in Man (OMIM) or the published literature (eTable 2) and conducted an epilepsy-focused evaluation across the entire cohort, including cases without parental data.

SNV identification

We identified rare, potentially clinically relevant variants predicted to be damaging based on the following criteria: (a) previously reported as pathogenic,[10] (b) minor allele frequency (MAF) <0.001% in Genome Aggregation Database (gnomAD, https://gnomad.broadinstitute.org/)[11] for autosomal dominant (AD), <0.1% for autosomal recessive (AR); (c) absence of homozygous (for AR) or hemizygous (for X-linked) variants in gnomAD; (d) location (exonic or splicing regions); (e) genotype quality =99 and mean allele read depth >10; (f) conservation across $\geq$5 species for missense variants; (g) 'deleteriousness' of missense variants: Combined Annotation Dependent Depletion (CADD) score >20[12] and Variant Effect Predictor (VEP) max score >0.9; and (g) for splice site variants, predicted splicing effects ('cryptic splicing variants', SpliceAI delta score $\geq$0.15).[13] We visualized all variants of interest by direct inspection in the Integrative Genomics Viewer[14] and Sequence Miner. We defined the following as damaging variants: 1) loss-of-function (LoF) including stop-gain, frameshift, and altered canonical splice site, 2) deleterious missense, 3) in-frame indels, and 4) cryptic splicing variants. Inheritance patterns were considered alongside the variant for established disease genes.

We defined mosaic variants as those with variant allele fraction (VAF) <0.2 (vs. germline variants, which have VAFs of roughly 0.5).

CNV identification

We identified CNVs using the Genome Analysis Toolkit (GATK) Germline CNV Caller as described.[15] A model was first built with 'cohort mode' using 500 random samples that were sequenced via the same method, and then CNVs were called for each sample with 'case mode'. Rare, potentially clinically relevant variants were then identified as (a) overlapping recurrent CNV syndrome regions[16] or an epilepsy-associated gene, (b) internal cohort AF <0.2% for dominant inheritance, <5% for recessive inheritance, (c) internal cohort homozygous count (for autosomal recessive) or hemizygous count (for X-linked) <6, and (d) exonic deletion or intragenic duplication (i.e., loss-of-function duplication). We assessed inherited and *de novo* variants accounting for *de novo*/autosomal dominant, autosomal recessive, X-linked dominant, and X-linked recessive models through segregation. Inheritance patterns were considered alongside the variant for established disease genes.

© 2023 Koh HY et al. *JAMA Network Open.*

eTables

eTable 1. Clinical and Diagnostic Genetic Findings in 89 Patients With SNVs

Gene	Variant	Inheritance	Zygosity	AF	ACMG/AMP classification (evidence)	Cases	Seizure type(s)	ID	ASD	ASM response*
ANKRD11	c.3039_3045del p.Asp1013GlufsTer303	*de novo*	hetero	N/A	P (PVS1, PS2, PM2)	0783	FIA (motor) tonic	Borderline	N	Refractory
	c.7535G>A p.Arg2512Gln14609	unknown	hetero	N/A	P (PP5, PM1, PM2, PM5, PP3, BP1)	1111	GES, generalized tonic, GTCS	Severe	N	Refractory
ARHGEF9	c.1285del p.Glu429LysfsTer19	unknown	hetero	N/A	LP (PM2, PVS1)	0613	Febrile generalized motor (4m), focal, rare GTC, atonic	Mild	Y	Refractory
ARID1B	c.3586 C>T p.Gln1196Ter	*de novo*	hetero	N/A	P (PVS1, PS2, PM2)	0477	Focal motor to bilateral tonic-clonic	Mild	N	Refractory
ATN1	c.3193C>T p.Gln1065Ter	*de novo*	hetero	N/A	VUS	1024	Generalized myoclonic, GTCS	Mild	N	Refractory
BCL11A	c.198C>G p.His66Gln	unknown	hetero	N/A	LP (PS1, PM2. PP2)	0544	GTCS; generalized absence, typical with eyelid myoclonia	Mild	N	Controlled
BRAF	c.770A>G p.Gln257Arg	*de novo*	hetero	N/A	P (PS2_VS, PS3, PM2)	0857	Motor tonic-clonic	Moderate	N	Controlled
BRAT1	c.1925C>A p.Ala642Glu	paternal	hetero	0.004119	LP (PM3_Strong, PM2, BP4)	0529	FIA (nonmotor) autonomic	Profound	N	Refractory
	c.294dupA p.Leu99ThrfsTer92	maternal	hetero	N/A	P (PVS1, PM3_Strong)					
BRWD3	c.4080+1G>A	maternal	hemi	N/A	LP (PM2, PVS1)	0484	Focal with secondary generalization	Severe	Y	Controlled
CACNA1A	c.601C>T p.Arg201Trp	unknown	hetero	N/A	LP (PM1, PM2, PP2, PP3)	0558	Generalized absence, typical	Borderline	N	Refractory
CACNA1G	c.3568C>T p.Arg1190Ter	*de novo*	hetero	N/A	VUS	0860	Generalized absence, typical	N/A	N	Refractory
CHD2	c.2876+3_2876+6delAAGT	*de novo*	hetero	N/A	LP (PM2, PP3, PS2, PP4)	0421	GTCS; generalized absence, typical with eyelid myoclonia	Mild	Y	Refractory
	c.3895_3896insC p.Val1299AlafsTer5	unknown	hetero	N/A	P (PM2, PVS1, PP4)	0634	GTCS; generalized absence, typical	Mild	N	Refractory
CLN8	c.784G>A p.Asp262Asn	unknown	hetero	0	LP (PM2, PM3, PP3)	0718	GTCS	Mild	Y	Controlled
	c.610C>T p.Arg204Cys	maternal	hetero	0.00003183	P (PM1, PM2, PP2, PP3, PP5)					
CREBBP	c.5315T>A p.Ile1772Asn	unknown	hetero	N/A	LP (PM2, PM1, PP4, PP3)	0564	FIA (motor) to bilateral tonic-clonic	Moderate	N	Controlled
CSNK2A1	c.921T>G p.Tyr307Ter	*de novo*	hetero	N/A	LP (PS2. PM2)	0798	Generalized myoclonic	Moderate	N	Controlled
CSNK2B	c.557+1G>A	unknown	hetero	N/A	LP (PM2, PVS1)	0648	GTCS	Severe	N	Refractory
CYFIP2	c.2542A>G	*de novo*	hetero	N/A	LP (PM2, PP3, PS2)	0759	FES, FIA (nonmotor) behavioral arrest	Borderline	N	Refractory

© 2023 Koh HY et al. *JAMA Network Open.*

	p.Met848Val									
DEPDC5	c.363+1G>A	paternal	hetero	N/A	LP (PM2, PVS1)	0840	FIA (nonmotor) behavioral arrest to bilateral clonic	N/A	N	Controlled
	c.667A>G p.Arg223Gly	maternal	hetero	N/A	VUS	0434	Focal motor to bilateral tonic-clonic	N/A	N	Refractory
	c.1459C>T p.Arg487Ter	unknown	hetero	N/A	P (PVS1, PM2, PP3, PP5)	1107	GTCS	Borderline		Controlled
DYNC1H1	c.5864G>T p.Gly1955Val	*de novo*	hetero	N/A	LP (PM2, PP3, PS2, PP2)	0360	GES	Borderline	Y	Controlled
EEF1A2	c.364G>A p.Glu122Lys	*de novo*	hetero	N/A	P (PM2, PS2_VS, PP3)	0818	Generalized absence, typical; generalized tonic; generalized myoclonic	Severe	N	Controlled
FRRS1L	c.737_739del p.Gly246del	unknown	homo	0.002402	P (PM2, PS1, PM3_S)	0460	Generalized tonic, GTCS	Profound	N	Refractory
FOXP1	c.-448G>C	unknown	hetero	N/A	VUS	0612	FIA (motor) automatisms (oromotor) to bilateral tonic-clonic	Moderate	Y	Refractory
GABRA5	c.902C>T p.Thr301Met	*de novo*	hetero	N/A	LP (PM1, PM2, PM5, PP2, PP3)	1097	Frontal to bilateral tonic-clonic	Mild	N	Controlled
GABRG2	c.1087C>T p.Arg363Trp	unknown	hetero	N/A	LP (PM2, PM5, PP2, PP3, PP5)	0872	GTCS	N/A	N	Controlled
	c.542C>A p.Thr181Asn	maternal	hetero	N/A	LP (PM2, PP2, PP3, PP4)	0976	FA (motor) clonic (mouth), generalized myoclonic (shoulder, eye fluttering)	N/A	N	Refractory
GFAP	c.882C>A p.Cys294Ter	unknown	hetero	0.000004023	VUS	0508	Febrile (2y), generalized convulsive seizures	Mild	N	Controlled
GPHN	c.1471A>T p.Arg491Ter	unknown	hetero	N/A	P (PVS1, PM2, PP3)	0649	Generalized myoclonic; generalized absence, typical	N/A	N	Refractory
GRIA3	c.1580C>A p.Ser527Arg	maternal	hemi	N/A	VUS	0855	Generalized myoclonic (eye fluttering)	Mild		Refractory
GRIN2A	c.1122+1G>C	unknown	hetero	N/A	LP (PVS1, PM2)	0490	Nonmotor eyelid myoclonia	Borderline	N	Controlled
GRIN2B	c.1843A>T p.Asn615Tyr	*de novo*	hetero	N/A	P (PM2, PM5, PM1, PS2)	0796	GES, generalized tonic, generalized myoclonic	Profound	N	Refractory
KCNA2	c.217C>T p.Arg73Ter	unknown	hetero	N/A	LP (PVS1, PM2)	0766	GTCS	N/A	N	Refractory
KCNMA1	c.1918C>T p.Arg640Ter	paternal	hetero	N/A	LP (PM2, PVS1)	0459	GTCS, unknown motor tonic	Mild	N	Refractory
	c.3199A>G p.Lys1067Glu	*de novo*	hetero	N/A	LP (PM2, PS2)	1004	Generalized myoclonic	N/A	N	Controlled
KCNQ2	c.365C>T p.Ser122Leu	*de novo*	hetero	N/A	P (PM2, PS1, PP1, PS2, PS3)	0781	GTCS	Severe	Y	Controlled
KCNQ3	c.688C>T p.Arg230Cys	*de novo*	hetero	N/A	P (PM2, PS1, PP5, PS2)	0628	FIA (motor) tonic (head, eye)	Severe	Y	Refractory
KDM4B	c.719G>A p.Arg240Gln	*de novo*	hetero	N/A	LP (PS2, PM2, PP3)	0479	Generalized atonic (head drop), GTCS	Moderate	N	Refractory
KDM6B	c.40C>G p.Arg14Gly	*de novo*	hetero	N/A	VUS	0914	GTCS	N/A	N	Controlled

© 2023 Koh HY et al. *JAMA Network Open.*

KMT2E	c.1097_1116del20 p.Glu366ValfsTer4	*de novo*	hetero	N/A	P (PM2, PVS1, PS2)	0774	Generalized absence, typical	Borderline	N	Refractory
LGI1	c.757G>A p.Ala253Thr	maternal	hetero	0.00001991	VUS	0463	FIA (nonmotor) behavioral arrest	N/A	N	Controlled
MECP2	c.1200_1243del p.Pro401Ter	*de novo*	hetero	N/A	P (PM2, PVS1, PS2_VS)	0452	FIA (motor) hypermotor (nocturnal)	Severe	N	Refractory
MTR	c.2411T>C p.Ile804Thr	paternal	hetero	0.00001194	VUS	0744	GES	Moderate	N	Controlled
	c.2472A>T p.Ala824=	maternal	hetero	N/A	VUS					
NBEA	c.4702dup p.Val1568GlyfsTer14	*de novo*	hetero	N/A	P (PM2, PVS1, PS2)	0323	GES, generalized tonic, generalized myoclonic	Severe	N	Refractory
NEXMIF	c.846_849delTGTC p.V283tfsX20	*de novo*	mosaic	N/A	P (PM2, PVS1, PS2)	0545	Absence, GTC	Mild	N	Refractory
NPRL2	c.323_339+19del	paternal	hetero	N/A	P (PM2, PVS1, PP4)	0546	Focal motor to bilateral tonic-clonic	Borderline	N	Refractory
OTUD6B	c.433C>T p.Arg145Ter	both	homo	0.000169	P (PVS1, PM3_S)	0679	GES, generalized myoclonic, generalized atonic (head drops)	Severe	N	Refractory
PCDH19	c.811_825del p.Gly271_Tyr275del	*de novo*	hetero	N/A	LP (PM2, PM4, PS2, PP4)	0768	Generalized atonic (drop)	Moderate	Y	Refractory
	c.1335C>A p.Asp445Glu	maternal	hetero	N/A	LP (PM1, PM2, PP2, PP3, PP5)	0720	Febrile (6m), focal dyscognitive, GTC, tonic	Moderate	Y	Refractory
POLR2A	c.3281C>T p.Ser1094Phe	*de novo*	hetero	N/A	LP (PS2_Moderate, PM2, PP3, PP2)	0906	GTCS	Mild	Y	Controlled
PGAP2	c.823A>G p.Met275Val	unknown	hetero	N/A	LP (PM1, PM2, PP2, PP3)	0689	Infantile spasms, GTC, focal, hemibody tonic	Profound	N	Refractory
	c.1040C>T p.Ala347Val	maternal	hetero	0.00002	LP (PM2, PM3, PP2, PP3)					
POLG	c.1760C>T/c.752C>T p.Pro587Leu/p.Thr251Ile	maternal	hetero	0.00152	P (PM2, PP2, PP3, PP5)	0790	GTCS	N/A	N	Controlled
	c.1703G>C p.Gly568Ala	paternal	hetero	N/A	VUS					
PPP2R5D	c.592G>A p.Glu198Lys	unknown	hetero	N/A	P (PM2, PP3, PS3, PS2_VS)	0678	Generalized myoclonic, generalized atonic (head drop)	Severe	N	Refractory
PRRT2	c.870delT p.Tyr290Ter	*de novo*	hetero	N/A	P (PM2, PVS1, PS2)	0700	Focal motor clonic	N/A	N	Controlled
	c.649dup p.Arg217ProfsTer8	paternal	hetero	0.003103	P (PM2, PVS1, PS3, PS2_VS)	0799	FIA (nonmotor) behavioral arrest	N/A	N	Controlled
	c.649dup p.Arg217ProfsTer8	maternal	hetero	0.003103	P (PM2, PVS1, PS3, PS2_VS)	0987	GTCS	N/A	N	Controlled
RORA	c.680del p.Thr227ArgfsTer80	*de novo*	hetero	N/A	P (PM2, PVS1, PS2)	0957	FIA (motor) tonic to clonic	Borderline	N	Refractory
SCN1A	c.5066T>C p.Met1689Thr	*de novo*	hetero	N/A	LP (PM2, PM5, PP2, PS2)	0498	Focal to bilateral tonic-clonic; generalized absence, typical	Mild	N	Controlled
	c.5495C>A p.Ala1832Glu	maternal mosaic	hetero	N/A	P (PM2, PM5, PM1, PS2)	0439	GTCS, FIA (motor) tonic	Severe	Y	Refractory

© 2023 Koh HY et al. *JAMA Network Open.*

	c.3429G>C p.Glu1143Asp	*de novo*	hetero	N/A	LP (PM2, PM5, PS2)	0824	FA (motor) clonic, GTCS	N/A	N	Refractory
	c.664C>T p.Arg222Ter	maternal mosaic	hetero	N/A	P (PM2, PVS1, PS3, PS2)	0839	FIA (nonmotor) behavioral arrest with autonomic features, GTCS	Mild	N	Refractory
	c.4634T>G p.Ile1545Arg	*de novo*	hetero	N/A	P (PM2, PS1, PS2)	0795	GTCS, generalized myoclonic, generalized atonic (drop)	N/A	N	Controlled
	c.2955T>G p.Asn985Lys	unknown	hetero	N/A	LP (PM2, PM5, PP3, PP2)	0806	Focal motor to bilateral tonic-clonic	Borderline	N	Controlled
	c.332T>A p.Leu111Ter	*de novo*	mosaic	N/A	P (PM2, PVS1, PS2)	0755	Focal	N/A	N	Refractory
	c.4057G>A p.Val1353Ile	unknown	hetero	N/A	P (PM1, PM2, PM5, PP2, PP3)	0846	GTCS, generalized myoclonic	Severe	N	Refractory
	c.5606T>C p.Phe1869Ser	maternal	hetero	N/A	VUS	0926	Generalized myoclonic	N/A	N	Refractory
SCN1B	c.363C>G p.Cys121Trp	maternal	hetero	0.00001414	LP (PM2, PP3, PS3, PP1)	0969	Focal motor to bilateral tonic-clonic	N/A	N	Refractory
	c.1A>C p.Met1?	paternal	hetero	N/A	LP (PVS1, PM2)	1056	Complex febrile	N/A		Refractory
SCN8A	c.3955G>T p.Ala1319Ser	*de novo*	hetero	N/A	P (PM2, PS1, PS2)	0417	Generalized tonic clonic seizures, at times with a prolonged tonic phase	Moderate	N	Refractory
SETD1A	c.4268A>G p.Gln1423Arg	*de novo*	hetero	N/A	LP (PS2, PM2, PP2, PP3)	0850	FA (motor) tonic (mouth twitching)	Mild	N	Refractory
SETD1B	c.5726T>C p.Ile1909Thr	*de novo*	hetero	N/A	LP (PM2, PP3, PS3, PS2)	0461	Generalized absence, typical	Borderline	N	Refractory
SHANK3	c.3949dupG p.Val1317GlyfsX28	*de novo*	hetero	N/A	P (PM2, PVS1, PS2)	0530	GTCS	Profound	N	Controlled
SLC12A5	c.1052A>G p.Asn351Ser	both	homo	0.000004023	LP (PM2, PP3, PP2, PM3)	0476	FIA (motor) tonic (eye) to FIA (nonmotor) behavioral arrest	Moderate	Y	Refractory
SON	c.6888T>G p.Asp2296Glu	*de novo*	hetero	N/A	LP (PS2, PM2, PP3, BP1)	0897	GES, generalized tonic	Mild	N	Refractory
SPATA5	c.2045C>T p.Ala682Val	maternal	hetero	N/A	LP (PM2, PP3, PM3_S)	0500	GES, generalized atonic (drop), generalized tonic; GTCS; generalized absence, atypical	Severe	N	Refractory
	c.1883A>G p.Asp628Gly	paternal	hetero	0.003581	LP (PM2, PP3, PM3_S)					
SPTAN1	c.6589_6594dupGAGCT p.Glu2197_Leu2198dup	*de novo*	hetero	N/A	P (PM2, PM4, PM1, PS2)	0647	Generalized absence, typical with eyelid myoclonia	Mild	N	Refractory
SRCAP	c.8919del p.Leu2975Ter	unknown	hetero	N/A	P (PVS1, PM2, PP5)	0488	GTCS; generalized absence, typical	Mild	N	Refractory
STAG1	c.1145C>T p.Thr382Ile	*de novo*	hetero	N/A	VUS	0664	Focal motor to bilateral tonic-clonic	N/A	N	Refractory
STXBP1	c.1652G>A p.Arg551His	unknown	hetero	N/A	P (PM2, PM5, PS2_VS)	0663	GES, GTCS	Moderate	Y	Controlled
	c.847G>A p.Glu283Lys	unknown	hetero	N/A	P (PM2, PP3, PP2, PS2_VS)	0757	Focal to bilateral tonic-clonic	Borderline	N	Controlled

© 2023 Koh HY et al. *JAMA Network Open.*

SYNGAP1	c.403C>T p.Arg135Ter	*de novo*	hetero	0.000004816	P (PM2, PVS1, PS2_VS)	0483	Generalized absence, typical; generalized absence, atypical; generalized myoclonic	Moderate	Y	Refractory
	c.1630C>T p.Arg544Ter	unknown	hetero	N/A	P (PM2, PVS1, PM6)	0738	Generalized absence, typical; generalized myoclonic	Mild	Y	Refractory
TANC2	c.2326G>T p.Glu776Ter	*de novo*	hetero	N/A	P (PM2, PVS1, PS2)	0437	GES, generalized tonic, generalized atonic (drop)	Severe	N	Refractory
TCF4	c.1486+5delG	*de novo*	hetero	N/A	LP (PM2, PS2, PP4)	0772	GTCS	Severe	N	Refractory
TRIT1	c.967C>T p.Arg323Trp	both	homo	0.000009612	LP (PM2, PS1)	0704	Generalized myoclonic	Mild	N	Controlled
UBA5	c.829G>A p.Gly277Ser	paternal	hetero	N/A	LP (PM2, PP3, PM3, PP4)	0754	Focal motor to bilateral tonic-clonic	Profound	N	Refractory
	c.1111G>A p.Ala371Thr	maternal	hetero	0.00188	P (PP3, PS3, PM3_S)					
WDR26	c.706C>G p.Leu236Val	unknown	hetero	N/A	VUS	0858	Focal	Mild	Y	Controlled
ZEB2	c.3135C>G p.His1045Gln	unknown	hetero	N/A	VUS	0851	Febrile GTC (2y), GTC	Moderate	N	Controlled

ACMG/AMP, The American College of Medical Genetics (ACMG)/Association for Molecular Pathology; AF, allele frequency; N/A, not available; ID, intellectual disability; ASD, autism spectrum disorder; ASM, anti-seizure medication; N/A, not applicable; P, pathogenic; LP, likely pathogenic; PM, moderate evidence; PVS, very strong evidence; PP, supporting evidences; VUS, variant of uncertain significance; FIA, focal impaired awareness seizures; GTCS, generalized tonic-clonic seizure; GTC, generalized tonic seizure; GES, generalized epileptic spasm; Y, yes; N, none.
* Refractoriness to ASM was associated with an increased likelihood of identifying a P/LP variant (OR 1.69 [95% CI 1.02 – 2.78], p = 0.04, two-sided).

© 2023 Koh HY et al. *JAMA Network Open.*

eTable 2. A Phenotype-Driven Epilepsy Gene List

Epilepsy genes in the list used in the study. 1,225 genes known to cause epilepsy or seizure phenotype are listed with RefSeq and outcomes. pLI, loss-of-function intolerant score; mis Z, missense Z score.
*Curated haploinsufficiency refers to ClinGen (https://www.clinicalgenome.org) Gene Dosage Sensitivity.
†Variants in constrained epilepsy genes were considered (pLI >0.9 and/or Z score for missense variation >3.09, eTable 3).[11]

Gene	RefSeq	Disease phenotype	Haplo-insufficiency*	pLI	Mis Z
AARS1	NM_001605.3	Developmental And Epileptic Encephalopathy 29	Unknown	N/A	N/A
AASS	NM_005763.4	Hyperlysinemia	Unknown	0.0	0.9
ABAT	NM_020686.6	Gaba-Transaminase Deficiency	Unknown	0.0	0.6
ABCA5	NM_172232.4	Hypertrichosis, congenital generalized, with gingival hyperplasia, 135400 (3) mutation identified in 1 HTC3 patient	Unknown	0.0	0.4
ABCC8	NM_000352.6	Diabetes Mellitus, Transient Neonatal, 2; Diabetes Mellitus, Permanent Neonatal (PNDM); Hyperinsulinemic Hypoglycemia, Familial, 3 (HHF3); Hyperinsulinemic Hypoglycemia, Familial, 1 (HHF1); Hypoglycemia, Leucine-Induced (LIH)	No	0.0	1.9
ACADM	NM_000016.6	Acyl-Coa Dehydrogenase, Medium-Chain, Deficiency Of (ACADMD)	No	0.0	0.5
ACADS	NM_000017.4	Acyl-Coa Dehydrogenase, Short-Chain, Deficiency Of (ACADSD)	Unknown	0.0	0.6
ACADSB	NM_001609.4	2-Methylbutyryl-Coa Dehydrogenase Deficiency	Unknown	0.0	-0.1
ACOX1	NM_004035.7	Peroxisomal Acyl-Coa Oxidase Deficiency	No	0.1	2.0
ACSF3	NM_001243279.3	Combined Malonic and Methylmalonic Aciduria (CMAMMA)	No	0.0	-1.3
ACSL4	NM_001318510.2	Intellectual Developmental Disorder, X-Linked 63 (MRX63); Amme Complex	Yes	1.0	2.6
ACTB	NM_001101.5	Dystonia, Juvenile-Onset; Baraitser-Winter Syndrome 1 (BRWS1)	Unknown	1.0	5.0
ACTG1†	NM_001614.5	Baraitser-Winter Syndrome 2 (BRWS2); Deafness, Autosomal Dominant 20 (DFNA20); Baraitser-Winter Syndrome 2 (BRWS2); Deafness, Autosomal Dominant 20 (DFNA20)	Unknown	0.0	3.2
ACTL6B	NM_016188.5	Intellectual developmental disorder with severe speech and ambulation defects, 618470 (3); Epileptic encephalopathy, early infantile, 76, 618468 (3)	Unknown	0.0	3.3
ACY1	NM_000666.3	Aminoacylase 1 Deficiency	Unknown	0.0	0.1
ADAM22	NM_001324418.2	Epileptic encephalopathy, early infantile, 61, 617933 (3) mutation identified in 1 EIEE61 patient	Unknown	0.3	2.3
ADAR	NM_001111.5	Aicardi-Goutieres Syndrome 6 (AGS6); Dyschromatosis Symmetrica Hereditaria (DSH)	Unknown	0.0	2.3
ADAT3	NM_138422.4	Intellectual Developmental Disorder, Autosomal Recessive 36 (MRT36)	Unknown	0.0	-0.4
ADD3	NM_016824.5	Cerebral palsy, spastic quadriplegic, 3, 617008 (3) fusion with NUP98 in T-ALL	Unknown	1.0	0.3
ADGRG1	NM_201525.4	Polymicrogyria, Bilateral Frontoparietal	No	N/A	N/A
ADGRV1	NM_032119.4	Febrile Seizures, Familial, 4	Unknown	N/A	N/A
ADK	NM_006721.4	Hypermethioninemia Due To Adenosine Kinase Deficiency	Unknown	0.0	1.5
ADNP†	NM_001282531.3	Helsmoortel-van der Aa syndrome, 615873 (3)	Yes	1.0	2.1
ADPRHL2	NM_017825.3	Neurodegeneration, childhood-onset, stress-induced, with variable ataxia and seizures, 618170 (3)	Unknown	0.0	0.3
ADRA2B†	NM_000682.7	Epilepsy, myoclonic, familial adult, 2, 607876	Unknown	0.0	0.4
ADSL	NM_000026.4	Adenylosuccinase Deficiency	No	0.0	0.8
AFF3	NM_001386135.1	Intellectual disability;Seizures;KINSSHIP syndrome	Unknown	1.0	1.7
AFG3L2	NM_006796.3	Spastic Ataxia 5, Autosomal Recessive (SPAX5); Spinocerebellar Ataxia 28 (SCA28)	Unknown	0.0	2.0
AGA	NM_000027.4	Aspartylglucosaminuria (AGU)	Unknown	0.0	-0.4
AGMO	NM_001004320.2	microcephaly;intellectual disability;epilepsy	Unknown	0.0	-2.6
AGTR2	NM_000686.5	Intellectual Developmental Disorder, X-Linked 88 (MRX88)	Unknown	0.0	-0.1
AHI1	NM_001134831.2	Joubert Syndrome 3 (JBTS3)	No	0.0	0.0
AIFM1	NM_004208.4	Cowchock Syndrome (COWCK); Combined Oxidative Phosphorylation Deficiency 6 (COXPD6)	Unknown	1.0	2.5
AIMP1	NM_001142416.2	Leukodystrophy, Hypomyelinating, 3 (HLD3)	No	0.0	-0.2
AIMP2	NM_006303.4	Leukodystrophy, hypomyelinating, 17, 618006 (3)	Unknown	0.0	-0.8

© 2023 Koh HY et al. *JAMA Network Open.*

AKT1†	NM_001382430.1	Cowden Syndrome 6 (CWS6); Proteus Syndrome; Colorectal Cancer (CRC); Breast Cancer	Unknown	1.0	3.5
AKT2	NM_001626.6	Hypoinsulinemic Hypoglycemia with Hemihypertrophy (HIHGHH); Diabetes Mellitus, Noninsulin-Dependent (NIDDM)	Unknown	0.6	2.4
AKT3	NM_005465.7	Megalencephaly-Polymicrogyria-Polydactyly-Hydrocephalus Syndrome	Uncertain	1.0	3.9
ALDH18A1	NM_002860.4	Cutis Laxa, Autosomal Recessive, Type Iiia (ARCL3A)	Unknown	0.0	2.0
ALDH3A2	NM_000382.3	Sjogren-Larsson Syndrome (SLS)	Unknown	0.0	0.7
ALDH4A1	NM_003748.4	Hyperprolinemia, Type Ii (HPII)	No	0.0	0.0
ALDH5A1	NM_001080.3	Succinic Semialdehyde Dehydrogenase Deficiency (SSADHD)	No	0.0	0.7
ALDH7A1	NM_001182.5	Epilepsy, Pyridoxine-Dependent (EPD)	Unknown	0.0	0.4
ALDOB	NM_000035.4	Fructose Intolerance, Hereditary	Unknown	0.0	-1.4
ALG1	NM_019109.5	Congenital Disorder of Glycosylation, Type Ik (CDG1K)	Unknown	0.0	-2.0
ALG11	NM_001004127.3	Congenital Disorder of Glycosylation, Type Ip (CDG1P)	Unknown	0.0	0.0
ALG13	NM_001099922.3	Congenital Disorder of Glycosylation, Type Is (CDG1S)	Unknown	1.0	1.1
ALG2	NM_033087.4	Congenital Disorder of Glycosylation, Type Ii (CDG1I)	Unknown	0.0	-0.1
ALG3	NM_005787.6	Congenital Disorder of Glycosylation, Type Id (CDG1D)	Unknown	0.0	-0.3
ALG6	NM_013339.4	Congenital Disorder of Glycosylation, Type Ic (CDG1C)	Unknown	0.0	1.5
ALG9	NM_024740.2	Congenital Disorder of Glycosylation, Type Il (CDG1L); Congenital Disorder Of Glycosylation, Type Il (CDG1L); Congenital Disorder Of Glycosylation, Type Il (CDG1L); Congenital Disorder Of Glycosylation, Type Il (CDG1L)	Unknown	0.0	1.0
ALKBH8	NM_138775.3	Intellectual developmental disorder, autosomal recessive 71, 618504 (3)	Unknown	0.0	0.9
ALMS1	NM_001378454.1	Alstrom Syndrome (ALMS)	No	0.0	-3.0
ALPL	NM_000478.6	Hypophosphatasia, Childhood; Hypophosphatasia, Infantile; Hypophosphatasia, Adult	Unknown	0.0	1.3
ALX4†	NM_021926.4	Frontonasal Dysplasia 2 (FND2); Parietal Foramina 2 (PFM2); Potocki-Shaffer Syndrome	Yes	0.4	0.8
AMACR	NM_014324.6	Alpha-Methylacyl-Coa Racemase Deficiency (AMACRD); Bile Acid Synthesis Defect, Congenital, 4 (CBAS4)	Unknown	0.0	0.6
AMER1	NM_152424.4	Osteopathia Striata With Cranial Sclerosis (OSCS)	Unknown	0.9	-0.6
AMPD2	NM_001368809.2	Spastic paraplegia 63, 615686 (3); Pontocerebellar hypoplasia, type 9, 615809 (3) mutation identified in 1 SPG63 family	Unknown	0.0	2.8
AMT	NM_000481.4	Glycine Encephalopathy (GCE)	Unknown	0.0	-0.1
ANK3	NM_020987.5	Intellectual Developmental Disorder, autosomal recessive, 37, 615493 (3)	Unknown	1.0	2.8
ANKH	NM_054027.6	Craniometaphyseal Dysplasia, Autosomal Dominant (CMDD); Chondrocalcinosis 2 (CCAL2)	Unknown	0.3	2.3
ANKLE2	NM_015114.3	Microcephaly 16, primary, autosomal recessive, 616681 (3)	Unknown	0.0	0.3
ANKRD11†	NM_013275.6	KBG Syndrome (KBGS)	Yes	1.0	-0.6
AP1S2	NM_001272071.2	Intellectual Developmental Disorder, X-Linked, Syndromic, Fried Type (MRXSF)	Yes	0.9	2.0
AP2M1†	NM_004068.4	Intellectual developmental disorder 60 with seizures, 618587 (3)	Unknown	1.0	4.9
AP3B2	NM_001278512.2	Epileptic encephalopathy, early infantile, 48, 617276 (3)	Unknown	0.8	2.9
AP4B1	NM_001253852.3	Spastic Paraplegia 47, Autosomal Recessive (SPG47)	No	0.0	0.1
AP4E1	NM_007347.5	Spastic Paraplegia 51, Autosomal Recessive (SPG51)	Unknown	0.0	0.5
AP4M1	NM_004722.4	Spastic Paraplegia 50, Autosomal Recessive (SPG50)	Unknown	0.0	-1.2
AP4S1	NM_001128126.3	Spastic Paraplegia 52, Autosomal Recessive (SPG52)	No	0.0	0.0
APC2	NM_005883.3	Sotos syndrome 3, 617169 (3); Cortical dysplasia, complex, with other brain malformations 10, 618677 (3) mutation identified in 1 SOTOS3 family	Unknown	1.0	0.8
APP	NM_000484.4	Cerebral amyloid angiopathy, Dutch, Italian, Iowa, Flemish, Arctic variants, 605714 (3); Alzheimer disease 1, familial, 104300 (3) proximal to SOD; very distal q21 or boundary with q22	Unknown	0.0	1.4
AQP2	NM_000486.6	Diabetes Insipidus, Nephrogenic, Autosomal	Unknown	0.0	1.1
ARFGEF2	NM_006420.3	Periventricular Heterotopia with Microcephaly, Autosomal Recessive	No	1.0	2.6
ARG1	NM_000045.4	Argininemia	No	0.0	0.1
ARHGAP31†	NM_020754.4	Adams-Oliver Syndrome 1 (AOS1)	Unknown	1.0	0.8
ARHGEF6	NM_004840.3	Intellectual Developmental Disorder, X-Linked 46 (MRX46)	Unknown	1.0	1.3

© 2023 Koh HY et al. *JAMA Network Open.*

ARHGEF9	NM_001353921.2	Epileptic Encephalopathy, Early Infantile, 8 (EIEE8); Epileptic Encephalopathy, Early Infantile, 8 (EIEE8)	Unknown	1.0	3.0
ARID1A†	NM_006015.6	Intellectual Developmental Disorder, Autosomal Dominant 14 (MRD14)	Yes	1.0	3.7
ARID1B†	NM_001374828.1	Intellectual Developmental Disorder, Autosomal Dominant 12 (MRD12)	Yes	1.0	2.6
ARL13B	NM_001174150.2	Joubert Syndrome 8 (JBTS8)	Unknown	0.0	-0.1
ARNT2	NM_014862.4	Webb-Dattani syndrome, 615926 (3) mutation identified in 1 WEDAS family	Unknown	1.0	2.0
ARSA	NM_000487.6	Multiple Sulfatase Deficiency (MSD); Metachromatic Leukodystrophy	No	0.0	0.4
ARV1	NM_022786.3	Epileptic encephalopathy, early infantile, 38, 617020 (3)	Unknown	0.0	0.2
ARX	NM_139058.3	Partington X-Linked Intellectual Developmental Disorder Syndrome (PRTS); Epileptic Encephalopathy, Early Infantile, 1 (EIEE1); Intellectual Developmental Disorder, X-Linked, With or Without Seizures, Arx-Related; Lissencephaly, X-Linked, 2 (LISX2); Corpus Callosum, Agenesis Of, With Abnormal Genitalia	Yes	0.9	2.1
ASAH1	NM_177924.5	Farber lipogranulomatosis, 228000 (3); Spinal muscular atrophy with progressive myoclonic epilepsy, 159950 (3)	Unknown	0.0	-2.2
ASCL1	NM_004316.4	Central hypoventilation syndrome, congenital, 209880 (3); Haddad syndrome, 209880 (3) distal to PAH and proximal to TRA1	Unknown	0.7	1.6
ASL	NM_000048.4	Argininosuccinic Aciduria	Unknown	0.0	0.7
ASNS	NM_001673.5	Asparagine synthetase deficiency, 615574 (3) temperature sensitive G1 mutant	Unknown	0.0	1.9
ASPA	NM_000049.4	Canavan Disease	No	0.0	0.5
ASPM	NM_018136.5	Microcephaly 5, Primary, Autosomal Recessive (MCPH5)	No	0.0	-0.7
ASS1	NM_054012.4	Citrullinemia, Classic	Unknown	0.0	0.8
ASTN1	NM_004319.3	Intellectual disability;epilepsy;cortical malformations	Unknown	1.0	1.7
ASXL1†	NM_015338.6	Myelodysplastic Syndrome (MDS); Bohring-Opitz Syndrome (BOPS)	Yes	0.0	0.6
ATIC	NM_004044.7	Aicar Transformylase/Imp Cyclohydrolase Deficiency	Unknown	0.0	-0.5
ATM	NM_000051.4	Ataxia-Telangiectasia (AT)	Yes	0.0	1.1
ATN1†	NM_001940.4	Dentatorubral-Pallidoluysian Atrophy (DRPLA); Dentatorubral-Pallidoluysian Atrophy (DRPLA)	Unknown	1.0	1.8
ATP13A2	NM_022089.4	Kufor-Rakeb Syndrome (KRS)	Unknown	0.0	1.4
ATP1A1†	NM_000701.8	Charcot-Marie-Tooth disease, axonal, type 2DD, 618036 (3); Hypomagnesemia, seizures, and Intellectual Developmental Disorder 2, 618314 (3)	Unknown	1.0	6.2
ATP1A2†	NM_000702.4	Migraine, Familial Hemiplegic, 2 (FHM2); Alternating Hemiplegia of Childhood 1 (AHC1)	Unknown	0.0	4.8
ATP1A3†	NM_152296.5	Alternating Hemiplegia of Childhood 2 (AHC2); Dystonia 12 (DYT12); Alternating Hemiplegia of Childhood 2 (AHC2); Dystonia 12 (DYT12)	Unknown	1.0	6.3
ATP5F1A	NM_004046.6	Combined Oxidative Phosphorylation Deficiency 22	Unknown	N/A	N/A
ATP6AP2	NM_005765.3	Intellectual Developmental Disorder, X-Linked, Syndromic, Hedera Type (MRXSH)	Unknown	0.9	1.7
ATP6V0A2	NM_012463.4	Wrinkly Skin Syndrome (WSS); Cutis Laxa, Autosomal Recessive, Type Iia (ARCL2A)	No	0.0	0.9
ATP6V1A	NM_001690.4	Cutis laxa, autosomal recessive, type IID, 617403 (3); Epileptic encephalopathy, infantile or early childhood, 3, 618012 (3)	Unknown	0.9	3.4
ATP7A	NM_000052.7	Menkes Disease; Occipital Horn Syndrome (OHS); Spinal Muscular Atrophy, Distal, X-Linked 3 (SMAX3); Menkes Disease; Occipital Horn Syndrome (OHS); Spinal Muscular Atrophy, Distal, X-Linked 3 (SMAX3)	Yes	1.0	1.5
ATP8A2	NM_016529.6	Cerebellar Ataxia, Intellectual Developmental Disorder, And Dysequilibrium Syndrome	Uncertain	0.0	2.3
ATR	NM_001184.4	Cutaneous Telangiectasia and Cancer Syndrome, Familial (FCTCS); Seckel Syndrome 1 (SCKL1)	Uncertain	0.0	4.4
ATRX	NM_000489.6	Intellectual Developmental Disorder -Hypotonic Facies Syndrome, X-Linked, 1 (MRXHF1); Alpha-Thalassemia/ Intellectual Developmental Disorder Syndrome, X-Linked (ATRX); Alpha-Thalassemia Myelodysplasia Syndrome (ATMDS); Intellectual Developmental Disorder -Hypotonic Facies Syndrome, X-Linked, 1 (MRXHF1); Alpha-Thalassemia/ Intellectual Developmental Disorder Syndrome, X-Linked (ATRX); Alpha-Thalassemia Myelodysplasia Syndrome (ATMDS)	Yes	1.0	3.1
ATXN10†	NM_013236.4	Spinocerebellar Ataxia 10 (SCA10)	Unknown	0.0	-0.3
AUH	NM_001698.3	3-Methylglutaconic Aciduria, Type I (MGCA1)	Unknown	0.0	0.3
AUTS2†	NM_015570.4	Intellectual Developmental Disorder, autosomal dominant 26, 615834 (3) translocation break at 7q11.2	Yes	1.0	2.2

© 2023 Koh HY et al. *JAMA Network Open.*

AVPR2	NM_000054.6	Diabetes Insipidus, Nephrogenic, X-Linked; Nephrogenic Syndrome of Inappropriate Antidiuresis (NSIAD); Diabetes Insipidus, Nephrogenic, X-Linked; Nephrogenic Syndrome of Inappropriate Antidiuresis (NSIAD)	Yes	0.6	1.0
B3GALNT2	NM_152490.5	Muscular Dystrophy-Dystroglycanopathy (Congenital with Brain and Eye	Unknown	0.0	1.0
B3GALTL	NM_194318.4	Peters-Plus Syndrome	Unknown	0.0	0.4
B4GAT1	NM_006876.3	Muscular Dystrophy-Dystroglycanopathy (Congenital with Brain and Eye Anomalies), Type A, 13	Unknown	N/A	N/A
B9D1	NM_015681.5	Meckel Syndrome, Type 9 (MKS9)	No	0.0	0.1
BCAP31	NM_001256447.2	Deafness, dystonia, and cerebral hypomyelination, 300475 (3)	Unknown	0.4	1.3
BCKDHA	NM_000709.4	Maple Syrup Urine Disease (MSUD)	Unknown	0.0	0.7
BCKDHB	NM_183050.4	Maple Syrup Urine Disease (MSUD)	Unknown	0.0	-0.2
BCKDK	NM_005881.4	Branched-Chain Ketoacid Dehydrogenase Kinase Deficiency (BCKDKD)	Unknown	0.0	1.6
BCL10	NM_003921.5	Mesothelioma, Malignant (MESOM)	Unknown	0.3	1.4
BCL11A†	NM_022893.4	Fetal Hemoglobin Quantitative Trait Locus 5 (HBFQTL5)	Yes	1.0	3.8
BCOR	NM_001123385.2	Microphthalmia, syndromic 2, 300166 (3)	Yes	1.0	1.9
BCORL1	NM_001379451.1	Shukla-Vernon syndrome, 301029 (3)	Unknown	1.0	2.1
BCS1L	NM_001079866.2	Gracile Syndrome; Bjornstad Syndrome (BJS); Mitochondrial Complex Iii Deficiency, Nuclear Type 1 (MC3DN1)	No	0.0	1.6
BMP4	NM_001202.6	Microphthalmia, Syndromic 6 (MCOPS6); Orofacial Cleft 11 (OFC11)	Unknown	1.0	1.0
BOLA3	NM_212552.3	Multiple Mitochondrial Dysfunctions Syndrome 2 (MMDS2)	Unknown	0.1	0.5
BRAF†	NM_001374258.1	Leopard Syndrome 3; Noonan Syndrome 7 (NS7); Lymphoma, Non-Hodgkin, Familial; Lung Cancer; Cardiofaciocutaneous Syndrome 1 (CFC1); Colorectal Cancer (CRC)	Uncertain	1.0	3.7
BRAT1	NM_152743.4	Rigidity And Multifocal Seizure Syndrome, Lethal Neonatal (RMFSL)	No	0.0	-0.6
BRWD3	NM_153252.5	Intellectual Developmental Disorder, X-Linked 93 (MRX93)	Unknown	1.0	4.3
BSCL2	NM_001122955.4	Neuronopathy, Distal Hereditary Motor, Type Va (HMN5A); Spastic Paraplegia 17, Autosomal Dominant (SPG17); Lipodystrophy, Congenital Generalized, Type 2 (CGL2)	Unknown	0.0	0.3
BTD	NM_001370658.1	Biotinidase Deficiency	No	0.0	-0.5
BUB1B	NM_001211.6	Mosaic Variegated Aneuploidy Syndrome 1 (MVA1); Premature Chromatid Separation Trait (PCS)	Unknown	0.0	0.9
C10orf2	NM_001031344.1	Progressive External Ophthalmoplegia With Mitochondrial DNA Deletions,; Sensory Ataxic Neuropathy, Dysarthria, And Ophthalmoparesis (SANDO); Mitochondrial DNA Depletion Syndrome 7 (Hepatocerebral Type) (MTDPS7)	Unknown	0.0	1.6
C12orf57	NM_138425.4	Temtamy Syndrome (TEMTYS); Temtamy Syndrome (TEMTYS)	Unknown	0.0	-0.9
C12orf65	NM_152269.5	Spastic Paraplegia 55, Autosomal Recessive (SPG55); Combined Oxidative Phosphorylation Deficiency 7 (COXPD7)	Unknown	0.2	-0.4
C5orf42	NM_001384732.1	Joubert Syndrome 17 (JBTS17)	No	0.0	1.9
C9orf72	NM_018325.5	Frontotemporal Dementia And/Or Amyotrophic Lateral Sclerosis (FTDALS)	Unknown	0.0	0.2
CA8	NM_004056.6	Cerebellar ataxia and Intellectual Developmental Disorder with or without quadrupedal locomotion 3, 613227 (3)	No	0.0	1.2
CACNA1A†	NM_001127222.2	Spinocerebellar Ataxia 6 (SCA6); Migraine, Familial Hemiplegic, 1 (FHM1); Episodic Ataxia, Type 2 (EA2)	Yes	1.0	5.8
CACNA1B†	NM_000718.4	Neurodevelopmental disorder with seizures and nonepileptic hyperkinetic movements, 618497 (3)	Unknown	1.0	4.5
CACNA1D	NM_001128840.3	Sinoatrial node dysfunction and deafness, 614896 (3); Primary aldosteronism, seizures, and neurologic abnormalities, 615474 (3)	Unknown	1.0	4.6
CACNA1E†	NM_001205293.3	Epileptic encephalopathy, early infantile, 69, 618285 (3)	Unknown	1.0	5.8
CACNA1G†	NM_018896.5	Spinocerebellar ataxia 42, 616795 (3); Spinocerebellar ataxia 42, early-onset, severe, with neurodevelopmental deficits, 618087 (3)	Unknown	1.0	4.6
CACNA2D2	NM_006030.4	Cerebellar atrophy with seizures and variable developmental delay, 618501 (3)	Unknown	1.0	3.0
CACNB4†	NM_000726.5	Episodic Ataxia, Type 5 (EA5); Epilepsy, Idiopathic Generalized, Susceptibility To, 9 (EIG9)	Unknown	0.0	2.7
CACNG2†	NM_006078.5	Intellectual Developmental Disorder, Autosomal Dominant 10 (MRD10)	Unknown	0.9	2.1
CAD	NM_004341.5	Epileptic encephalopathy, early infantile, 50, 616457 (3)	Unknown	1.0	4.3
CALM1	NM_006888.6	Ventricular Tachycardia, Catecholaminergic Polymorphic, 4 (CPVT4)	Unknown	0.9	3.1
CALM2	NM_001743.6	Ventricular Tachycardia, Catecholaminergic Polymorphic, 4 (CPVT4)	Unknown	0.9	2.8

© 2023 Koh HY et al. *JAMA Network Open.*

CALM3	NM_005184.4	Ventricular Tachycardia, Catecholaminergic Polymorphic, 4 (CPVT4)	Unknown	0.9	3.0
CAMK2A	NM_015981.4	Intellectual Developmental Disorder, autosomal dominant 53, 617798 (3); Intellectual Developmental Disorder, autosomal recessive 63, 618095 (3) mutation identified in 1 MRT63 family	Uncertain	1.0	4.7
CAMK2B†	NM_001220.5	Intellectual Developmental Disorder, autosomal dominant 54, 617799 (3)	Unknown	0.7	4.1
CARS2	NM_024537.4	Combined oxidative phosphorylation deficiency 27, 616672 (3)	Unknown	0.0	0.2
CASK	NM_001367721.1	Intellectual Developmental Disorder and Microcephaly with Pontine and Cerebellar Hypoplasia; Fg Syndrome 4 (FGS4)	Yes	1.0	4.3
CASQ2	NM_001232.4	Ventricular Tachycardia, Catecholaminergic Polymorphic, 2 (CPVT2)	Unknown	0.0	-0.2
CASR	NM_000388.4	Epilepsy, Idiopathic Generalized, Susceptibility To, 8 (EIG8); Calcium-Sensing Receptor (CASR); Hypocalcemia, Autosomal Dominant 1 (HYPOC1); Hyperparathyroidism, Neonatal Severe (NSHPT); Hypocalciuric Hypercalcemia, Familial, Type I (HHC1)	Unknown	0.0	3.1
CBS	NM_000071.3	Homocystinuria Due to Cystathionine Beta-Synthase Deficiency	No	0.0	0.8
CC2D1A	NM_017721.5	Intellectual Developmental Disorder, Autosomal Recessive 3 (MRT3)	Unknown	0.0	0.9
CC2D2A	NM_001378615.1	Joubert Syndrome 9 (JBTS9); Meckel Syndrome, Type 6 (MKS6); Coach Syndrome	No	0.0	0.6
CCBE1	NM_133459.4	Hennekam Lymphangiectasia-Lymphedema Syndrome	Unknown	0.0	-0.7
CCDC88A	NM_001365480.1	PEHO syndrome-like, 617507 (3) mutation identified in 1 PEHOL family	Unknown	1.0	2.3
CCDC88C	NM_001080414.4	Hydrocephalus, Nonsyndromic, Autosomal Recessive 1 (HYC1)	Unknown	0.0	0.7
CCM2†	NM_031443.4	Cerebral Cavernous Malformations 2 (CCM2)	Unknown	0.0	0.5
CCND2	NM_001759.4	Megalencephaly-polymicrogyria-polydactyly-hydrocephalus syndrome 3, 615938 (3)	Unknown	1.0	2.1
CD96	NM_005816.5	C Syndrome	Unknown	0.0	-0.5
CDC42	NM_001791.4	Takenouchi-Kosaki syndrome, 616737 (3)	Unknown	0.8	3.0
CDH15†	NM_004933.3	Intellectual Developmental Disorder, Autosomal Dominant 3 (MRD3)	Unknown	0.0	-1.0
CDKL5	NM_001323289.2	Epileptic Encephalopathy, Early Infantile, 2 (EIEE2)	Yes	1.0	2.7
CDON†	NM_001378964.1	Holoprosencephaly 11 (HPE11)	Unknown	0.0	0.2
CECR1	NM_001282225.2	Vasculitis, autoinflammation, immunodeficiency, and hematologic defects syndrome, 615688 (3); Sneddon syndrome, 182410 (3) mutation identified in 1 SNEDS family	Unknown	0.0	0.4
CENPE	NM_001813.3	Microcephaly 13, primary, autosomal recessive, 616051 (3) mutation identified in 1 MCPH13 family	Unknown	0.2	1.3
CENPJ	NM_018451.5	Seckel Syndrome 4 (SCKL4); Microcephaly 6, Primary, Autosomal Recessive (MCPH6)	Unknown	0.0	0.2
CEP104	NM_014704.4	Joubert syndrome 25, 616781 (3)	Unknown	0.0	0.4
CEP120	NM_001375405.1	Short-rib thoracic dysplasia 13 with or without polydactyly, 616300 (3); Joubert syndrome 31, 617761 (3)	Unknown	0.0	-0.2
CEP152	NM_001194998.2	Microcephaly 9, Primary, Autosomal Recessive (MCPH9); Seckel Syndrome 5 (SCKL5)	Unknown	0.0	0.0
CEP290	NM_025114.4	Leber Congenital Amaurosis 10 (LCA10); Meckel Syndrome, Type 4 (MKS4); Senior-Loken Syndrome 6 (SLSN6); Joubert Syndrome 5 (JBTS5); Bardet-Biedl Syndrome (BBS)	No	0.0	0.5
CEP41	NM_018718.3	Joubert Syndrome 15 (JBTS15); Joubert Syndrome 15 (JBTS15)	Unknown	0.0	0.3
CEP57	NM_014679.5	Mosaic Variegated Aneuploidy Syndrome 2 (MVA2)	Unknown	0.1	-0.5
CERS1	NM_021267.5	Epilepsy, progressive myoclonic, 8, 616230 (3) mutation identified in 1 EPM8 family	Unknown	0.1	0.8
CERT1	NM_001379029.1	Intellectual Developmental Disorder, Autosomal Dominant 34	Unknown	N/A	N/A
CHAMP1†	NM_032436.4	Intellectual Developmental Disorder, autosomal dominant 40, 616579 (3)	Unknown	1.0	0.1
CHD1†	NM_001270.4	Pilarowski-Bjornsson syndrome, 617682 (3)	Unknown	1.0	4.2
CHD2†	NM_001271.4	Epileptic encephalopathy, childhood-onset, 615369 (3)	Yes	1.0	5.2
CHD7	NM_017780.4	Hypogonadotropic Hypogonadism 5 with or without Anosmia (HH5); Scoliosis, Idiopathic, Susceptibility To, 3 (IS3); Charge Syndrome	Yes	1.0	3.2
CHD8†	NM_001170629.2	Autism, Susceptibility To, 18 (AUTS18)	Yes	1.0	5.9
CHKB	NM_005198.5	Muscular dystrophy, congenital, megaconial type, 602541 (3)	No	0.0	-0.5
CHRNA2†	NM_000742.4	Epilepsy, Nocturnal Frontal Lobe, 4 (ENFL4)	Unknown	0.0	-0.3
CHRNA4†	NM_000744.7	Epilepsy, Nocturnal Frontal Lobe, 1 (ENFL1)	Unknown	0.0	0.3
CHRNB2†	NM_000748.3	Epilepsy, Nocturnal Frontal Lobe, 3 (ENFL3)	Unknown	0.0	2.1
CIC†	NM_001386298.1	Intellectual Developmental Disorder, autosomal dominant 45, 617600 (3)	Unknown	1.0	0.7

© 2023 Koh HY et al. *JAMA Network Open.*

CISD2	NM_001008388.5	Wolfram Syndrome 2 (WFS2)	Unknown	0.4	1.3
CKAP2L	NM_152515.5	Filippi syndrome, 272440 (3)	Unknown	0.0	-0.1
CLCF1	NM_013246.3	Cold-Induced Sweating Syndrome 2 (CISS2)	Unknown	0.9	1.1
CLCN1	NM_000083.3	Myotonia Congenita, Autosomal Recessive; Myotonia Congenita, Autosomal Dominant	Unknown	0.0	0.2
CLCN2†	NM_004366.6	Epilepsy, juvenile myoclonic, susceptibility to, 8, 607628 (3); Epilepsy, juvenile absence, susceptibility to, 2, 607628 (3); Epilepsy, idiopathic generalized, susceptibility to, 11, 607628 (3); Leukoencephalopathy with ataxia, 615651 (3); Hyperaldosteronism, familial, type II, 605635 (3)	Unknown	0.0	0.7
CLCN4	NM_001830.4	Raynaud-Claes syndrome, 300114 (3)	Unknown	1.0	4.5
CLDN16	NM_006580.4	Hypomagnesemia 3, Renal (HOMG3)	Unknown	0.0	-0.1
CLEC7A	NM_197947.3	Aspergillosis, Susceptibility To; Candidiasis, Familial, 4 (CANDF4)	Unknown	0.0	0.1
CLIC2	NM_001289.6	Intellectual Developmental Disorder, X-Linked, Syndromic 32 (MRXS32); Intellectual Developmental Disorder, X-Linked, Syndromic 32 (MRXS32)	Unknown	0.0	1.3
CLN3	NM_001042432.2	Ceroid Lipofuscinosis, Neuronal, 3 (CLN3)	Unknown	0.0	-0.1
CLN5	NM_006493.4	Ceroid Lipofuscinosis, Neuronal, 5 (CLN5)	Unknown	0.0	-0.1
CLN6	NM_017882.3	Ceroid Lipofuscinosis, Neuronal, 6 (CLN6); Ceroid Lipofuscinosis, Neuronal, 4A, Autosomal Recessive (CLN4A)	Unknown	0.0	0.0
CLN8	NM_018941.4	Ceroid Lipofuscinosis, Neuronal, 8, Northern Epilepsy Variant; Ceroid Lipofuscinosis, Neuronal, 8 (CLN8)	Unknown	0.0	-0.8
CLP1	NM_006831.3	Pontocerebellar hypoplasia, type 10, 615803 (3)	Unknown	0.2	2.3
CLPB	NM_001258392.3	3-methylglutaconic aciduria, type VII, with cataracts, neurologic involvement and neutropenia, 616271 (3)	Unknown	0.0	1.1
CLPP	NM_006012.4	Perrault Syndrome 3 (PRLTS3)	No	0.6	1.9
CLTC†	NM_004859.4	Intellectual Developmental Disorder, autosomal dominant 56, 617854 (3) fusion gene with TFE3 in renal adenocarcinoma	Unknown	1.0	7.8
CNKSR2	NM_014927.5	Intellectual Developmental Disorder, X-linked, syndromic, Houge type, 301008 (3) conflicting assignment to chr.1	Unknown	1.0	3.6
CNNM2	NM_017649.5	Hypomagnesemia 6, Renal (HOMG6)	Unknown	1.0	4.4
CNPY3	NM_006586.5	Epileptic encephalopathy, early infantile, 60, 617929 (3)	Unknown	0.4	0.4
CNTN2	NM_005076.5	Epilepsy, myoclonic, familial adult, 5, 615400 (3) 1 family identified with mutation	No	0.0	2.6
CNTNAP2	NM_014141.6	Autism, Susceptibility To, 15 (AUTS15); Cortical Dysplasia-Focal Epilepsy Syndrome	Uncertain	0.0	-0.3
COA8	NM_001370595.2	Mitochondrial Complex Iv Deficiency, Nuclear Type 17	Unknown	N/A	N/A
COG2	NM_007357.3	Congenital disorder of glycosylation, type IIq, 617395 (3) mutation identified in 1 CDG2Q patient	Unknown	0.0	0.9
COG4	NM_015386.3	Congenital Disorder of Glycosylation, Type Iij (CDG2J)	Unknown	0.0	-0.3
COG5	NM_006348.5	Congenital Disorder of Glycosylation, Type Iii (CDG2I)	Unknown	0.0	-0.5
COG6	NM_020751.3	Shaheen Syndrome (SHNS); Congenital Disorder of Glycosylation, Type Iil (CDG2L)	Unknown	0.0	-0.2
COG7	NM_153603.4	Congenital Disorder of Glycosylation, Type Iie (CDG2E)	No	0.0	0.8
COG8	NM_032382.5	Congenital Disorder of Glycosylation, Type Iih (CDG2H)	No	0.0	-0.1
COL18A1	NM_001379500.1	Knobloch Syndrome 1 (KNO1)	Unknown	0.0	-0.8
COL4A1†	NM_001845.6	Brain small vessel disease with or without ocular anomalies, 175780 (3); Angiopathy, hereditary, with nephropathy, aneurysms, and muscle cramps, 611773 (3); Hemorrhage, intracerebral, susceptibility to, 614519 (3); Retinal arteries, tortuosity of, 180000 (3); Microangiopathy and leukoencephalopathy, pontine, autosomal dominant, 618564 (3) mutation identified in 1 RATOR family	Unknown	1.0	3.0
COL4A2†	NM_001846.4	Hemorrhage, Intracerebral, Susceptibility To (ICH); Porencephaly 2 (POREN2)	Unknown	0.0	2.2
COMT†	NM_000754.4	Catechol-O-Methyltransferase (COMT); Alcohol Dependence	Unknown	0.0	0.4
COQ2	NM_001358921.2	Coenzyme Q10 Deficiency, Primary, 1 (COQ10D1)	No	0.0	0.0
COQ4	NM_016035.5	Coenzyme Q10 deficiency, primary, 7, 616276 (3)	Unknown	0.0	0.4
COQ6	NM_182476.3	Coenzyme Q10 Deficiency, Primary, 6 (COQ10D6)	Unknown	0.0	0.6
COQ8A	NM_020247.5	Coenzyme Q10 Deficiency, Primary, 4	Unknown	N/A	N/A
COQ9	NM_020312.4	Coenzyme Q10 Deficiency, Primary, 5 (COQ10D5)	Unknown	0.0	-0.2
COX10	NM_001303.4	Leigh Syndrome (LS); Mitochondrial Complex Iv Deficiency	Unknown	0.1	-0.2

© 2023 Koh HY et al. *JAMA Network Open.*

COX15	NM_078470.6	Cardioencephalomyopathy, Fatal Infantile, Due to Cytochrome C Oxidase; Leigh Syndrome (LS)	No	0.0	0.3
COX7B	NM_001866.3	Aplasia Cutis Congenita, Reticulolinear, With Microcephaly, Facial; Aplasia Cutis Congenita, Reticulolinear, With Microcephaly, Facial	Unknown	0.7	0.4
COX8A	NM_004074.3	Mitochondrial complex IV deficiency, nuclear type 15	Unknown	0.1	0.2
CPA6	NM_020361.5	Febrile Seizures, Familial, 11 (44238); Epilepsy, Familial Temporal Lobe, 5 (ETL5)	Unknown	0.0	-0.5
CPLX1	NM_006651.4	Epileptic encephalopathy, early infantile, 63, 617976 (3)	Unknown	0.8	-0.4
CPOX	NM_000097.7	Coproporphyria, Hereditary (HCP)	Unknown	0.2	1.3
CPS1	NM_001875.5	Carbamoylphosphate synthetase I deficiency, 237300 (3); Pulmonary hypertension, neonatal, susceptibility to, 615371 (3) urea cycle enzyme	Unknown	0.0	1.5
CPT1A	NM_001876.4	Carnitine Palmitoyltransferase I Deficiency	No	0.0	1.6
CPT2	NM_000098.3	Encephalopathy, Acute, Infection-Induced, Susceptibility To, 4 (IIAE4); Carnitine Palmitoyltransferase Ii Deficiency, Lethal Neonatal; Carnitine Palmitoyltransferase Ii Deficiency, Infantile; Carnitine Palmitoyltransferase Ii Deficiency, Late-Onset	Unknown	0.0	0.4
CRADD	NM_003805.5	Intellectual Developmental Disorder, Autosomal Recessive 34 (MRT34)	No	0.9	0.1
CRB2	NM_173689.7	Focal segmental glomerulosclerosis 9, 616220 (3); Ventriculomegaly with cystic kidney disease, 219730 (3)	Unknown	0.0	1.1
CRBN	NM_016302.4	Intellectual Developmental Disorder, Autosomal Recessive 2 (MRT2)	Unknown	0.0	0.0
CREBBP†	NM_004380.3	Rubinstein-Taybi Syndrome 1 (RSTS1)	Yes	1.0	3.9
CRLF1	NM_004750.5	Cold-Induced Sweating Syndrome 1 (CISS1)	Unknown	0.0	0.5
CRX	NM_000554.6	Leber Congenital Amaurosis 7 (LCA7); Retinitis Pigmentosa (RP); Cone-Rod Dystrophy 2 (CORD2)	Unknown	0.5	0.3
CSF1R	NM_001288705.3	Leukoencephalopathy, Diffuse Hereditary, With Spheroids (HDLS)	Unknown	0.1	1.6
CSNK2A1†	NM_177559.3	Okur-Chung neurodevelopmental syndrome, 617062 (3) pseudogene on 11p15	Unknown	1.0	3.7
CSNK2B†	NM_001320.7	Poirier-Bienvenu neurodevelopmental syndrome, 618732 (3)	Unknown	0.9	3.1
CSPP1	NM_001382391.1	Joubert syndrome 21, 615636 (3)	No	0.0	0.7
CSTB	NM_000100.4	Myoclonic Epilepsy of Unverricht and Lundborg	No	0.0	0.0
CTBP1†	NM_001012614.2	Hypotonia, ataxia, developmental delay, and tooth enamel defect syndrome, 617915 (3)	Unknown	1.0	3.3
CTC1	NM_025099.6	Cerebroretinal Microangiopathy With Calcifications and Cysts (CRMCC)	No	0.0	0.5
CTNNA2	NM_001282597.3	Cortical dysplasia, complex, with other brain malformations 9, 618174 (3)	Unknown	0.4	3.6
CTNNB1†	NM_001904.4	Intellectual Developmental Disorder, Autosomal Dominant 19 (MRD19); Salivary Gland Adenoma, Pleomorphic; Ovarian Cancer; Mesothelioma, Malignant (MESOM); Medulloblastoma (MDB); Pilomatrixoma; Colorectal Cancer (CRC)	Yes	1.0	3.8
CTSA	NM_000308.4	Galactosialidosis (GSL)	Unknown	0.0	0.5
CTSD	NM_001909.5	Ceroid Lipofuscinosis, Neuronal, 10 (CLN10)	Unknown	0.0	1.4
CTSF	NM_003793.4	Ceroid Lipofuscinosis, Neuronal, 13 (CLN13)	Unknown	0.0	0.7
CUL4B	NM_001079872.2	Intellectual Developmental Disorder, X-Linked, With Short Stature, Hypogonadism, And	Yes	1.0	3.8
CUX2†	NM_015267.4	Epileptic encephalopathy, early infantile, 67, 618141 (3)	Unknown	1.0	3.2
CXCR4	NM_003467.3	Whim Syndrome	Unknown	0.0	1.7
CYFIP2†	NM_001037333.3	Epileptic encephalopathy, early infantile, 65, 618008 (3)	Unknown	1.0	6.0
CYP27A1	NM_000784.4	Cerebrotendinous Xanthomatosis (CTX)	Unknown	0.0	-0.3
CYP27B1	NM_000785.4	Vitamin D-dependent rickets, type I, 264700 (3)	Unknown	0.0	0.1
CYP2R1	NM_024514.5	Vitamin D Hydroxylation-Deficient Rickets, Type 1B (VDDR1B); Vitamin D Hydroxylation-Deficient Rickets, Type 1B (VDDR1B)	Unknown	0.0	0.5
D2HGDH	NM_152783.5	D-2-Hydroxyglutaric Aciduria 1	Unknown	0.1	0.7
DAG1	NM_004393.6	Muscular Dystrophy-Dystroglycanopathy (Limb-Girdle), Type C, 9 (MDDGC9)	Unknown	1.0	0.6
DARS2	NM_018122.5	Leukoencephalopathy with Brainstem and Spinal Cord Involvement	No	0.0	1.2
DBH	NM_000787.4	Dopamine Beta-Hydroxylase Deficiency, Congenital	No	0.0	-1.1
DBT	NM_001918.5	Maple Syrup Urine Disease (MSUD)	Unknown	0.0	1.0
DCX	NM_001195553.2	Lissencephaly, X-linked, 300067 (3); Subcortical laminal heterotopia, X-linked, 300067 (3)	Yes	0.3	2.7
DDB2	NM_000107.3	Xeroderma pigmentosum, group E, DDB-negative subtype, 278740 (3)	Unknown	0.0	1.2
DDOST	NM_005216.5	Congenital Disorder of Glycosylation, Type Ir (CDG1R)	No	0.0	0.8

© 2023 Koh HY et al. *JAMA Network Open.*

DDX3X	NM_001356.5	Intellectual developmental disorder, X-linked, syndrome, Snijders Blok type, 300958 (3)	Yes	1.0	4.3
DEAF1	NM_021008.4	Vulto-van Silfout-de Vries syndrome, 615828 (3); Neurodevelopmental disorder with hypotonia, impaired expressive language, and with or without seizures, 617171 (3)	Unknown	0.0	1.5
DEGS1	NM_003676.4	Leukodystrophy, hypomyelinating, 18, 618404 (3)	Unknown	0.1	0.8
DENND5A	NM_015213.4	Epileptic encephalopathy, early infantile, 49, 617281 (3)	Unknown	0.1	2.4
DEPDC5†	NM_001242896.3	Epilepsy, familial focal, with variable foci 1, 604364 (3)	Unknown	0.1	2.7
DGUOK	NM_080916.3	Mitochondrial DNA Depletion Syndrome 3 (Hepatocerebral Type) (MTDPS3)	Unknown	0.0	-0.5
DHCR24	NM_014762.4	Desmosterolosis	Unknown	0.0	1.5
DHCR7	NM_001360.3	Smith-Lemli-Opitz Syndrome (SLOS)	No	0.0	-0.5
DHDDS	NM_205861.3	Retinitis Pigmentosa 59 (RP59)	Unknown	0.2	1.1
DHFR	NM_000791.4	Megaloblastic Anemia Due to Dihydrofolate Reductase Deficiency	Unknown	0.0	1.0
DHPS	NM_001930.4	Neurodevelopmental disorder with seizures and speech and walking impairment, 618480 (3)	Unknown	0.0	-0.4
DHX30†	NM_138615.3	Neurodevelopmental disorder with severe motor impairment and absent language, 617804 (3)	Unknown	1.0	5.3
DIAPH1†	NM_005219.5	Deafness, autosomal dominant 1, with or without thrombocytopenia, 124900 (3); Seizures, cortical blindness, microcephaly syndrome, 616632 (3)	Unknown	0.9	1.7
DIS3L2	NM_152383.5	Perlman Syndrome (PRLMNS)	Unknown	0.9	0.9
DLD	NM_000108.5	Dihydrolipoamide Dehydrogenase Deficiency (DLDD)	Unknown	0.0	1.1
DLG3	NM_021120.4	Intellectual Developmental Disorder, X-Linked 90 (MRX90)	Unknown	1.0	2.9
DLG4†	NM_001321075.3	Intellectual developmental disorder 62, 618793 (3)	Unknown	1.0	4.9
DLL1†	NM_005618.4	Neurodevelopmental disorder with nonspecific brain abnormalities and with or without seizures, 618709 (3)	Unknown	1.0	1.8
DMBX1	NM_172225.2	global developmental delay, intellectual disability, and epilepsy	Unknown	0.9	0.8
DMD	NM_004006.3	Muscular Dystrophy, Duchenne Type (DMD); Cardiomyopathy, Dilated, 3B (CMD3B); Muscular Dystrophy, Becker Type (BMD)	Yes	1.0	-2.4
DMXL2	NM_001378457.1	Polyendocrine-polyneuropathy syndrome, 616113 (3); Deafness, autosomal dominant 71, 617605 (3); Epileptic encephalopathy, early infantile, 81, 618663 (3) mutation identified in 1 PEPNS family and 1 DFNA71 family	Unknown	1.0	2.4
DNAJC19	NM_145261.4	3-methylglutaconic aciduria, type V, 610198 (3)	Unknown	0.0	0.9
DNAJC5	NM_025219.3	Ceroid lipofuscinosis, neuronal, 4, Parry type, 162350 (3)	Unknown	0.8	1.8
DNAJC6	NM_001256864.2	Parkinson disease 19a, juvenile-onset, 615528 (3); Parkinson disease 19b, early-onset, 615528 (3)	No	1.0	1.6
DNASE1L3	NM_004944.4	Systemic Lupus Erythematosus 16 (SLEB16)	Unknown	0.0	0.0
DNM1†	NM_004408.4	Epileptic encephalopathy, early infantile, 31, 616346 (3)	Unknown	1.0	5.2
DNM1L	NM_012062.5	Encephalopathy, Lethal, Due to Defective Mitochondrial And Peroxisomal	Unknown	0.0	3.8
DNMT1†	NM_001130823.3	Neuropathy, Hereditary Sensory, Type Ie (HSN1E); Cerebellar Ataxia, Deafness, And Narcolepsy, Autosomal Dominant (ADCADN)	Unknown	1.0	5.0
DNMT3A†	NM_022552.5	Tatton-Brown-Rahman syndrome, 615879 (3); Acute myeloid leukemia, somatic, 601626 (3); Heyn-Sproul-Jackson syndrome, 618724 (3)	Uncertain	0.0	3.4
DOCK6	NM_020812.4	Adams-Oliver Syndrome 2 (AOS2)	Unknown	0.0	1.4
DOCK7	NM_001367561.1	Epileptic encephalopathy, early infantile, 23, 615859 (3)	No	0.0	3.4
DOCK8	NM_203447.4	Intellectual Developmental Disorder, Autosomal Dominant 2 (MRD2); Hyper-Ige Recurrent Infection Syndrome, Autosomal Recessive	Unknown	0.0	-2.5
DOLK	NM_014908.4	Congenital Disorder of Glycosylation, Type Im (CDG1M)	Unknown	0.0	1.0
DPAGT1	NM_001382.4	Myasthenic Syndrome, Congenital, With Tubular Aggregates 2 (CMSTA2); Congenital Disorder of Glycosylation, Type Ij (CDG1J)	No	0.0	1.4
DPM1	NM_003859.3	Congenital Disorder of Glycosylation, Type Ie (CDG1E)	No	0.0	0.4
DPM2	NM_003863.4	Congenital Disorder of Glycosylation, Type Iu (CDG1U)	Unknown	0.3	0.6
DPM3	NM_153741.2	Congenital Disorder of Glycosylation, Type Io (CDG1O)	No	0.2	0.4
DPYD	NM_000110.4	Dihydropyrimidine Dehydrogenase Deficiency	No	0.0	-0.2
DPYS	NM_001385.3	Dihydropyrimidinase Deficiency	Unknown	0.0	0.3
DUSP6	NM_001946.4	Hypogonadotropic Hypogonadism 19 With or Without Anosmia (HH19)	Unknown	1.0	1.2

© 2023 Koh HY et al. *JAMA Network Open.*

DYNC1H1†	NM_001376.5	Intellectual Developmental Disorder, Autosomal Dominant 13 (MRD13); Charcot-Marie-Tooth Disease, Axonal, Type 2O (CMT2O); Spinal Muscular Atrophy, Lower Extremity-Predominant, 1, Autosomal	Unknown	1.0	11.0
DYRK1A†	NM_001347721.2	Intellectual Developmental Disorder, Autosomal Dominant 7 (MRD7)	Unknown	1.0	3.3
EARS2	NM_001083614.2	Combined Oxidative Phosphorylation Deficiency 12 (COXPD12)	No	0.0	0.1
ECHS1	NM_004092.4	Mitochondrial short-chain enoyl-CoA hydratase 1 deficiency, 616277 (3)	Unknown	0.0	0.9
ECM1	NM_004425.4	Lipoid Proteinosis of Urbach And Wiethe	Unknown	0.0	-0.2
EDC3	NM_025083.5	Intellectual Developmental Disorder, autosomal recessive 50, 616460 (3) mutation identified in 1 MRT50 family	Unknown	0.0	2.0
EDN3	NM_207034.3	Hirschsprung Disease, Susceptibility To, 4 (HSCR4); Waardenburg Syndrome, Type 4B (WS4B); Central Hypoventilation Syndrome, Congenital (CCHS)	Unknown	0.0	-0.9
EEF1A2†	NM_001958.5	Intellectual Developmental Disorder, autosomal dominant 38, 616393 (3); Epileptic encephalopathy, early infantile, 33, 616409 (3)	Unknown	1.0	4.8
EFTUD2†	NM_004247.4	Mandibulofacial Dysostosis, Guion-Almeida Type (MFDGA)	Yes	1.0	4.0
EGF	NM_001963.6	Hypomagnesemia 4, renal, 611718 (3) linked to ADH3; cen-ADH3-EGF-IL2-qter	Unknown	0.0	0.9
EHMT1†	NM_024757.5	Kleefstra Syndrome	Yes	1.0	1.2
EIF2AK3	NM_004836.7	Epiphyseal Dysplasia, Multiple, With Early-Onset Diabetes Mellitus	Unknown	0.0	2.0
EIF2B1	NM_001414.4	Leukoencephalopathy With Vanishing White Matter (VWM)	Unknown	0.0	1.4
EIF2B2	NM_014239.4	Leukoencephalopathy With Vanishing White Matter (VWM)	Unknown	0.0	1.0
EIF2B3	NM_020365.5	Leukoencephalopathy With Vanishing White Matter (VWM)	No	0.6	1.0
EIF2B4	NM_001034116.2	Leukoencephalopathy With Vanishing White Matter (VWM)	Unknown	1.0	0.8
EIF2B5	NM_003907.3	Leukoencephalopathy With Vanishing White Matter (VWM)	Unknown	0.1	0.8
EIF2S3	NM_001415.4	MEHMO syndrome, 300148 (3)	Unknown	1.0	3.6
EIF3F	NM_003754.3	Intellectual Developmental Disorder, autosomal recessive 67, 618295 (3)	Unknown	1.0	-0.1
ELOVL4	NM_022726.4	Ichthyosis, Spastic Quadriplegia, And Intellectual Developmental Disorder (ISQMR); Stargardt Disease 3 (STGD3)	Unknown	0.8	1.2
EMC1	NM_015047.3	Cerebellar atrophy, visual impairment, and psychomotor retardation, 616875 (3)	Unknown	0.0	1.4
EMG1	NM_006331.8	Bowen-Conradi Syndrome (BWCNS)	Unknown	0.0	-0.3
EML1	NM_004434.3	Band heterotopia, 600348 (3)	Unknown	1.0	2.5
ENG	NM_001114753.3	Telangiectasia, Hereditary Hemorrhagic, Of Rendu, Osler, And Weber	Yes	1.0	0.9
EOGT	NM_001278689.2	Adams-Oliver Syndrome 4 (AOS4)	Unknown	0.0	-0.1
EP300	NM_001429.4	Rubinstein-Taybi Syndrome 2 (RSTS2)	Yes	1.0	2.0
EPB41L1†	NM_012156.2	Intellectual Developmental Disorder, Autosomal Dominant 11 (MRD11)	Unknown	0.9	1.9
EPCAM	NM_002354.3	Diarrhea 5, with tufting enteropathy, congenital, 613217 (3); Colorectal cancer, hereditary nonpolyposis, type 8, 613244 (3) previously assigned to 4q	No	0.0	-1.4
EPG5	NM_020964.3	Vici Syndrome (VICIS)	Unknown	0.0	1.1
EPM2A	NM_005670.4	Myoclonic Epilepsy of Lafora	No	0.0	-0.3
ERCC1	NM_001983.4	Cerebrooculofacioskeletal Syndrome 4 (COFS4)	Unknown	0.0	0.7
ERCC2	NM_000400.4	Cerebrooculofacioskeletal Syndrome 2 (COFS2); Trichothiodystrophy, Photosensitive (TTDP); Xeroderma Pigmentosum, Complementation Group D (XPD)	Unknown	0.0	0.4
ERCC3	NM_000122.2	Xeroderma Pigmentosum, Complementation Group B (XPB); Trichothiodystrophy, Photosensitive (TTDP)	No	0.0	0.9
ERCC4	NM_005236.3	Fanconi Anemia, Complementation Group Q (FANCQ); Xfe Progeroid Syndrome; Xeroderma Pigmentosum, Complementation Group F (XPF)	Unknown	0.0	-0.8
ERCC5	NM_000123.4	Xeroderma Pigmentosum, Complementation Group G (XPG)	Unknown	0.0	0.1
ERCC6	NM_000124.4	Macular Degeneration, Age-Related, 5 (ARMD5); Uv-Sensitive Syndrome 1 (UVSS1); De Sanctis-Cacchione Syndrome; Cerebrooculofacioskeletal Syndrome 1 (COFS1); Cockayne Syndrome B (CSB)	Unknown	0.0	0.1
ERCC8	NM_000082.4	Uv-Sensitive Syndrome 2 (UVSS2); Cockayne Syndrome A (CSA)	No	0.0	-0.4
ERLIN2	NM_007175.8	Spastic Paraplegia 18, Autosomal Recessive (SPG18)	Unknown	0.0	1.9
ERMARD	NM_018341.3	Periventricular nodular heterotopia 6, 615544 (3)	Uncertain	0.0	-0.3
ETHE1	NM_014297.5	Encephalopathy, Ethylmalonic (EE)	Unknown	0.0	1.0
EXOSC3	NM_016042.4	Pontocerebellar Hypoplasia, Type 1B (PCH1B)	No	0.0	-0.2

© 2023 Koh HY et al. *JAMA Network Open.*

EXT2	NM_207122.2	Potocki-Shaffer Syndrome; Exostoses, Multiple, Type Ii	Yes	0.0	0.6
EZH2†	NM_004456.5	Weaver Syndrome (WVS)	Unknown	1.0	4.7
FA2H	NM_024306.5	Spastic paraplegia 35, autosomal recessive, 612319 (3)	Unknown	0.1	0.5
FADD	NM_003824.4	Infections, Recurrent, With Encephalopathy, Hepatic Dysfunction, And	No	0.5	0.8
FAM111A	NM_001312909.2	Gracile Bone Dysplasia (GCLEB); Kenny-Caffey Syndrome, Type 2 (KCS2)	Unknown	0.0	0.0
FAM126A	NM_032581.4	Leukodystrophy, Hypomyelinating, 5 (HLD5)	Unknown	0.1	1.0
FAM58A	NM_152274.5	Toe Syndactyly, Telecanthus, and Anogenital and Renal Malformations	Unknown	0.9	1.1
FAR1	NM_032228.6	Peroxisomal fatty acyl-CoA reductase 1 disorder, 616154 (3)	Unknown	1.0	3.3
FARS2	NM_006567.5	Combined Oxidative Phosphorylation Deficiency 14 (COXPD14)	No	0.0	0.7
FAS	NM_000043.6	Autoimmune Lymphoproliferative Syndrome (ALPS)	Unknown	0.8	1.2
FASTKD2	NM_001136193.2	Combined oxidative phosphorylation deficiency 44, 618855 (3)	Unknown	0.0	0.7
FAT4	NM_001291303.3	Van Maldergem syndrome 2, 615546 (3); Hennekam lymphangiectasia-lymphedema syndrome 2, 616006 (3)	Unknown	1.0	0.6
FBP1	NM_000507.4	Fructose-1,6-Bisphosphatase Deficiency	No	0.1	0.7
FBXL4	NM_001278716.2	Mitochondrial DNA depletion syndrome 13 (encephalomyopathic type), 615471 (3)	Unknown	0.0	0.6
FBXO11†	NM_001190274.2	Intellectual developmental disorder with dysmorphic facies and behavioral abnormalities, 618089 (3) 2p16	Unknown	1.0	4.4
FBXO31	NM_024735.5	Intellectual Developmental Disorder, autosomal recessive 45, 615979 (3) mutation identified in 1 MRT45 family	Unknown	0.4	2.5
FDFT1	NM_004462.5	Squalene synthase deficiency, 618156 (3)	Unknown	0.0	-3.9
FDXR	NM_024417.5	Auditory neuropathy and optic atrophy, 617717 (3)	Unknown	0.0	0.9
FEZF1	NM_001024613.4	Hypogonadotropic hypogonadism 22, with or without anosmia, 616030 (3)	Unknown	1.0	1.3
FGF12†	NM_004113.6	Developmental and epileptic encephalopathy 47	Unknown	0.6	1.5
FGF17	NM_003867.4	Hypogonadotropic Hypogonadism 20 with or without Anosmia (HH20)	Unknown	1.0	1.9
FGF8	NM_033163.5	Hypogonadotropic Hypogonadism 6 with or without Anosmia (HH6)	Unknown	0.7	1.6
FGFR1†	NM_023110.3	Trigonocephaly 1 (TRIGNO1); Osteoglophonic Dysplasia (OGD); Hypogonadotropic Hypogonadism 2 With or Without Anosmia (HH2); Pfeiffer Syndrome	Unknown	1.0	2.5
FGFR2†	NM_000141.5	Bent Bone Dysplasia Syndrome (BBDS); Scaphocephaly, Maxillary Retrusion, And Intellectual Developmental Disorder; Antley-Bixler Syndrome Without Genital Anomalies or Disordered Steroidogenesis; Lacrimoauriculodentodigital Syndrome (LADD); Beare-Stevenson Cutis Gyrata Syndrome (BSTVS); Crouzon Syndrome; Jackson-Weiss Syndrome (JWS); Pfeiffer Syndrome; Apert Syndrome	Unknown	1.0	2.4
FGFR3	NM_000142.5	Crouzon Syndrome with Acanthosis Nigricans (CAN); Camptodactyly, Tall Stature, And Hearing Loss Syndrome; Cervical Cancer; Muenke Syndrome (MNKES); Testicular Germ Cell Tumor (TGCT); Myeloma, Multiple; Thanatophoric Dysplasia, Type Ii (TD2); Thanatophoric Dysplasia, Type I (TD1); Keratosis, Seborrheic; Nevus, Epidermal; Lacrimoauriculodentodigital Syndrome (LADD); Hypochondroplasia (HCH); Bladder Cancer; Achondroplasia (ACH)	Unknown	0.0	1.3
FIG4	NM_014845.6	Amyotrophic Lateral Sclerosis 11 (ALS11); Charcot-Marie-Tooth Disease, Type 4J (CMT4J); Yunis-Varon Syndrome (YVS)	Unknown	0.0	1.9
FKRP	NM_024301.5	Muscular Dystrophy-Dystroglycanopathy (Congenital with Brain and Eye; Muscular Dystrophy-Dystroglycanopathy (Limb-Girdle), Type C, 5 (MDDGC5); Muscular Dystrophy-Dystroglycanopathy (Congenital with or without impaired intellectual development)	Unknown	0.0	1.8
FKTN	NM_001079802.2	Muscular Dystrophy-Dystroglycanopathy (Congenital Without Intellectual Developmental Disorder); Cardiomyopathy, Dilated, 1X (CMD1X); Muscular Dystrophy-Dystroglycanopathy (Limb-Girdle), Type C, 4 (MDDGC4); Muscular Dystrophy-Dystroglycanopathy (Congenital with Brain and Eye	Unknown	0.0	0.0
FLI1	NM_002017.5	Ewing Sarcoma (ES)	Unknown	1.0	2.4
FLNA	NM_001110556.2	Cardiac Valvular Dysplasia, X-Linked (CVD1); Otopalatodigital Syndrome, Type I (OPD1); Melnick-Needles Syndrome (MNS); Frontometaphyseal Dysplasia (FMD); Otopalatodigital Syndrome, Type Ii (OPD2); Heterotopia, Periventricular, Ehlers-Danlos Variant; Fg Syndrome 2 (FGS2); Terminal Osseous Dysplasia (TOD); Heterotopia, Periventricular, X-Linked Dominant; Intestinal Pseudoobstruction, Neuronal, Chronic Idiopathic, X-Linked; Cardiac Valvular Dysplasia, X-Linked (CVD1); Otopalatodigital Syndrome, Type I (OPD1); Melnick-Needles Syndrome (MNS); Frontometaphyseal Dysplasia (FMD); Otopalatodigital Syndrome, Type Ii (OPD2); Heterotopia, Periventricular, Ehlers-Danlos Variant; Fg Syndrome 2 (FGS2); Terminal Osseous Dysplasia (TOD); Heterotopia, Periventricular, X-Linked Dominant; Intestinal Pseudoobstruction, Neuronal, Chronic Idiopathic, X-Linked	Yes	1.0	3.8

© 2023 Koh HY et al. *JAMA Network Open.*

FLRT3	NM_198391.3	Hypogonadotropic Hypogonadism 21 With or Without Anosmia (HH21)	Unknown	0.2	1.9
FLVCR2	NM_017791.3	Proliferative Vasculopathy and Hydranencephaly-Hydrocephaly Syndrome	No	0.0	0.1
FMN2	NM_020066.5	Intellectual Developmental Disorder, autosomal recessive 47, 616193 (3)	Unknown	1.0	0.3
FMR1	NM_002024.6	Premature Ovarian Failure 1 (POF1); Fragile X Intellectual Developmental Disorder Syndrome; Fragile X Tremor/Ataxia Syndrome (FXTAS); Premature Ovarian Failure 1 (POF1); Fragile X Intellectual Developmental Disorder Syndrome; Fragile X Tremor/Ataxia Syndrome (FXTAS)	Unknown	0.6	3.0
FOLR1	NM_016729.3	Neurodegeneration Due to Cerebral Folate Transport Deficiency	No	0.1	0.7
FOXG1†	NM_005249.5	Rett Syndrome, Congenital Variant	Yes	0.9	3.5
FOXP1†	NM_001349338.3	Intellectual Developmental Disorder with Language Impairment And Autistic Features	Yes	1.0	2.3
FOXRED1	NM_017547.4	Mitochondrial Complex I Deficiency	No	0.0	-0.4
FRRS1L	NM_014334.4	Epileptic encephalopathy, early infantile, 37, 616981 (3)	Unknown	0.0	0.6
FTL	NM_000146.4	Hyperferritinemia-cataract syndrome, 600886 (3); Neurodegeneration with brain iron accumulation 3, 606159 (3); L-ferritin deficiency, dominant and recessive, 615604 (3)	Unknown	0.0	-0.4
FTO	NM_001080432.3	Growth Retardation, Developmental Delay, Coarse Facies, And Early	Unknown	0.0	0.6
FTSJ1	NM_012280.4	Intellectual Developmental Disorder, X-Linked 9 (MRX9); Intellectual Developmental Disorder, X-Linked 9 (MRX9)	Yes	1.0	2.2
FUCA1	NM_000147.5	Fucosidosis	Unknown	0.0	0.8
FUK	NM_145059.3	Congenital disorder of glycosylation with defective fucosylation 2, 618324 (3)	Unknown	0.0	0.4
FUT8	NM_001371533.1	Congenital disorder of glycosylation with defective fucosylation 1, 618005 (3)	Unknown	0.0	2.2
FXYD2	NM_001680.5	Hypomagnesemia 2, Renal (HOMG2)	Unknown	0.1	-0.2
G6PC	NM_000151.4	Glycogen storage disease Ia, 232200 (3)	Unknown	0.0	0.3
GABBR2†	NM_005458.8	Nicotine dependence, susceptibility to, 188890 (3); Nicotine dependence, protection against, 188890 (3); Epileptic encephalopathy, early infantile, 59, 617904 (3); Neurodevelopmental disorder with poor language and loss of hand skills, 617903 (3)	Unknown	1.0	4.6
GABRA1†	NM_001127644.2	Epilepsy, Juvenile Myoclonic, Susceptibility To, 5 (EJM5)	Unknown	0.9	3.1
GABRA2†	NM_000807.4	Alcohol dependence, susceptibility to, 103780 (3); Epileptic encephalopathy, early infantile, 78, 618557 (3)	Unknown	1.0	3.1
GABRA5†	NM_000810.4	Epileptic encephalopathy, early infantile, 79, 618559 (3) 100kb from GABRB3	Unknown	0.9	3.2
GABRB1†	NM_000812.4	Epileptic encephalopathy, early infantile, 45, 617153 (3)	Unknown	1.0	2.7
GABRB2†	NM_001371727.1	Epileptic encephalopathy, infantile or early childhood, 2, 617829 (3)	Unknown	0.8	3.4
GABRB3†	NM_000814.6	Epilepsy, Childhood Absence, Susceptibility To, 5 (ECA5); Gamma-Aminobutyric Acid Receptor, Beta-3 (GABRB3)	Unknown	1.0	3.4
GABRG2†	NM_198904.4	Generalized Epilepsy with Febrile Seizures Plus, Type 3 (GEFSP3); Epilepsy, Childhood Absence, Susceptibility To, 2 (ECA2); Dravet Syndrome; Generalized Epilepsy With Febrile Seizures Plus, Type 1 (GEFSP1)	Uncertain	0.7	3.0
GAD1	NM_000817.3	Cerebral Palsy, Spastic Quadriplegic, 1 (CPSQ1)	Unknown	0.0	2.3
GAL†	NM_015973.5	Epilepsy, familial temporal lobe, 8, 616461 (3) mutation identified in 1 ETL8 family	Unknown	0.0	-0.1
GALC	NM_000153.4	Krabbe Disease	Unknown	0.0	0.2
GAMT	NM_000156.6	Cerebral Creatine Deficiency Syndrome 2 (CCDS2)	No	0.0	-0.1
GATA3	NM_001002295.2	Hypoparathyroidism, Sensorineural Deafness, And Renal Disease (HDR)	Yes	0.9	1.9
GATA6†	NM_005257.6	Atrial Septal Defect 9 (ASD9); Atrioventricular Septal Defect 5 (AVSD5); Pancreatic Agenesis and Congenital Heart Defects (PACHD); Conotruncal Heart Malformations (CTHM); Tetralogy Of Fallot (TOF)	Yes	1.0	1.3
GATAD2B†	NM_020699.4	Intellectual Developmental Disorder, Autosomal Dominant 18 (MRD18); Intellectual Developmental Disorder, Autosomal Dominant 18 (MRD18)	Yes	1.0	3.2
GBA	NM_000157.4	Gaucher disease, type I, 230800 (3); Gaucher disease, type II, 230900 (3); Gaucher disease, type III, 231000 (3); Gaucher disease, type IIIC, 231005 (3); Gaucher disease, perinatal lethal, 608013 (3); Parkinson disease, late-onset, susceptibility to, 168600 (3); Lewy body dementia, susceptibility to, 127750 (3) pseudogene GBAP ~16kb 3' to GBA	No	0.0	1.2
GCDH	NM_000159.4	Glutaric Acidemia I	Unknown	0.0	0.3
GCH1	NM_000161.3	Hyperphenylalaninemia, Bh4-Deficient, B (HPABH4B); Dystonia, Dopa-Responsive (DRD)	Yes	0.9	1.5
GCM2	NM_004752.4	Hypoparathyroidism, Familial Isolated (FIH)	Unknown	0.0	0.7
GCSH	NM_004483.5	Glycine Encephalopathy (GCE)	Unknown	0.0	0.1

© 2023 Koh HY et al. *JAMA Network Open.*

GDI1	NM_001493.3	Intellectual Developmental Disorder, X-Linked 41 (MRX41); Intellectual Developmental Disorder, X-Linked 41 (MRX41)	Unknown	1.0	3.3
GDNF	NM_000514.4	Hirschsprung Disease, Susceptibility To, 3 (HSCR3); Central Hypoventilation Syndrome, Congenital (CCHS)	Unknown	0.2	1.1
GFAP†	NM_002055.5	Alexander Disease	Unknown	0.0	0.9
GFM1	NM_024996.7	Combined Oxidative Phosphorylation Deficiency 1 (COXPD1)	No	0.0	1.3
GFM2	NM_032380.5	Combined oxidative phosphorylation deficiency 39, 618397 (3)	Unknown	0.0	0.8
GJA1	NM_000165.5	Atrioventricular Septal Defect 3 (AVSD3); Oculodentodigital Dysplasia, Autosomal Recessive; Hypoplastic Left Heart Syndrome 1 (HLHS1); Hallermann-Streiff Syndrome (HSS); Syndactyly, Type Iii; Oculodentodigital Dysplasia (ODDD)	Unknown	0.2	1.3
GJC2	NM_020435.4	Lymphedema, Hereditary, Ic; Spastic Paraplegia 44, Autosomal Recessive (SPG44); Leukodystrophy, Hypomyelinating, 2 (HLD2)	Unknown	0.0	1.9
GK	NM_001205019.2	Hyperglycerolemia	Yes	1.0	3.2
GLA	NM_000169.3	Fabry Disease; Fabry Disease	Yes	1.0	1.9
GLB1	NM_000404.4	Mucopolysaccharidosis Type Ivb; Gm1-Gangliosidosis, Type Iii; Gm1-Gangliosidosis, Type Ii; Gm1-Gangliosidosis, Type I	No	0.0	0.8
GLDC	NM_000170.3	Glycine Encephalopathy (GCE)	No	0.0	-1.6
GLI2	NM_001374353.1	Holoprosencephaly 9 (HPE9)	Unknown	1.0	0.8
GLI3†	NM_000168.6	Greig Cephalopolysyndactyly Syndrome (GCPS); Polydactyly, Preaxial Iv; Polydactyly, Postaxial, Type A1 (PAPA1); Pallister-Hall Syndrome (PHS)	Unknown	1.0	0.5
GLRA1	NM_000171.4	Hyperekplexia, Hereditary 1 (HKPX1)	Unknown	0.0	1.4
GLRB	NM_000824.5	Hyperekplexia 2 (HKPX2)	No	0.0	0.8
GLS	NM_014905.5	Epileptic encephalopathy, early infantile, 71, 618328 (3); Infantile cataract, skin abnormalities, glutamate excess, and impaired intellectual development, 618339 (3); Global developmental delay, progressive ataxia, and elevated glutamine, 618412 (3) mutation identified in 1 CASGID patient	Unknown	1.0	3.7
GLUD1†	NM_005271.5	Hyperinsulinemic Hypoglycemia, Familial, 6 (HHF6)	Unknown	0.0	3.1
GLUL	NM_001033044.4	Glutamine Deficiency, Congenital	No	1.0	1.6
GLYCTK	NM_145262.4	D-Glyceric Aciduria	Unknown	0.0	-0.2
GM2A	NM_000405.5	Gm2-Gangliosidosis, Ab Variant	Unknown	0.0	-0.3
GMPPA	NM_013335.4	Alacrima, achalasia, and Intellectual Developmental Disorder syndrome, 615510 (3)	No	0.0	1.2
GMPPB	NM_021971.4	Muscular Dystrophy-Dystroglycanopathy (Limb-Girdle), Type C, 14 (MDDGC14); Muscular Dystrophy-Dystroglycanopathy (Congenital With Intellectual Developmental Disorder),; Muscular Dystrophy-Dystroglycanopathy (Congenital With Brain And Eye	Unknown	0.0	1.0
GNAO1†	NM_020988.3	Epileptic encephalopathy, early infantile, 17, 615473 (3); Neurodevelopmental disorder with involuntary movements, 617493 (3) close to MT1 in mouse	Unknown	1.0	3.2
GNAQ	NM_002072.5	Sturge-Weber Syndrome (SWS); Capillary Malformations, Congenital (CMC)	Unknown	1.0	3.7
GNAS	NM_000516.7	Pseudohypoparathyroidism, Type Ic (PHP1C); Pseudohypoparathyroidism, Type Ib (PHP1B); Acth-Independent Macronodular Adrenal Hyperplasia (AIMAH); Mccune-Albright Syndrome (MAS); Osseous Heteroplasia, Progressive (POH); Colorectal Cancer (CRC); Pseudohypoparathyroidism, Type Ia (PHP1A); Pituitary Adenoma, Growth Hormone-Secreting	Yes	0.7	2.7
GNB1†	NM_002074.5	Intellectual Developmental Disorder, autosomal dominant 42, 616973 (3); Leukemia, acute lymphoblastic, somatic, 613065 (3)	Uncertain	1.0	3.8
GNB5	NM_016194.4	Intellectual developmental disorder with cardiac arrhythmia, 617173 (3); Language delay and ADHD/cognitive impairment with or without cardiac arrhythmia, 617182 (3)	Unknown	0.0	1.5
GNE	NM_005476.7	Nonaka Myopathy (NM); Inclusion Body Myopathy 2, Autosomal Recessive (IBM2); Sialuria	Unknown	0.0	2.6
GNS	NM_002076.4	Mucopolysaccharidosis Type Iiid	Unknown	0.0	1.4
GOSR2	NM_004287.5	Epilepsy, Progressive Myoclonic 6 (EPM6)	Unknown	0.0	-0.5
GOT2	NM_002080.4	Epileptic encephalopathy, early infantile, 82, 618721 (3) pseudogenes on 12 and 1	Unknown	0.0	1.4
GP1BB	NM_000407.5	Bernard-Soulier Syndrome (BSS)	Unknown	0.5	0.5
GPAA1	NM_003801.4	Glycosylphosphatidylinositol biosynthesis defect 15, 617810 (3)	Unknown	0.0	0.9
GPC3	NM_004484.4	Simpson-Golabi-Behmel Syndrome, Type 1 (SGBS1)	Yes	1.0	1.5
GPC4	NM_001448.3	Keipert syndrome, 301026 (3) centromeric to GPC3	Unknown	0.9	1.7
GPHN†	NM_020806.5	Molybdenum cofactor deficiency C, 615501 (3)	Unknown	1.0	3.4

© 2023 Koh HY et al. *JAMA Network Open.*

GPSM2	NM_013296.5	Chudley-Mccullough Syndrome (CMCS)	No	0.0	1.3
GPT2	NM_133443.4	Intellectual Developmental Disorder, autosomal recessive 49, 616281 (3)	Unknown	0.0	1.9
GRIA2†	NM_001083619.3	Neurodevelopmental disorder with language impairment and behavioral abnormalities, 618917 (3)	Unknown	1.0	4.6
GRIA3	NM_007325.5	Intellectual Developmental Disorder, X-Linked, Syndromic, Wu Type (MRXSW)	Unknown	1.0	4.2
GRIA4†	NM_000829.4	Neurodevelopmental disorder with or without seizures and gait abnormalities, 617864 (3)	Unknown	0.0	3.4
GRIK2	NM_021956.5	Intellectual Developmental Disorder, Autosomal Recessive 6 (MRT6)	No	1.0	2.9
GRIN1	NM_007327.4	Intellectual Developmental Disorder, Autosomal Dominant 8 (MRD8)	Unknown	1.0	6.2
GRIN2A†	NM_001134407.3	Epilepsy, focal, with speech disorder and with or without Intellectual Developmental Disorder, 245570 (3)	Uncertain	1.0	2.8
GRIN2B†	NM_000834.5	Intellectual Developmental Disorder, Autosomal Dominant 6 (MRD6)	Yes	1.0	5.4
GRIN2D†	NM_000836.4	Epileptic encephalopathy, early infantile, 46, 617162 (3)	Unknown	1.0	4.8
GRM1	NM_001278064.2	Spinocerebellar Ataxia, Autosomal Recessive 13 (SCAR13)	Unknown	0.1	2.6
GRM7	NM_000844.4	Neurodevelopmental disorder with seizures, hypotonia, and brain imaging abnormalities, 618922 (3)	Unknown	1.0	2.1
GRN	NM_002087.4	Ceroid Lipofuscinosis, Neuronal, 11 (CLN11); Frontotemporal Lobar Degeneration with Tdp43 Inclusions, Grn-Related	Unknown	0.1	0.3
GSS	NM_000178.4	Glutathione Synthetase Deficiency (GSSD); Glutathione Synthetase Deficiency of Erythrocytes, Hemolytic Anemia	Unknown	0.0	0.8
GTPBP2	NM_019096.5	Jaberi-Elahi syndrome, 617988 (3)	Unknown	0.3	3.4
GTPBP3	NM_032620.4	Deafness, Aminoglycoside-Induced	Unknown	0.0	0.0
GUF1	NM_021927.3	Epileptic encephalopathy, early infantile, 40, 617065 (3) mutation identified in 1 EIEE40 family	Unknown	0.0	0.2
GYS1	NM_002103.5	Glycogen Storage Disease 0, Muscle	Unknown	0.0	1.8
GYS2	NM_021957.4	Glycogen Storage Disease 0, Liver	No	0.0	0.1
HACE1	NM_020771.4	Spastic paraplegia and psychomotor retardation with or without seizures, 616756 (3)	Unknown	0.0	3.6
HADH	NM_005327.7	Hyperinsulinemic Hypoglycemia, Familial, 4 (HHF4); 3-Hydroxyacyl-Coa Dehydrogenase Deficiency	Unknown	0.0	0.5
HAX1	NM_006118.4	Neutropenia, Severe Congenital, 3, Autosomal Recessive (SCN3)	Unknown	0.0	0.1
HCCS	NM_005333.5	Microphthalmia, Syndromic 7 (MCOPS7)	Yes	0.9	1.2
HCFC1	NM_005334.3	Methylmalonic Acidemia And Homocysteinemia, Cblx Type; Methylmalonic Acidemia and Homocysteinemia, Cblx Type	Unknown	1.0	5.6
HCN1†	NM_021072.4	Epileptic encephalopathy, early infantile, 24, 615871 (3); Generalized epilepsy with febrile seizures plus, type 10, 618482 (3)	Unknown	1.0	3.7
HCN2	NM_001194.4	Genetic epilepsy with febrile seizures plus;Other seizure disorders	Unknown	0.5	3.4
HDAC4†	NM_001378414.1	Brachydactyly-Intellectual Developmental Disorder Syndrome (BDMR)	Uncertain	1.0	2.9
HDAC8	NM_018486.3	Wilson-Turner X-Linked Intellectual Developmental Disorder Syndrome (WTS); Cornelia De Lange Syndrome 5 (CDLS5); Wilson-Turner X-Linked Intellectual Developmental Disorder Syndrome (WTS); Cornelia De Lange Syndrome 5 (CDLS5)	Yes	1.0	2.8
HECW2†	NM_001348768.2	Neurodevelopmental disorder with hypotonia, seizures, and absent language, 617268 (3)	Unknown	1.0	3.3
HEPACAM	NM_152722.5	Megalencephalic Leukoencephalopathy with Subcortical Cysts 2B, Remitting; Megalencephalic Leukoencephalopathy with Subcortical Cysts 2A (MLC2A)	Unknown	0.9	1.6
HERC2	NM_004667.6	Skin/Hair/Eye Pigmentation, Variation In, 1 (SHEP1); Skin/Hair/Eye Pigmentation, Variation In, 1 (SHEP1)	Unknown	1.0	4.4
HESX1	NM_003865.3	Pituitary Hormone Deficiency, Combined, 2 (CPHD2); Septooptic Dysplasia	Unknown	0.0	-0.2
HEXA	NM_000520.6	Tay-Sachs Disease (TSD)	Unknown	0.0	-0.4
HEXB	NM_000521.4	Sandhoff Disease	Unknown	0.0	0.6
HGSNAT	NM_152419.3	Mucopolysaccharidosis Type Iiic	Unknown	0.0	0.7
HIBCH	NM_014362.4	Beta-Hydroxyisobutyryl Coa Deacylase Deficiency	Unknown	0.0	0.3
HIVEP2†	NM_006734.4	Intellectual Developmental Disorder, autosomal dominant 43, 616977 (3)	Yes	1.0	1.8
HK1	NM_000188.3	Neuropathy, Hereditary Motor and Sensory, Russe Type (HMSNR); Hemolytic Anemia, Nonspherocytic, Due To Hexokinase Deficiency	Unknown	0.9	3.2
HLCS	NM_001352514.2	Holocarboxylase Synthetase Deficiency	No	0.0	0.1

© 2023 Koh HY et al. *JAMA Network Open.*

HMBS†	NM_000190.4	Porphyria, Acute Intermittent; Porphyria, Acute Intermittent	Yes	0.9	0.6
HMGCL	NM_000191.3	3-Hydroxy-3-Methylglutaryl-Coa Lyase Deficiency (HMGCLD)	Unknown	0.0	0.5
HMGCS2	NM_005518.4	3-Hydroxy-3-Methylglutaryl-Coa Synthase 2 Deficiency	Unknown	0.0	-0.2
HNRNPH2	NM_019597.5	Intellectual Developmental Disorder, X-linked, syndromic, Bain type, 300986 (3) second signal at 6q25.3-q26, pseudogene	Unknown	1.0	3.6
HNRNPR	NM_005826.5	Global developmental delay;Intellectual disability;Seizures;Postnatal microcephaly;Short digit	Unknown	1.0	3.5
HNRNPU	NM_031844.3	Epileptic encephalopathy, early infantile, 54, 617391 (3)	Unknown	1.0	3.4
HOXA1	NM_005522.5	Bosley-Salih-Alorainy syndrome, 601536 (3); Athabaskan brainstem dysgenesis syndrome, 601536 (3)	Unknown	0.3	-0.1
HPD	NM_002150.3	Tyrosinemia, Type Iii; Hawkinsinuria	Unknown	0.0	0.7
HPRT1	NM_000194.3	Kelley-Seegmiller Syndrome; Lesch-Nyhan Syndrome (LNS)	Yes	0.9	2.3
HRAS	NM_005343.4	Thyroid Carcinoma, Hurthle Cell; Costello Syndrome; Schimmelpenning-Feuerstein-Mims Syndrome (SFM); Bladder Cancer	Unknown	0.1	1.5
HSD17B10	NM_004493.3	Intellectual Developmental Disorder, X-Linked 17 (MRX17); 17-Beta-Hydroxysteroid Dehydrogenase X Deficiency; Intellectual Developmental Disorder, X-Linked, Syndromic 10 (MRXS10); Intellectual Developmental Disorder, X-Linked 17 (MRX17); 17-Beta-Hydroxysteroid Dehydrogenase X Deficiency; Intellectual Developmental Disorder, X-Linked, Syndromic 10 (MRXS10)	Unknown	0.9	2.6
HSD17B4	NM_000414.4	D-Bifunctional Protein Deficiency; Perrault Syndrome 1 (PRLTS1)	No	0.0	-0.3
HSPD1	NM_002156.5	Leukodystrophy, Hypomyelinating, 4 (HLD4); Spastic Paraplegia 13, Autosomal Dominant (SPG13)	Unknown	1.0	2.3
HTRA1	NM_002775.5	Macular degeneration, age-related, 7, 610149 (3); Macular degeneration, age-related, neovascular type, 610149 (3); CARASIL syndrome, 600142 (3); Cerebral arteriopathy, autosomal dominant, with subcortical infarcts and leukoencephalopathy, type 2, 616779 (3)	Unknown	0.0	1.1
HTRA2	NM_013247.5	Parkinson disease 13, 610297 (3); 3-methylglutaconic aciduria, type VIII, 617248 (3)	Unknown	0.0	1.7
HUWE1	NM_031407.7	Intellectual Developmental Disorder, X-Linked, Syndromic, Turner Type (MRXST); Intellectual Developmental Disorder, X-Linked 17 (MRX17); Intellectual Developmental Disorder, X-Linked, Syndromic, Turner Type (MRXST); Intellectual Developmental Disorder, X-Linked 17 (MRX17)	Unknown	1.0	8.9
HYLS1	NM_001134793.2	Hydrolethalus Syndrome 1 (HLS1)	Unknown	0.0	0.2
IBA57	NM_001010867.4	Multiple Mitochondrial Dysfunctions Syndrome 3 (MMDS3)	Unknown	0.0	-0.6
IDH2	NM_002168.4	D-2-Hydroxyglutaric Aciduria 2	Unknown	0.9	1.3
IDS	NM_000202.8	Mucopolysaccharidosis II, 309900 (3) telomeric IDS2 source of inversion in IDS	Yes	1.0	1.6
IER3IP1	NM_016097.5	Microcephaly, Epilepsy, And Diabetes Syndrome (MEDS); Microcephaly, Epilepsy, And Diabetes Syndrome (MEDS)	Unknown	0.0	-0.4
IFIH1†	NM_022168.4	Diabetes Mellitus, Insulin-Dependent, 19 (IDDM19)	Unknown	0.0	-0.8
IFT140	NM_014714.4	Mainzer-Saldino Syndrome (MZSDS)	Unknown	0.0	-0.8
IKBKG	NM_001099857.5	Incontinentia pigmenti, 308300	Unknown	0.1	0.0
INPP5E	NM_019892.6	Intellectual Developmental Disorder, Truncal Obesity, Retinal Dystrophy, And Micropenis; Joubert Syndrome 1 (JBTS1)	No	0.0	0.5
INS	NM_000207.3	Maturity-Onset Diabetes Of The Young, Type 10 (MODY10); Diabetes Mellitus, Permanent Neonatal (PNDM); Insulin (INS); Diabetes Mellitus, Insulin-Dependent, 2	Unknown	0.3	0.9
INSR	NM_000208.4	Diabetes Mellitus, Insulin-Resistant, With Acanthosis Nigricans; Hyperinsulinemic Hypoglycemia, Familial, 5 (HHF5); Pineal Hyperplasia, Insulin-Resistant Diabetes Mellitus, And Somatic; Donohue Syndrome; Diabetes Mellitus, Noninsulin-Dependent (NIDDM)	Unknown	0.0	3.8
IQCB1	NM_001023570.4	Senior-Loken Syndrome 5 (SLSN5)	Unknown	0.0	0.3
IQSEC1	NM_001134382.3	Intellectual developmental disorder with short stature and behavioral abnormalities, 618687 (3)	Unknown	1.0	2.3
IQSEC2	NM_001111125.3	Intellectual Developmental Disorder, X-Linked 1 (MRX1); Intellectual Developmental Disorder, X-Linked 1 (MRX1)	Yes	1.0	5.2
IRF2BPL	NM_024496.4	Neurodevelopmental disorder with regression, abnormal movements, loss of speech, and seizures, 618088 (3)	Unknown	0.8	0.7
ITGB6	NM_000888.5	Amelogenesis imperfecta, type IH, 616221 (3)	Unknown	0.0	-0.7
ITPA	NM_033453.4	Inosine Triphosphatase Deficiency	Unknown	0.0	0.1

© 2023 Koh HY et al. *JAMA Network Open.*

IVD	NM_002225.5	Isovaleric Acidemia (IVA)	Unknown	0.0	0.3
JAM3	NM_032801.5	Hemorrhagic Destruction of The Brain, Subependymal Calcification,	No	0.0	-0.9
KANSL1†	NM_015443.4	Koolen-De Vries syndrome, 610443 (3)	Yes	1.0	1.2
KARS	NM_005548.3	Charcot-Marie-Tooth Disease, Recessive Intermediate B (CMTRIB)	No	0.0	-0.7
KAT6A†	NM_006766.5	Arboleda-Tham syndrome, 616268 (3)	Yes	1.0	2.1
KAT6B†	NM_012330.4	Genitopatellar Syndrome (GTPTS); Ohdo Syndrome, Sbbys Variant (SBBYSS)	Yes	1.0	2.9
KAT8	NM_032188.3	Li-Ghorgani-Weisz-Hubshman syndrome, 618974 (3)	Unknown	0.2	3.2
KATNB1	NM_005886.3	Lissencephaly 6, with microcephaly, 616212 (3)	Unknown	0.0	1.6
KCNA1†	NM_000217.3	Episodic Ataxia, Type 1 (EA1)	Unknown	0.1	3.3
KCNA2†	NM_004974.4	Epileptic encephalopathy, early infantile, 32, 616366 (3)	Unknown	0.9	3.8
KCNB1	NM_004975.4	Epileptic encephalopathy, early infantile, 26, 616056 (3)	Unknown	1.0	4.3
KCNC1†	NM_001112741.2	Epilepsy, progressive myoclonic 7, 616187 (3)	Unknown	1.0	4.5
KCNC3†	NM_004977.3	Spinocerebellar Ataxia 13 (SCA13)	Unknown	0.2	3.0
KCND2	NM_012281.3	epilepsy;autism	Unknown	0.0	3.5
KCNE2	NM_172201.2	Long QT syndrome 6, 613693 (3); Atrial fibrillation, familial, 4, 611493 (3)	Unknown	0.0	0.1
KCNH1	NM_172362.3	Temple-Baraitser syndrome, 611816 (3); Zimmermann-Laband syndrome 1, 135500 (3)	Unknown	0.5	3.8
KCNH2†	NM_000238.4	Long Qt Syndrome 2 (LQT2); Short Qt Syndrome 1 (SQT1)	Yes	1.0	3.4
KCNH5	NM_139318.5	epilepsy	Unknown	0.0	2.5
KCNJ1	NM_153766.3	Bartter Syndrome, Antenatal, Type 2	No	0.0	-0.3
KCNJ10	NM_002241.5	Seizures, Sensorineural Deafness, Ataxia, Intellectual Developmental Disorder, And	Unknown	0.2	1.5
KCNJ11	NM_000525.4	Diabetes Mellitus, Transient Neonatal, 3; Diabetes Mellitus, Permanent Neonatal (PNDM); Hyperinsulinemic Hypoglycemia, Familial, 2 (HHF2)	Unknown	0.0	1.6
KCNJ13	NM_002242.4	Leber Congenital Amaurosis 16 (LCA16); Vitreoretinal Degeneration, Snowflake Type (SVD)	Unknown	0.0	1.9
KCNJ6	NM_002240.5	Keppen-Lubinsky syndrome, 614098 (3)	Unknown	0.5	3.7
KCNK4	NM_033310.3	Facial dysmorphism, hypertrichosis, epilepsy, intellectual/developmental delay, and gingival overgrowth syndrome, 618381 (3)	Unknown	0.0	0.8
KCNMA1	NM_001161352.2	Generalized Epilepsy and Paroxysmal Dyskinesia (GEPD)	Unknown	1.0	5.1
KCNN3†	NM_002249.6	Zimmermann-Laband syndrome 3, 618658 (3)	Unknown	1.0	3.3
KCNQ1	NM_000218.3	Long QT syndrome 1, 192500 (3); Jervell and Lange-Nielsen syndrome, 220400 (3); Atrial fibrillation, familial, 3, 607554 (3); Short QT syndrome 2, 609621 (3); Long QT syndrome 1, acquired, susceptibility to, 192500 (3)	Unknown	0.0	1.8
KCNQ2†	NM_172107.4	Epileptic Encephalopathy, Early Infantile, 7 (EIEE7); Seizures, Benign Familial Neonatal, 1 (BFNS1)	Yes	1.0	4.0
KCNQ3†	NM_004519.4	Seizures, benign neonatal, 2, 121201 (3)	Unknown	0.8	2.1
KCNQ5†	NM_019842.4	Intellectual Developmental Disorder, autosomal dominant 46, 617601 (3)	Unknown	1.0	3.3
KCNT1†	NM_020822.3	Epileptic encephalopathy, early infantile, 14, 614959 (3); Epilepsy, nocturnal frontal lobe, 5, 615005 (3)	Unknown	0.0	2.9
KCNT2†	NM_198503.5	Epileptic encephalopathy, early infantile, 57, 617771 (3) mutation identified in 1 EIEE57 patient	Unknown	0.0	3.6
KCTD3	NM_016121.5	No OMIM number;Epileptic encephalopathy	Unknown	0.1	2.8
KCTD7	NM_153033.5	Epilepsy, progressive myoclonic 3, with or without intracellular inclusions, 611726 (3)	Unknown	0.0	1.5
KDM4B†	NM_015015.3	Intellectual developmental disorder, autosomal dominant 65	Unknown	1.0	3.5
KDM5B	NM_006618.5	Intellectual Developmental Disorder, autosomal recessive 65, 618109 (3)	Uncertain	0.0	1.8
KDM5C	NM_004187.5	Intellectual Developmental Disorder, X-Linked, Syndromic, Claes-Jensen Type (MRXSCJ); Intellectual Developmental Disorder, X-Linked, Syndromic, Claes-Jensen Type (MRXSCJ)	Yes	1.0	5.1
KDM6A	NM_001291415.2	Kabuki syndrome 2, 300867 (3) UTY also in mouse and man; escapes inactivation	Yes	1.0	2.9
KDM6B†	NM_001348716.2	Neurodevelopmental disorder with coarse facies and mild distal skeletal abnormalities, 618505 (3)	Uncertain	1.0	1.3
KIAA0226	NM_014687.4	Spinocerebellar ataxia, autosomal recessive 15, 615705 (3)	Unknown	0.0	1.5
KIAA0556	NM_015202.5	Joubert syndrome 26, 616784 (3)	Unknown	0.0	-0.1

© 2023 Koh HY et al. *JAMA Network Open.*

KIAA0586	NM_001329943.3	Joubert syndrome 23, 616490 (3); Short-rib thoracic dysplasia 14 with polydactyly, 616546 (3)	Unknown	0.0	0.4
KIAA1109	NM_001384125.1	Alkuraya-Kucinskas syndrome, 617822 (3)	Unknown	0.0	6.0
KIF11†	NM_004523.4	Microcephaly with or without Chorioretinopathy, Lymphedema, or Impaired Intellectual Development	Yes	1.0	3.3
KIF1A	NM_001244008.2	Intellectual Developmental Disorder, Autosomal Dominant 9 (MRD9); Neuropathy, Hereditary Sensory, Type Iic (HSN2C); Spastic Paraplegia 30, Autosomal Recessive (SPG30)	Unknown	1.0	5.2
KIF2A	NM_001098511.3	Cortical dysplasia, complex, with other brain malformations 3, 615411 (3)	Unknown	1.0	4.0
KIF4A	NM_012310.5	Intellectual Developmental Disorder, X-linked 100, 300923 (3) mutation identified in 1 MRX100 family	Unknown	1.0	2.6
KIF5A†	NM_004984.4	Spastic Paraplegia 10, Autosomal Dominant (SPG10)	Unknown	1.0	3.6
KIF5C	NM_004522.3	Cortical dysplasia, complex, with other brain malformations 2, 615282 (3)	Unknown	1.0	4.1
KIF7	NM_198525.3	Hydrolethalus Syndrome 2 (HLS2); Bardet-Biedl Syndrome (BBS); Acrocallosal Syndrome (ACLS); Pallister-Hall Syndrome (PHS)	No	0.0	-0.7
KIFBP	NM_015634.4	Goldberg-Shprintzen megacolon syndrome	Unknown	N/A	N/A
KIRREL3†	NM_032531.4	Intellectual Developmental Disorder, Autosomal Dominant 4 (MRD4)	Unknown	1.0	2.4
KLHL15	NM_030624.3	Intellectual Developmental Disorder, X-linked 103, 300982 (3)	Unknown	1.0	3.7
KLLN	NM_001126049.2	Cowden Syndrome 4 (CWS4)	Unknown	0.0	-1.0
KMT2A†	NM_001197104.2	Hairy Elbows, Short Stature, Facial Dysmorphism, And Developmental; Myeloid/Lymphoid or Mixed Lineage Leukemia Gene (MLL); Hairy Elbows, Short Stature, Facial Dysmorphism, And Developmental; Myeloid/Lymphoid Or Mixed Lineage Leukemia Gene (MLL)	Yes	1.0	6.2
KMT2D†	NM_003482.4	Kabuki Syndrome 1 (KABUK1)	Yes	1.0	3.7
KMT2E†	NM_182931.3	O'Donnell-Luria-Rodan syndrome, 618512 (3)	Unknown	1.0	1.4
KMT5B	NM_017635.5	Intellectual Developmental Disorder, autosomal dominant 51	Unknown	N/A	N/A
KPTN	NM_007059.4	Intellectual Developmental Disorder, autosomal recessive 41, 615637 (3)	Unknown	0.0	1.4
KRAS	NM_004985.5	Lung cancer, somatic, 211980 (3); Bladder cancer, somatic, 109800 (3); Pancreatic carcinoma, somatic, 260350 (3); Gastric cancer, somatic, 137215 (3); Leukemia, acute myeloid, somatic, 601626 (3); Noonan syndrome 3, 609942 (3); Cardiofaciocutaneous syndrome 2, 615278 (3); Breast cancer, somatic, 114480 (3); Schimmelpenning-Feuerstein-Mims syndrome, somatic mosaic, 163200 (3); RAS-associated autoimmune leukoproliferative disorder, 614470 (3); Arteriovenous malformation of the brain, somatic, 108010 (3); Oculoectodermal syndrome, somatic, 600268 (3) pseudogene KRAS1P on 6p12-p11	Unknown	0.0	2.3
KRIT1	NM_194454.3	Cerebral Cavernous Malformations (CCM)	Unknown	0.0	1.4
L1CAM	NM_001278116.2	Hydrocephalus Due to Congenital Stenosis Of Aqueduct Of Sylvius (HSAS); Corpus Callosum, Partial Agenesis Of, X-Linked; Masa Syndrome; Hydrocephalus Due To Congenital Stenosis Of Aqueduct Of Sylvius (HSAS); Corpus Callosum, Partial Agenesis Of, X-Linked; Masa Syndrome	Yes	1.0	2.8
L2HGDH	NM_024884.3	L-2-Hydroxyglutaric Aciduria	Unknown	0.0	0.4
LAMA2	NM_000426.4	Muscular Dystrophy, Congenital Merosin-Deficient, 1A (MDC1A)	No	0.0	0.0
LAMB1	NM_002291.3	Lissencephaly 5, 615191 (3)	Unknown	0.0	0.5
LAMC3	NM_006059.4	Cortical Malformations, Occipital (OCCM)	Unknown	0.0	-0.4
LARGE1	NM_133642.5	Muscular dystrophy-dystroglycanopathy (congenital with brain and eye anomalies), type A, 6	No	N/A	N/A
LARS	NM_020117.11	Infantile liver failure syndrome 1, 615438 (3) mutation identified in 1 family	Unknown	0.0	1.5
LARS2	NM_015340.4	Perrault Syndrome 4 (PRLTS4)	Unknown	0.0	1.3
LBR	NM_002296.4	Reynolds Syndrome; Hydrops-Ectopic Calcification-Moth-Eaten Skeletal Dysplasia; Pelger-Huet Anomaly (PHA)	No	0.2	0.3
LGI1†	NM_005097.4	Epilepsy, Familial Temporal Lobe, 1 (ETL1)	Unknown	1.0	2.8
LIAS	NM_006859.4	Pyruvate Dehydrogenase Lipoic Acid Synthetase Deficiency (PDHLD)	Unknown	0.0	1.3
LIG4	NM_206937.2	Lig4 Syndrome; Severe Combined Immunodeficiency with Sensitivity to Ionizing Radiation	No	0.0	0.2
LIPT1	NM_145199.3	Lipoyltransferase 1 deficiency, 616299 (3)	Unknown	0.0	0.3
LIPT2	NM_001144869.3	Encephalopathy, neonatal severe, with lactic acidosis and brain abnormalities, 617668 (3)	Unknown	0.6	-0.1

© 2023 Koh HY et al. *JAMA Network Open.*

LMBRD1	NM_018368.4	Methylmalonic Aciduria and Homocystinuria, Cblf Type	Unknown	0.0	0.3
LMNB2	NM_032737.4	Lipodystrophy, Partial, Acquired, Susceptibility To (APLD)	Unknown	1.0	0.7
LNPK	NM_030650.3	Neurodevelopmental disorder with epilepsy and hypoplasia of the corpus callosum	Unknown	N/A	N/A
LONP1	NM_004793.4	CODAS syndrome, 600373 (3)	Unknown	1.0	0.4
LRAT	NM_004744.5	Leber Congenital Amaurosis 14 (LCA14)	Unknown	0.0	0.0
LRP2	NM_004525.3	Donnai-Barrow Syndrome	No	1.0	2.1
LRPPRC	NM_133259.4	Leigh Syndrome, French Canadian Type (LSFC)	No	0.0	-1.9
LSS	NM_002340.6	Cataract 44, 616509 (3); Hypotrichosis 14, 618275 (3); Alopecia-Intellectual Developmental Disorder syndrome 4, 618840 (3)	Unknown	0.0	0.7
LYST	NM_000081.4	Chediak-Higashi Syndrome (CHS)	No	0.0	1.4
MACF1†	NM_001394062.1	Lissencephaly 9 with complex brainstem malformation, 618325 (3)	Unknown	1.0	3.4
MAF†	NM_005360.5	Cataract 21, Multiple Types (CTRCT21)	Unknown	0.7	1.2
MAFB	NM_005461.5	Multicentric Carpotarsal Osteolysis Syndrome (MCTO)	Unknown	0.9	2.2
MAGEL2	NM_019066.5	Schaaf-Yang syndrome, 615547 (3)	Uncertain	1.0	-0.6
MAN1B1	NM_016219.5	Intellectual Developmental Disorder, Autosomal Recessive 15 (MRT15)	Unknown	0.0	-0.7
MANBA	NM_005908.4	Mannosidosis, Beta A, Lysosomal (MANSB)	Unknown	0.0	0.0
MAP2K1	NM_002755.4	Cardiofaciocutaneous Syndrome 3 (CFC3)	Unknown	0.9	3.1
MAPK10†	NM_138982.4	Epileptic Encephalopathy;EPILEPTIC ENCEPHALOPATHY LENNOX-GASTAUT TYPE	Unknown	0.3	3.0
MAPT	NM_001377265.1	Supranuclear Palsy, Progressive, 1 (PSNP1); Frontotemporal Dementia (FTD); Parkinson-Dementia Syndrome; Pick Disease of Brain	Unknown	0.0	1.5
MAST1†	NM_014975.3	Mega-corpus-callosum syndrome with cerebellar hypoplasia and cortical malformations, 618273 (3)	Unknown	1.0	5.8
MATN4	NM_001393530.1	Global developmental delay, holoprosencephaly, microcephaly, lumbosacral myelomeningocele, epilepsy, proptosis, and diabetes insipidus	Unknown	0.0	1.1
MBD5†	NM_001378120.1	Intellectual Developmental Disorder, Autosomal Dominant 1 (MRD1)	Yes	1.0	0.9
MBOAT7	NM_024298.5	Intellectual Developmental Disorder, autosomal recessive 57, 617188 (3)	Unknown	0.1	0.4
MBTPS2	NM_015884.4	Keratosis Follicularis Spinulosa Decalvans, X-Linked (KFSDX); Ifap Syndrome with or without Bresheck Syndrome	Unknown	1.0	2.1
MC2R	NM_000529.2	Glucocorticoid Deficiency 1 (GCCD1)	Unknown	0.0	0.0
MCCC1	NM_020166.5	3-Methylcrotonyl-CoA carboxylase 1 deficiency, 210200 (3)	No	0.0	0.5
MCCC2	NM_022132.5	3-Methylcrotonyl-Coa Carboxylase 2 Deficiency; 3-Methylcrotonyl-Coa Carboxylase 2 Deficiency	Unknown	0.0	0.3
MCPH1	NM_024596.5	Microcephaly 1, Primary, Autosomal Recessive (MCPH1)	Unknown	0.0	-4.8
MDH1	NM_005917.4	Epileptic encephalopathy, early infantile, 88, 618959 (3) proximal to APOB; mutation identified in 1 EIEE88 family	Unknown	0.9	1.2
MDH2	NM_005918.4	Epileptic encephalopathy, early infantile, 51, 617339 (3)	Unknown	0.0	0.0
MECP2	NM_001110792.2	Rett syndrome, 312750 (3); Intellectual Developmental Disorder, X-linked, syndromic 13, 300055 (3); Rett syndrome, preserved speech variant, 312750 (3); Encephalopathy, neonatal severe, 300673 (3); Autism susceptibility, X-linked 3, 300496 (3); Intellectual Developmental Disorder, X-linked syndromic, Lubs type, 300260 (3); Rett syndrome, atypical, 312750 (3) 70kb centromeric of RCP/GCP	Unknown	0.9	-1.2
MED12	NM_005120.3	Lujan-Fryns Syndrome; Opitz-Kaveggia Syndrome (OKS); Ohdo Syndrome, X-Linked (OHDOX)	Unknown	1.0	6.6
MED13L†	NM_015335.5	Transposition of the Great Arteries, Dextro-Looped 1 (DTGA1)	Yes	1.0	3.7
MED17	NM_004268.5	Microcephaly, Postnatal Progressive, With Seizures and Brain Atrophy	Unknown	0.0	0.9
MED23	NM_004830.4	Intellectual Developmental Disorder, autosomal recessive 18, 614249 (3)	Unknown	0.0	4.7
MED25	NM_030973.4	Charcot-Marie-Tooth Disease, Axonal, Type 2B2 (CMT2B2)	Unknown	0.0	1.3
MEF2C†	NM_002397.5	Intellectual Developmental Disorder, Autosomal Dominant 20 (MRD20)	Yes	0.0	4.0
MEFV	NM_000243.3	Familial Mediterranean Fever (FMF); Familial Mediterranean Fever, Autosomal Dominant	Unknown	0.0	-1.7
MEGF10	NM_001256545.2	Myopathy, Areflexia, Respiratory Distress, And Dysphagia, Early-Onset	Unknown	0.4	1.0
MEIS2†	NM_170675.5	Cleft palate, cardiac defects, and Intellectual Developmental Disorder, 600987 (3)	Unknown	1.0	2.5
METTL23	NM_001080510.5	Intellectual Developmental Disorder, autosomal recessive 44, 615942 (3)	No	0.0	-1.0
MFF	NM_001277062.2	Encephalopathy due to defective mitochondrial and peroxisomal fission 2	Unknown	0.1	0.4

© 2023 Koh HY et al. *JAMA Network Open.*

MFSD2A	NM_032793.5	Neurodevelopmental disorder with progressive microcephaly, spasticity, and brain imaging abnormalities, 616486 (3)	Unknown	0.0	2.0
MFSD8	NM_001371596.2	Ceroid Lipofuscinosis, Neuronal, 7 (CLN7)	No	0.0	0.1
MGAT2	NM_002408.4	Congenital Disorder of Glycosylation, Type Iia (CDG2A)	Unknown	0.0	0.5
MGP	NM_000900.5	Keutel Syndrome	Unknown	0.0	0.1
MID2	NM_012216.4	Intellectual Developmental Disorder, X-linked 101, 300928 (3) mutation identified in 1 MRX101 family	Unknown	0.2	1.6
MKRN3	NM_005664.4	Precocious Puberty, Central, 2 (CPPB2)	Unknown	0.3	-0.4
MKS1	NM_017777.4	Meckel Syndrome, Type 1 (MKS1); Bardet-Biedl Syndrome (BBS)	No	0.0	0.5
MLC1	NM_015166.4	Megalencephalic Leukoencephalopathy with Subcortical Cysts 1 (MLC1)	Unknown	0.0	0.2
MLYCD	NM_012213.3	Malonyl-Coa Decarboxylase Deficiency	No	0.0	-1.8
MMAA	NM_172250.3	Methylmalonic Aciduria, Cbla Type	No	0.0	0.3
MMACHC	NM_015506.3	Homocysteinemia; Methylmalonic Aciduria and Homocystinuria, Cblc Type	Unknown	0.0	-1.1
MMADHC	NM_015702.3	Methylmalonic Aciduria and Homocystinuria, Cbld Type	No	0.0	-0.4
MN1	NM_002430.3	Meningioma, Familial, Susceptibility To	Unknown	1.0	2.2
MOCS1	NM_001358530.2	Molybdenum Cofactor Deficiency, Complementation Group A (MOCODA)	Unknown	0.0	-0.6
MOCS2	NM_004531.5	Molybdenum Cofactor Deficiency, Complementation Group A (MOCODA)	No	0.0	-0.1
MOGS	NM_006302.3	Congenital Disorder of Glycosylation, Type Iib (CDG2B)	Unknown	0.0	0.7
MPC1	NM_016098.4	Mitochondrial Pyruvate Carrier Deficiency (MPYCD)	Unknown	0.0	1.1
MPDU1	NM_004870.4	Congenital Disorder of Glycosylation, Type If (CDG1F)	Unknown	0.0	0.4
MPDZ	NM_001378778.1	Hydrocephalus, Nonsyndromic, Autosomal Recessive 2 (HYC2)	No	0.0	-3.0
MRAP	NM_001379228.1	Glucocorticoid Deficiency 2 (GCCD2)	Unknown	0.0	-0.1
MRPS22	NM_020191.4	Combined Oxidative Phosphorylation Deficiency 5 (COXPD5)	Unknown	0.0	0.1
MSX2	NM_002449.5	Craniosynostosis 2 (CRS2); Parietal Foramina with Cleidocranial Dysplasia (PFMCCD); Parietal Foramina (PFM)	Unknown	0.3	0.5
MTFMT	NM_139242.4	Combined Oxidative Phosphorylation Deficiency 15 (COXPD15); Leigh Syndrome (LS)	No	0.0	-0.5
MTHFR	NM_005957.5	Homocysteinemia; Neural Tube Defects, Folate-Sensitive; Stroke, Ischemic; Homocystinuria Due to Deficiency of N(5,10)-Methylenetetrahydrofolate	Unknown	0.0	0.9
MTHFS	NM_006441.4	Neurodevelopmental disorder with microcephaly, epilepsy, and hypomyelination, 618367 (3)	Unknown	0.0	0.6
MTM1	NM_000252.3	Myopathy, Centronuclear, X-Linked (CNMX); Myopathy, Centronuclear, X-Linked (CNMX)	Yes	1.0	2.4
MTO1	NM_012123.4	Combined Oxidative Phosphorylation Deficiency 10 (COXPD10)	No	0.0	0.6
MTOR	NM_004958.4	Smith-Kingsmore syndrome, 616638 (3); Focal cortical dysplasia, type II, somatic, 607341 (3)	Unknown	1.0	7.0
MTR	NM_000254.3	Homocysteinemia; Neural Tube Defects, Folate-Sensitive; Homocystinuria-Megaloblastic Anemia, Cblg Complementation Type (HMAG)	No	0.0	2.0
MTRR	NM_002454.3	Neural Tube Defects, Folate-Sensitive; Homocystinuria-Megaloblastic Anemia, Cble Complementation Type (HMAE)	No	0.0	-0.7
MVK	NM_000431.4	Mevalonic Aciduria (MEVA); Hyper-Igd Syndrome (HIDS)	Unknown	0.2	0.9
MYH3†	NM_002470.4	Arthrogryposis, Distal, Type 2B (DA2B); Arthrogryposis, Distal, Type 2A (DA2A)	Unknown	0.0	1.7
MYO5A	NM_001382347.1	Griscelli Syndrome, Type 3 (GS3); Elejalde Disease; Griscelli Syndrome, Type 1 (GS1)	No	0.9	3.1
MYT1L†	NM_001303052.2	Intellectual Developmental Disorder, autosomal dominant 39, 616521 (3)	Yes	1.0	4.8
NAA10	NM_003491.4	Ogden Syndrome (OGDNS); Ogden Syndrome (OGDNS)	Unknown	0.8	2.4
NACC1†	NM_052876.4	Neurodevelopmental disorder with epilepsy, cataracts, feeding difficulties, and delayed brain myelination, 617393 (3)	Unknown	1.0	4.2
NADK2	NM_001085411.3	2,4-dienoyl-CoA reductase deficiency, 616034 (3)	Unknown	0.1	2.0
NAGA	NM_000262.3	Kanzaki Disease; Schindler Disease, Type I	Unknown	0.0	0.2
NAGLU	NM_000263.4	Mucopolysaccharidosis Type Iiib	Unknown	0.0	1.0
NAGS	NM_153006.3	N-Acetylglutamate Synthase Deficiency	Unknown	0.0	1.3
NALCN	NM_052867.4	Hypotonia, infantile, with psychomotor retardation and characteristic facies 1, 615419 (3); Congenital contractures of the limbs and face, hypotonia, and developmental delay, 616266 (3)	Unknown	0.0	5.0

© 2023 Koh HY et al. *JAMA Network Open.*

NARS2	NM_024678.6	Combined oxidative phosphorylation deficiency 24, 616239 (3); Deafness, autosomal recessive 94, 618434 (3) mutation identified in 1 DFNB94 family	Unknown	0.0	-0.8
NAT8L	NM_178557.4	N-Acetylaspartate Deficiency (NACED)	No	0.9	2.1
NBEA†	NM_001385012.1	Neurodevelopmental disorder with or without early-onset generalized epilepsy	Yes	1.0	5.5
NDE1	NM_017668.3	Lissencephaly 4 (LIS4)	No	0.0	-1.1
NDN	NM_002487.3	Prader-Willi Syndrome (PWS)	Unknown	0.7	0.7
NDP	NM_000266.4	Norrie Disease (ND); Exudative Vitreoretinopathy 2, X-Linked (EVR2)	Yes	0.7	1.0
NDST1	NM_001543.5	Intellectual Developmental Disorder, autosomal recessive 46, 616116 (3)	Unknown	1.0	2.9
NDUFA1	NM_004541.4	Mitochondrial Complex I Deficiency	Unknown	0.6	1.0
NDUFA10	NM_004544.4	Mitochondrial complex I deficiency, nuclear type 22, 618243 (3)	No	0.0	-0.4
NDUFA11	NM_175614.5	Mitochondrial Complex I Deficiency	Unknown	0.0	0.5
NDUFA12	NM_018838.5	Leigh Syndrome (LS)	Unknown	0.0	0.0
NDUFA2	NM_002488.5	Mitochondrial complex I deficiency, nuclear type 13, 618235 (3)	Unknown	0.8	-0.2
NDUFA4	NM_002489.4	Mitochondrial complex IV deficiency, nuclear type 21	Unknown	0.0	0.0
NDUFA9	NM_005002.5	Mitochondrial complex I deficiency, nuclear type 26, 618247 (3)	Unknown	0.0	0.1
NDUFAF2	NM_174889.5	Mitochondrial Complex I Deficiency	No	0.0	-0.1
NDUFAF3	NM_199069.2	Mitochondrial complex I deficiency, nuclear type 18, 618240 (3)	Unknown	0.0	-0.5
NDUFAF4	NM_014165.4	Mitochondrial Complex I Deficiency	Unknown	0.6	0.1
NDUFAF5	NM_024120.5	Leigh Syndrome (LS); Mitochondrial Complex I Deficiency	No	0.0	0.2
NDUFAF6	NM_152416.4	Mitochondrial Complex I Deficiency	Unknown	0.0	0.6
NDUFB11	NM_001135998.3	Linear skin defects with multiple congenital anomalies 3, 300952 (3); Mitochondrial complex I deficiency, nuclear type 30, 301021 (3) mutation identified in 1 MC1DN30 patient	Unknown	0.8	0.5
NDUFS1	NM_005006.7	Mitochondrial Complex I Deficiency	Unknown	0.0	-0.1
NDUFS2	NM_001377299.1	Mitochondrial Complex I Deficiency	Unknown	0.0	1.5
NDUFS3	NM_004551.3	Mitochondrial complex I deficiency, nuclear type 8, 618230 (3)	Unknown	0.0	0.0
NDUFS4	NM_002495.4	Mitochondrial Complex I Deficiency	Unknown	0.0	-0.8
NDUFS6	NM_004553.6	Mitochondrial complex I deficiency, nuclear type 9, 618232 (3)	No	0.1	-0.2
NDUFS7	NM_024407.5	Leigh Syndrome (LS); Mitochondrial Complex I Deficiency	No	0.9	1.0
NDUFS8	NM_002496.4	Leigh Syndrome (LS)	No	0.2	0.7
NDUFV1	NM_007103.4	Leigh Syndrome (LS); Mitochondrial Complex I Deficiency	No	0.0	0.3
NDUFV2	NM_021074.5	Mitochondrial complex I deficiency, nuclear type 7, 618229 (3) pseudogene on 19q13.3-qter	No	0.0	-0.6
NECAP1	NM_015509.4	Epileptic encephalopathy, early infantile, 21, 615833 (3)	Unknown	0.1	1.4
NEDD4L†	NM_001144967.3	Periventricular nodular heterotopia 7, 617201 (3)	Unknown	1.0	3.7
NEK9	NM_033116.6	Lethal congenital contracture syndrome 10, 617022 (3); Arthrogryposis, Perthes disease, and upward gaze palsy, 614262 (3); Nevus comedonicus, somatic, 617025 (3) mutation identified in 1 APUG family	Unknown	0.0	2.5
NEU1	NM_000434.4	Neuraminidase Deficiency; Neuraminidase Deficiency; Neuraminidase Deficiency; Neuraminidase Deficiency; Neuraminidase Deficiency; Neuraminidase Deficiency; Neuraminidase Deficiency; Neuraminidase Deficiency	Unknown	0.0	1.4
NEUROD2†	NM_006160.4	Epileptic encephalopathy, early infantile, 72, 618374 (3)	Unknown	0.9	2.7
NEXMIF	NM_001008537.3	Intellectual Developmental Disorder, X-linked 98	Yes	N/A	N/A
NF1†	NM_001042492.3	Juvenile Myelomonocytic Leukemia (JMML); Neurofibromatosis-Noonan Syndrome (NFNS); Watson Syndrome; Neurofibromatosis, Familial Spinal; Neurofibromatosis, Type I (NF1); Colorectal Cancer (CRC)	Yes	0.9	6.5
NFIA	NM_001134673.4	Brain malformations with or without urinary tract defects, 613735 (3)	Yes	1.0	3.2
NFIX†	NM_001365902.3	Sotos Syndrome 2 (SOTOS2); Marshall-Smith Syndrome (MRSHSS)	Unknown	1.0	4.1
NGLY1	NM_018297.4	Congenital Disorder of Glycosylation, Type Iv (CDG1V)	No	0.0	-0.1
NHLRC1	NM_198586.3	Myoclonic Epilepsy of Lafora	Unknown	0.1	0.5
NID1	NM_002508.3	Hydrocephalus, focal epilepsy and hemiparesis	Unknown	0.0	0.5
NIN	NM_020921.4	Seckel Syndrome 7 (SCKL7)	Unknown	0.0	0.3

© 2023 Koh HY et al. *JAMA Network Open.*

NIPA1	NM_144599.5	Spastic Paraplegia 6, Autosomal Dominant (SPG6)	Unknown	0.0	1.9
NIPBL	NM_133433.4	Cornelia De Lange Syndrome 1 (CDLS1)	Yes	1.0	5.6
NLGN4X	NM_181332.3	Asperger Syndrome, X-Linked, Susceptibility To, 2 (ASPGX2); Autism, Susceptibility To, X-Linked 2 (AUTSX2)	Unknown	1.0	2.7
NNT	NM_182977.3	Glucocorticoid Deficiency 4 (GCCD4)	Unknown	1.0	2.1
NODAL	NM_018055.5	Heterotaxy, Visceral, 5, Autosomal (HTX5)	Uncertain	1.0	1.0
NOTCH3†	NM_000435.3	Myofibromatosis, Infantile, 2 (IMF2); Cerebral Arteriopathy, Autosomal Dominant, With Subcortical Infarcts	Unknown	0.4	3.5
NPC1	NM_000271.5	Niemann-Pick Disease, Type C1 (NPC1)	Unknown	0.0	1.1
NPC2	NM_006432.5	Niemann-Pick Disease, Type C2	Unknown	0.0	1.3
NPHP1	NM_001128178.3	Joubert Syndrome 4 (JBTS4); Senior-Loken Syndrome 1 (SLSN1); Nephronophthisis 1 (NPHP1)	No	0.0	0.2
NPRL2†	NM_006545.5	Epilepsy, familial focal, with variable foci 2, 617116 (3)	Unknown	0.4	1.6
NPRL3†	NM_001077350.3	Epilepsy, familial focal, with variable foci 3, 617118 (3)	Unknown	0.2	1.8
NR2F1	NM_005654.6	Bosch-Boonstra-Schaaf optic atrophy syndrome, 615722 (3)	Unknown	1.0	4.2
NRAS	NM_002524.5	Autoimmune Lymphoproliferative Syndrome, Type Iv (ALPS4); Noonan Syndrome 6 (NS6); Juvenile Myelomonocytic Leukemia (JMML)	Unknown	0.5	1.7
NRROS	NM_198565.3	Seizures, early-onset, with neurodegeneration and brain calcification, 618875 (3)	Unknown	0.3	0.9
NRXN1†	NM_001330078.2	Pitt-Hopkins-like syndrome 2, 614325 (3); Schizophrenia, susceptibility to, 17, 614332 (3)	Yes	1.0	2.6
NSD1†	NM_022455.5	Beckwith-Wiedemann Syndrome (BWS); Sotos Syndrome 1 (SOTOS1)	Yes	1.0	3.4
NSDHL	NM_015922.3	Congenital Hemidysplasia with Ichthyosiform Erythroderma And Limb; Ck Syndrome; Congenital Hemidysplasia with Ichthyosiform Erythroderma And Limb; Ck Syndrome	Yes	1.0	0.9
NSF	NM_006178.4	Seizures;EEG with burst suppression;Global developmental delay;Intellectual disability	Unknown	0.0	2.6
NSUN2	NM_017755.6	Intellectual Developmental Disorder, Autosomal Recessive 5 (MRT5)	Unknown	0.0	0.0
NTRK2†	NM_006180.6	Obesity, hyperphagia, and developmental delay, 613886 (3); Epileptic encephalopathy, early infantile, 58, 617830 (3)	Unknown	1.0	3.7
NUBPL	NM_025152.3	Mitochondrial Complex I Deficiency	Unknown	0.0	0.3
NUP214	NM_005085.4	Leukemia, acute myeloid, somatic, 601626 (3); Leukemia, T-cell acute lymphoblastic, somatic, 613065 (3); Encephalopathy, acute, infection-induced, susceptibility to, 9, 618426 (3) fused with DEK in AML; fused with ABL1 in T-ALL	Unknown	0.1	0.7
NUS1	NM_138459.5	Congenital disorder of glycosylation, type 1aa, 617082 (3); Intellectual Developmental Disorder, autosomal dominant 55, with seizures, 617831 (3) mutation identified in 1 CDG1AA family	Unknown	1.0	0.9
OCLN	NM_001205254.2	Band-Like Calcification with Simplified Gyration and Polymicrogyria; Band-Like Calcification With Simplified Gyration And Polymicrogyria	Unknown	0.0	0.3
OCRL	NM_000276.4	Lowe Oculocerebrorenal Syndrome (OCRL); Dent Disease 2	Yes	1.0	3.0
OFD1	NM_003611.3	Orofaciodigital syndrome I, 311200 (3); Simpson-Golabi-Behmel syndrome, type 2, 300209 (3); Joubert syndrome 10, 300804 (3); Retinitis pigmentosa 23, 300424 (3) mutation identified in 1 RP23 family	Yes	1.0	0.3
OPA1†	NM_130837.3	Optic Atrophy 1 (OPA1); Optic Atrophy with Or Without Deafness, Ophthalmoplegia, Myopathy,	Unknown	1.0	2.0
OPHN1	NM_002547.3	Intellectual Developmental Disorder, X-Linked, With Cerebellar Hypoplasia and Distinctive	Yes	1.0	2.6
OTC	NM_000531.6	Ornithine Transcarbamylase Deficiency, Hyperammonemia Due To	Yes	0.9	1.3
OTUD6B	NM_016023.5	Intellectual developmental disorder with dysmorphic facies, seizures, and distal limb anomalies, 617452 (3)	Unknown	0.0	0.3
OTX2†	NM_021728.4	Pituitary Hormone Deficiency, Combined, 6 (CPHD6); Microphthalmia, Syndromic 5 (MCOPS5)	Yes	0.9	1.0
OXR1	NM_001198533.2	Cerebellar hypoplasia/atrophy, epilepsy, and global developmental delay, 213000 (3)	Unknown	0.8	1.0
P4HTM	NM_177939.3	Hypotonia, hypoventilation, impaired intellectual development, dysautonomia, epilepsy, and eye abnormalities, 618493 (3)	Unknown	0.0	1.5
PACS1†	NM_018026.4	Intellectual Developmental Disorder, Autosomal Dominant 17 (MRD17)	Unknown	1.0	3.7
PACS2†	NM_001100913.3	Epileptic encephalopathy, early infantile, 66, 618067 (3)	Unknown	1.0	2.2
PAFAH1B1	NM_000430.4	Lissencephaly 1, 607432 (3); Subcortical laminar heterotopia, 607432 (3)	Unknown	1.0	3.5
PAH	NM_000277.3	Phenylketonuria (PKU)	No	0.0	-0.6

PAK1†	NM_002576.5	Intellectual developmental disorder with macrocephaly, seizures, and speech delay, 618158 (3)	Unknown	0.0	4.0
PAK3	NM_002578.5	Intellectual Developmental Disorder, X-Linked 30 (MRX30)	Unknown	1.0	3.5
PANK2	NM_001386393.1	Hypoprebetalipoproteinemia, Acanthocytosis, Retinitis Pigmentosa; Neurodegeneration With Brain Iron Accumulation 1 (NBIA1)	Unknown	0.0	0.2
PARS2	NM_152268.4	Epileptic encephalopathy, early infantile, 75, 618437 (3)	Unknown	0.0	0.3
PC	NM_001040716.2	Pyruvate Carboxylase Deficiency	Unknown	0.0	3.1
PCCA	NM_000282.4	Propionic Acidemia	No	0.0	0.2
PCCB	NM_000532.5	Propionic Acidemia	Unknown	0.0	-0.9
PCDH12	NM_016580.4	Diencephalic-mesencephalic junction dysplasia syndrome 1, 251280 (3)	Unknown	0.0	0.8
PCDH19	NM_001184880.2	Epileptic Encephalopathy, Early Infantile, 9 (EIEE9)	Yes	1.0	2.6
PCDHB4	NM_018938.4	Intellectual disability, microcephaly and epilepsy	Unknown	0.0	-0.3
PCK1	NM_002591.4	Phosphoenolpyruvate carboxykinase deficiency, cytosolic, 261680 (3) mutation identified in 1 PCKDC family	Unknown	0.0	0.0
PCLO	NM_033026.6	Pontocerebellar hypoplasia, type 3, 608027 (3) mutation identified in 1 PCHD3 family	Unknown	1.0	-1.5
PCNT	NM_006031.6	Microcephalic Osteodysplastic Primordial Dwarfism, Type Ii (MOPD2)	Unknown	0.0	-1.7
PCSK1	NM_000439.5	Body Mass Index Quantitative Trait Locus 12 (BMIQ12); Proprotein Convertase 1/3 Deficiency	Unknown	0.0	1.5
PCYT2	NM_002861.5	Spastic paraplegia 82, autosomal recessive, 618770 (3)	Unknown	0.0	1.7
PDCD10†	NM_007217.4	Cerebral Cavernous Malformations 3 (CCM3)	Unknown	1.0	1.4
PDE6D	NM_002601.4	Joubert syndrome 22, 615665 (3) mutation identified in 1 JBTS22 family	Unknown	0.0	1.6
PDGFB	NM_002608.4	Dermatofibrosarcoma Protuberans (DFSP)	Unknown	0.8	1.1
PDGFRB	NM_002609.4	Basal Ganglia Calcification, Idiopathic, 4 (IBGC4); Juvenile Myelomonocytic Leukemia (JMML); Leukemia, Acute Myeloid (AML); Myofibromatosis, Infantile, 1 (IMF1); Myeloproliferative Disorder, Chronic, With Eosinophilia	Unknown	0.9	1.8
PDHA1	NM_000284.4	Pyruvate Dehydrogenase E1-Alpha Deficiency (PDHAD); Leigh Syndrome, X-Linked	Yes	1.0	2.6
PDHB	NM_000925.4	Pyruvate Dehydrogenase E1-Beta Deficiency (PDHBD)	Unknown	0.2	1.6
PDHX	NM_003477.3	Pyruvate Dehydrogenase E3-Binding Protein Deficiency (PDHXD)	Unknown	0.0	-0.2
PDP1	NM_018444.4	Pyruvate Dehydrogenase Phosphatase Deficiency (PDHPD)	Unknown	0.5	1.6
PDSS2	NM_020381.4	Coenzyme Q10 deficiency, primary, 3, 614652 (3)	Unknown	0.0	0.5
PET100	NM_001171155.2	Mitochondrial complex IV deficiency, 220110 (3)	Unknown	0.0	0.2
PEX1	NM_000466.3	Peroxisome Biogenesis Disorder 1B (PBD1B); Peroxisome Biogenesis Disorder 1A (Zellweger) (PBD1A)	Unknown	0.0	1.1
PEX10	NM_002617.4	Peroxisome Biogenesis Disorder 6B (PBD6B); Peroxisome Biogenesis Disorder 6A (Zellweger) (PBD6A)	Unknown	0.0	-0.3
PEX11B	NM_003846.3	Peroxisome Biogenesis Disorder 14B (PEX14B); Peroxisome Biogenesis Disorder 14B (PEX14B)	No	0.0	-0.3
PEX12	NM_000286.3	Peroxisome Biogenesis Disorder 3A (Zellweger) (PBD3A); Peroxisome Biogenesis Disorder 3B (PBD3B)	Unknown	0.0	0.5
PEX13	NM_002618.4	Peroxisome Biogenesis Disorder 11B (PBD11B); Peroxisome Biogenesis Disorder 11A (Zellweger) (PBD11A)	Unknown	0.0	-0.3
PEX14	NM_004565.3	Peroxisome Biogenesis Disorder 13A (Zellweger) (PBD13A)	No	0.2	1.3
PEX16	NM_004813.4	Peroxisome Biogenesis Disorder 8B (PBD8B); Peroxisome Biogenesis Disorder 8A (Zellweger) (PBD8A)	No	0.0	0.5
PEX19	NM_002857.4	Peroxisome Biogenesis Disorder 12A (Zellweger) (PBD12A)	Unknown	0.0	-0.8
PEX2	NM_000318.3	Peroxisome Biogenesis Disorder 5B (PBD5B); Peroxisome Biogenesis Disorder 5A (Zellweger) (PBD5A)	Unknown	0.0	0.0
PEX26	NM_001127649.3	Peroxisome Biogenesis Disorder 7B (PBD7B); Peroxisome Biogenesis Disorder 7A (Zellweger) (PBD7A)	No	0.9	-0.4
PEX3	NM_003630.3	Peroxisome Biogenesis Disorder 10A (Zellweger) (PBD10A)	No	0.0	1.2
PEX5	NM_001351132.2	Peroxisome Biogenesis Disorder 2A (Zellweger) (PBD2A); Peroxisome Biogenesis Disorder 2B (PBD2B)	Unknown	0.0	0.7
PEX6	NM_000287.4	Peroxisome Biogenesis Disorder 4B (PBD4B); Peroxisome Biogenesis Disorder 4A (Zellweger) (PBD4A)	Unknown	0.0	1.4

© 2023 Koh HY et al. *JAMA Network Open.*

PEX7	NM_000288.4	Peroxisome Biogenesis Disorder 9B (PBD9B); Rhizomelic Chondrodysplasia Punctata, Type 1 (RCDP1)	Unknown	0.0	0.5
PGAP1	NM_024989.4	Intellectual Developmental Disorder, autosomal recessive 42, 615802 (3)	Unknown	0.0	1.3
PGAP2	NM_014489.4	Hyperphosphatasia With Intellectual Developmental Disorder Syndrome 3 (HPMRS3)	Unknown	0.9	0.9
PGAP3	NM_033419.5	Hyperphosphatasia with Intellectual Developmental Disorder syndrome 4, 615716 (3)	Unknown	0.0	0.7
PGK1	NM_000291.4	Phosphoglycerate Kinase 1 Deficiency; Phosphoglycerate Kinase 1 Deficiency	Yes	0.8	0.3
PHACTR1	NM_030948.6	Epileptic encephalopathy, early infantile, 70, 618298 (3)	Unknown	0.6	0.7
PHF21A†	NM_001352027.3	Intellectual developmental disorder with behavioral abnormalities and craniofacial dysmorphism with or without seizures, 618725 (3)	Unknown	1.0	2.9
PHF6	NM_001015877.2	Borjeson-Forssman-Lehmann Syndrome (BFLS)	Yes	1.0	2.3
PHGDH	NM_006623.4	Phosphoglycerate Dehydrogenase Deficiency	Unknown	0.0	0.2
PHOX2B	NM_003924.4	Neuroblastoma, Susceptibility To, 2 (NBLST2); Central Hypoventilation Syndrome, Congenital (CCHS)	Unknown	0.9	2.1
PI4KA	NM_058004.4	Polymicrogyria, perisylvian, with cerebellar hypoplasia and arthrogryposis, 616531 (3)	Unknown	0.0	3.5
PIEZO2	NM_001378183.1	Arthrogryposis, distal, type 5, 108145 (3); Arthrogryposis, distal, type 3, 114300 (3); Marden-Walker syndrome, 248700 (3); Arthrogryposis, distal, with impaired proprioception and touch, 617146 (3) mutation identified in 1 MWKS patient	Unknown	0.0	3.4
PIGA	NM_002641.4	Multiple Congenital Anomalies-Hypotonia-Seizures Syndrome 2 (MCAHS2); Paroxysmal Nocturnal Hemoglobinuria 1 (PNH1)	Unknown	1.0	2.2
PIGB	NM_004855.5	Epileptic encephalopathy, early infantile, 80, 618580 (3)	Unknown	0.0	0.6
PIGC	NM_153747.2	Glycosylphosphatidylinositol biosynthesis defect 16, 617816 (3)	Unknown	0.0	0.8
PIGG	NM_001127178.3	Intellectual Developmental Disorder, autosomal recessive 53, 616917 (3)	Unknown	0.0	-0.2
PIGH	NM_004569.5	Glycosylphosphatidylinositol biosynthesis defect 17, 618010 (3)	Unknown	0.2	0.9
PIGL	NM_004278.4	Coloboma, Congenital Heart Disease, Ichthyosiform Dermatosis, Mental	Unknown	0.0	0.0
PIGM	NM_145167.3	Glycosylphosphatidylinositol Deficiency	Unknown	0.0	-0.1
PIGN	NM_176787.5	Multiple congenital anomalies-hypotonia-seizures syndrome 1, 614080 (3)	Unknown	0.0	0.4
PIGO	NM_032634.4	Hyperphosphatasia With Intellectual Developmental Disorder Syndrome 2 (HPMRS2)	No	0.0	1.0
PIGP	NM_153682.3	Epileptic encephalopathy, early infantile, 55, 617599 (3)	Unknown	0.0	-0.6
PIGQ	NM_004204.5	Epileptic encephalopathy, early infantile, 77, 618548 (3)	Unknown	0.0	-0.1
PIGS	NM_033198.4	Glycosylphosphatidylinositol biosynthesis defect 18, 618143 (3)	Unknown	0.0	0.8
PIGT	NM_015937.6	Paroxysmal nocturnal hemoglobinuria 2, 615399 (3); Multiple congenital anomalies-hypotonia-seizures syndrome 3, 615398 (3) mutation identified in 1 PNH2 family	Unknown	0.0	0.3
PIGU	NM_080476.5	Neurodevelopmental disorder with brain anomalies, seizures, and scoliosis, 618590 (3)	Unknown	0.0	0.7
PIGV	NM_017837.4	Hyperphosphatasia With Intellectual Developmental Disorder Syndrome 1 (HPMRS1)	Unknown	0.0	0.4
PIGW	NM_001346754.2	Glycosylphosphatidylinositol biosynthesis defect 11	No	0.0	0.0
PIGY	NM_001042616.3	Hyperphosphatasia with Intellectual Developmental Disorder syndrome 6	Unknown	0.1	-0.3
PIK3CA	NM_006218.4	Cowden Syndrome 5 (CWS5); Congenital Lipomatous Overgrowth, Vascular Malformations, And Epidermal; Megalencephaly-Polymicrogyria-Polydactyly-Hydrocephalus Syndrome; Megalencephaly-Capillary Malformation-Polymicrogyria Syndrome (MCAP); Keratosis, Seborrheic; Ovarian Cancer; Hepatocellular Carcinoma; Colorectal Cancer (CRC); Breast Cancer	Unknown	1.0	5.6
PIK3R2	NM_005027.4	Megalencephaly-Polymicrogyria-Polydactyly-Hydrocephalus Syndrome; Megalencephaly-Polymicrogyria-Polydactyly-Hydrocephalus Syndrome	Unknown	0.0	2.3
PLA2G6	NM_003560.4	Parkinson Disease 14, Autosomal Recessive (PARK14); Neurodegeneration with Brain Iron Accumulation 2B (NBIA2B); Neurodegeneration With Brain Iron Accumulation 2A (NBIA2A)	Unknown	0.0	1.2
PLAA	NM_001031689.3	Neurodevelopmental disorder with progressive microcephaly, spasticity, and brain anomalies, 617527 (3)	Unknown	0.5	1.1
PLCB1	NM_015192.4	Epileptic Encephalopathy, Early Infantile, 12 (EIEE12)	No	1.0	3.8
PLK4	NM_014264.5	Microcephaly and chorioretinopathy, autosomal recessive, 2, 616171 (3)	Unknown	0.0	0.8
PLP1	NM_000533.5	Spastic Paraplegia 2, X-Linked (SPG2); Pelizaeus-Merzbacher Disease (PMD)	Yes	0.9	2.0
PLPBP	NM_007198.4	Epilepsy, Early-onset, Vitamin B6-dependent	Unknown	N/A	N/A
PMM2	NM_000303.3	Congenital Disorder of Glycosylation, Type Ia (CDG1A)	No	0.0	-1.4
PMPCB	NM_004279.3	Multiple mitochondrial dysfunctions syndrome 6, 617954 (3)	Unknown	0.0	-0.2

© 2023 Koh HY et al. *JAMA Network Open.*

PNKP	NM_007254.4	Microcephaly, seizures, and developmental delay, 613402 (3); Ataxia-oculomotor apraxia 4, 616267 (3); Charcot-Marie-Tooth disease, type 2B2, 605589 (3) mutation identified in 1 CMT2B2 family	Unknown	0.0	-1.3
PNPLA8	NM_001256007.3	Mitochondrial myopathy with lactic acidosis, 251950 (3) mutation identified in 1 MMLA family	Unknown	0.0	0.1
PNPO	NM_018129.4	Pyridoxamine 5-Prime-Phosphate Oxidase Deficiency	Unknown	0.0	0.8
POGZ†	NM_015100.4	White-Sutton syndrome, 616364 (3)	Yes	1.0	3.5
POLA1	NM_001330360.2	Pigmentary disorder, reticulate, with systemic manifestations, X-linked, 301220 (3); Van Esch-O'Driscoll syndrome, 301030 (3)	Unknown	1.0	2.0
POLG	NM_002693.3	Mitochondrial DNA Depletion Syndrome 4B (Mngie Type) (MTDPS4B); Sensory Ataxic Neuropathy, Dysarthria, And Ophthalmoparesis (SANDO); Progressive External Ophthalmoplegia with Mitochondrial DNA Deletions; Leigh Syndrome (LS); Mitochondrial DNA Depletion Syndrome 4A (Alpers Type) (MTDPS4A)	Unknown	0.0	-0.7
POLG2	NM_007215.4	Progressive External Ophthalmoplegia with Mitochondrial DNA Deletions; Progressive External Ophthalmoplegia with Mitochondrial DNA Deletions,	Unknown	0.0	-0.3
POLR2A	NM_000937.5	Neurodevelopmental disorder with hypotonia and variable intellectual and behavioral abnormalities	Unknown	1.0	7.1
POLR3A	NM_007055.4	Leukodystrophy, Hypomyelinating, 7, with or without Oligodontia And/Or	Unknown	0.0	2.3
POMGNT1	NM_017739.4	Muscular Dystrophy-Dystroglycanopathy (Limb-Girdle), Type C, 3 (MDDGC3); Muscular Dystrophy-Dystroglycanopathy (Congenital with Intellectual Developmental Disorder); Muscular Dystrophy-Dystroglycanopathy (Congenital with Brain And Eye	Unknown	0.0	0.9
POMGNT2	NM_032806.6	Muscular Dystrophy-Dystroglycanopathy (Congenital with Brain and Eye	Unknown	0.0	1.0
POMK	NM_032237.5	Muscular Dystrophy-Dystroglycanopathy (Congenital with Brain and Eye	Unknown	0.0	0.5
POMT1	NM_001077365.2	Muscular Dystrophy-Dystroglycanopathy (Congenital with Intellectual Developmental Disorder); Muscular Dystrophy-Dystroglycanopathy (Limb-Girdle), Type C, 1 (MDDGC1); Muscular Dystrophy-Dystroglycanopathy (Congenital With Brain And Eye	Unknown	0.0	0.6
POMT2	NM_013382.7	Muscular Dystrophy-Dystroglycanopathy (Limb-Girdle), Type C, 2 (MDDGC2); Muscular Dystrophy-Dystroglycanopathy (Congenital with Impaired Intellectual Development); Muscular Dystrophy-Dystroglycanopathy (Congenital With Brain And Eye	Unknown	0.0	0.1
POU1F1	NM_000306.4	Pituitary Hormone Deficiency, Combined, 1 (CPHD1)	Unknown	0.0	0.5
PPM1K	NM_152542.5	Maple Syrup Urine Disease, Mild Variant (MSUDMV)	Unknown	0.0	1.3
PPOX	NM_001122764.3	Porphyria Variegata	Unknown	0.0	1.0
PPP2CA†	NM_002715.4	Neurodevelopmental disorder and language delay with or without structural brain abnormalities, 618354 (3)	Unknown	1.0	4.2
PPP2R1A†	NM_014225.6	Intellectual Developmental Disorder, autosomal dominant 36, 616362 (3)	Unknown	1.0	4.5
PPP2R5D†	NM_006245.4	Intellectual Developmental Disorder, autosomal dominant 35, 616355 (3)	Unknown	1.0	3.6
PPP3CA†	NM_000944.5	Epileptic encephalopathy, infantile or early childhood, 1, 617711 (3); Arthrogryposis, cleft palate, craniosynostosis, and impaired intellectual development, 618265 (3)	Unknown	1.0	3.6
PPT1	NM_000310.4	Ceroid Lipofuscinosis, Neuronal, 1 (CLN1)	No	0.0	0.0
PQBP1	NM_001032382.2	Renpenning Syndrome 1 (RENS1); Renpenning Syndrome 1 (RENS1)	Yes	0.8	1.9
PRDM8	NM_001099403.2	Epilepsy, progressive myoclonic, 10, 616640 (3) mutation identified in 1 EPM10 family	Unknown	0.8	0.0
PREPL	NM_001171613.2	Hypotonia-Cystinuria Syndrome	No	0.0	-2.5
PRF1	NM_001083116.3	Hemophagocytic Lymphohistiocytosis, Familial, 2 (FHL2)	Unknown	0.0	0.0
PRICKLE1	NM_153026.3	Epilepsy, Progressive Myoclonic 1B (EPM1B); Neural Tube Defects	Unknown	1.0	1.8
PRICKLE2†	NM_198859.4	Epilepsy, Progressive Myoclonic 5 (EPM5)	Unknown	1.0	1.7
PRKDC	NM_006904.7	Immunodeficiency 26, with or without neurologic abnormalities, 615966 (3)	Unknown	1.0	2.8
PRMT7	NM_019023.5	Short stature, brachydactyly, intellectual developmental disability, and seizures, 617157 (3)	Unknown	0.0	0.7
PRODH	NM_016335.6	Schizophrenia 4 (SCZD4); Hyperprolinemia, Type I (HPI); Schizophrenia (SCZD)	Unknown	0.0	0.1
PROKR2	NM_144773.4	Hypogonadotropic Hypogonadism 3 with or without Anosmia (HH3)	Unknown	0.0	-0.1
PROP1	NM_006261.5	Pituitary Hormone Deficiency, Combined, 2 (CPHD2)	No	0.1	0.2
PROSC	NM_007198.4	Epilepsy, early-onset, vitamin B6-dependent, 617290 (3)	Unknown	0.0	1.0
PRRT2†	NM_145239.3	Seizures, Benign Familial Infantile, 2 (BFIS2); Convulsions, Familial Infantile, With Paroxysmal Choreoathetosis; Episodic Kinesigenic Dyskinesia 1 (EKD1)	Unknown	0.6	0.2
PRSS12	NM_003619.4	Intellectual Developmental Disorder, Autosomal Recessive 1 (MRT1)	Unknown	0.0	0.4

© 2023 Koh HY et al. *JAMA Network Open.*

PRUNE	NM_021222.3	Neurodevelopmental disorder with microcephaly, hypotonia, and variable brain anomalies, 617481 (3)	Unknown	0.0	1.1
PSAP	NM_002778.4	Krabbe Disease, Atypical, Due to Saposin A Deficiency; Combined Saposin Deficiency; Gaucher Disease, Atypical, Due To Saposin C Deficiency; Metachromatic Leukodystrophy Due To Saposin B Deficiency	Unknown	1.0	-0.5
PSAT1	NM_058179.4	Phosphoserine Aminotransferase Deficiency	Unknown	0.0	0.4
PSEN1†	NM_000021.4	Acne Inversa, Familial, 3 (ACNINV3); Cardiomyopathy, Dilated, 1U (CMD1U); Alzheimer Disease 3; Frontotemporal Dementia (FTD)	Unknown	1.0	2.2
PSMB8	NM_148919.4	Proteasome-associated autoinflammatory syndrome 1 and digenic forms, 256040 (3) between TAP1 and TAP2	Unknown	0.0	0.7
PSPH	NM_004577.4	Phosphoserine Phosphatase Deficiency (PSPHD)	No	0.0	0.8
PTCH1†	NM_000264.5	Holoprosencephaly 7 (HPE7); Basal Cell Carcinoma, Susceptibility To, 1 (BCC1); Basal Cell Nevus Syndrome (BCNS)	Yes	1.0	1.7
PTCHD1	NM_173495.3	Autism, susceptibility to, X-linked 4, 300830 (3)	Yes	1.0	2.0
PTEN	NM_000314.8	Glioma Susceptibility 2 (GLM2); Chromosome 10Q23 Deletion Syndrome; Endometrial Cancer; Macrocephaly/Autism Syndrome; Vacterl Association with Hydrocephalus; Squamous Cell Carcinoma, Head and Neck (HNSCC); Prostate Cancer; Cowden Syndrome 1 (CWS1); Bannayan-Riley-Ruvalcaba Syndrome (BRRS); Glioma Susceptibility 1 (GLM1)	Yes	0.3	3.5
PTF1A	NM_178161.3	Pancreatic and cerebellar agenesis, 609069 (3); Pancreatic agenesis 2, 615935 (3)	Unknown	0.2	0.5
PTH	NM_000315.4	Hypoparathyroidism, Familial Isolated (FIH)	Unknown	0.0	0.5
PTPN22	NM_015967.7	Systemic Lupus Erythematosus (SLE)	Unknown	0.0	0.9
PTPN23	NM_015466.4	Neurodevelopmental disorder and structural brain anomalies with or without seizures and spasticity, 618890 (3)	Unknown	0.0	1.3
PTS	NM_000317.3	Hyperphenylalaninemia, Bh4-Deficient, A (HPABH4A)	No	0.0	0.1
PUM1†	NM_001020658.2	Spinocerebellar ataxia 47, 617931 (3)	Unknown	1.0	4.4
PURA†	NM_005859.5	Intellectual Developmental Disorder, autosomal dominant 31, 616158 (3)	Yes	0.9	3.4
PUS3	NM_031307.4	Intellectual Developmental Disorder, autosomal recessive 55, 617051 (3)	Unknown	0.0	0.1
QARS	NM_005051.3	Microcephaly, progressive, seizures, and cerebral and cerebellar atrophy, 615760 (3)	Unknown	0.0	0.2
QDPR	NM_000320.3	Hyperphenylalaninemia, Bh4-Deficient, C (HPABH4C)	Unknown	0.0	0.3
RAB11B	NM_004218.4	Neurodevelopmental disorder with ataxic gait, absent speech, and decreased cortical white matter, 617807 (3)	Unknown	0.4	2.5
RAB18	NM_021252.5	Warburg Micro Syndrome 3 (WARBM3)	Unknown	0.9	1.4
RAB27A	NM_183235.3	Griscelli syndrome, type 2, 607624 (3)	Unknown	0.0	-0.5
RAB39B	NM_171998.4	Intellectual Developmental Disorder, X-Linked 72 (MRX72); Intellectual Developmental Disorder, X-Linked 72 (MRX72)	Yes	0.8	1.9
RAB3GAP1	NM_012233.3	Warburg Micro Syndrome 1 (WARBM1)	Unknown	0.0	1.2
RAB3GAP2	NM_012414.4	Warburg Micro Syndrome 2 (WARBM2); Martsolf Syndrome	No	1.0	0.9
RAD21	NM_006265.3	Cornelia De Lange Syndrome 4 (CDLS4)	Unknown	1.0	2.6
RAI1†	NM_030665.4	Smith-Magenis Syndrome (SMS)	Unknown	1.0	1.1
RALA	NM_005402.4	Global developmental delay;Intellectual disability;Seizures;Abnormality of nervous system morphology	Unknown	1.0	2.7
RALGAPA1	NM_001346249.2	Neurodevelopmental disorder with hypotonia, neonatal respiratory insufficiency, and thermodysregulation, 618797 (3) pseudogene on 9q31.1	Unknown	1.0	3.5
RANBP2	NM_006267.5	Encephalopathy, Acute, Infection-Induced, Susceptibility To, 3 (IIAE3)	Unknown	1.0	-0.8
RARS	NM_002887.4	Leukodystrophy, hypomyelinating, 9, 616140 (3)	Unknown	0.0	0.5
RARS2	NM_020320.5	Pontocerebellar Hypoplasia, Type 6 (PCH6)	No	0.0	-0.1
RBM10	NM_005676.5	Tarp Syndrome (TARPS); Tarp Syndrome (TARPS)	Unknown	1.0	4.5
RBPJ	NM_015874.6	Adams-Oliver Syndrome 3 (AOS3)	Unknown	1.0	3.6
RD3	NM_001164688.2	Leber Congenital Amaurosis 12 (LCA12)	Unknown	0.0	0.6
RDH12	NM_152443.3	Leber Congenital Amaurosis 13 (LCA13)	Unknown	0.0	-0.1
RECQL4	NM_004260.4	Rothmund-Thomson syndrome, type 2, , 268400 (3); Baller-Gerold syndrome, 218600 (3); RAPADILINO syndrome, 266280 (3)	Unknown	0.0	-4.2
RELN	NM_005045.4	Lissencephaly 2 (LIS2)	No	1.0	2.2

© 2023 Koh HY et al. *JAMA Network Open.*

RERE[†]	NM_001042681.2	Neurodevelopmental disorder with or without anomalies of the brain, eye, or heart, 616975 (3)	Unknown	1.0	2.0
RFT1	NM_052859.4	Congenital Disorder of Glycosylation, Type In (CDG1N)	Unknown	0.0	1.0
RHOBTB2	NM_015178.3	Epileptic encephalopathy, early infantile, 64, 618004 (3)	Unknown	0.0	2.7
RMND1	NM_017909.4	Combined oxidative phosphorylation deficiency 11, 614922 (3)	No	0.0	0.8
RNASEH2A	NM_006397.3	Aicardi-Goutieres syndrome 4, 610333 (3)	Unknown	0.0	0.2
RNASEH2B	NM_024570.4	Aicardi-Goutieres Syndrome 2 (AGS2)	Unknown	0.0	-0.2
RNASEH2C	NM_032193.4	Aicardi-Goutieres syndrome 3, 610329 (3)	Unknown	0.0	-0.9
RNASET2	NM_003730.6	Leukoencephalopathy, Cystic, Without Megalencephaly	Unknown	0.0	0.2
RNF113A	NM_006978.3	Trichothiodystrophy 5, nonphotosensitive, 300953 (3)	Unknown	0.9	1.8
RNF125	NM_017831.4	Tenorio syndrome, 616260 (3)	Unknown	0.0	0.8
RNF13	NM_183381.3	Epileptic encephalopathy, early infantile, 73, 618379 (3)	Unknown	0.0	0.9
RNU4ATAC	NR_023343.1	Microcephalic osteodysplastic primordial dwarfism, type I	Unknown	N/A	N/A
ROBO3	NM_022370.4	Gaze Palsy, Familial Horizontal, With Progressive Scoliosis (HGPPS)	No	0.0	0.6
ROGDI	NM_024589.3	Kohlschutter-Tonz Syndrome (KTZS)	No	0.0	-1.3
RORA	NM_134261.3	Intellectual developmental disorder with or without epilepsy or cerebellar ataxia, 618060 (3)	Unknown	0.4	2.6
RORB[†]	NM_006914.4	Epilepsy, idiopathic generalized, susceptibility to, 15, 618357 (3)	Unknown	1.0	3.1
RPGRIP1L	NM_015272.5	Joubert syndrome 7, 611560 (3); Meckel syndrome 5, 611561 (3); COACH syndrome, 216360 (3)	Unknown	0.0	-0.1
RPIA	NM_144563.3	Ribose 5-Phosphate Isomerase Deficiency	Unknown	0.0	1.0
RPL10	NM_006013.5	Autism, Susceptibility To, X-Linked 5 (AUTSX5); Autism, Susceptibility To, X-Linked 5 (AUTSX5)	Unknown	0.9	2.6
RPS6KA3	NM_004586.3	Coffin-Lowry syndrome, 303600 (3); Intellectual Developmental Disorder, X-linked 19, 300844 (3)	Yes	1.0	4.5
RRM2B	NM_015713.5	Progressive External Ophthalmoplegia with Mitochondrial DNA Deletions; Mitochondrial DNA Depletion Syndrome 8A (Encephalomyopathic Type with Renal Tubulopathy)	Unknown	0.0	1.2
RTN4IP1	NM_032730.5	Optic atrophy 10 with or without ataxia, Intellectual Developmental Disorder, and seizures, 616732 (3)	Unknown	0.0	0.7
RTTN	NM_173630.4	Polymicrogyria With Seizures (PMGYS)	Unknown	0.0	0.1
RUBCN	NM_014687.4	Spinocerebellar ataxia, autosomal recessive 15	Unknown	N/A	N/A
RYR2[†]	NM_001035.3	Ventricular Tachycardia, Catecholaminergic Polymorphic, 1, with or without Atrial Dysfunction or Dilated Cardiomyopathy; Arrhythmogenic Right Ventricular Dysplasia, Familial, 2 (ARVD2)	Unknown	1.0	5.8
RYR3	NM_001036.6	Epileptic encephalopathy	Unknown	0.7	1.8
SAMHD1	NM_015474.4	Chilblain Lupus 2 (CHBL2); Aicardi-Goutieres Syndrome 5 (AGS5)	Unknown	0.0	1.5
SASS6	NM_194292.3	Microcephaly 14, primary, autosomal recessive, 616402 (3) mutation identified in 1 MCPH14 family	Unknown	0.0	1.0
SATB2	NM_001172509.2	Cleft Palate, Isolated (CPI)	Yes	1.0	4.1
SC5D	NM_006918.5	Lathosterolosis	Unknown	0.0	0.7
SCAMP5	NM_138967.4	Global developmental delay;Intellectual disability;Seizures;Abnormality of nervous system morphology;Behavioral abnormality	Unknown	0.7	2.0
SCARB2	NM_005506.4	Epilepsy, Progressive Myoclonic 4, with or without Renal Failure	Unknown	0.0	1.3
SCN10A	NM_006514.4	Episodic pain syndrome, familial, 2, 615551 (3)	Unknown	0.0	-0.7
SCN11A	NM_001349253.2	Neuropathy, hereditary sensory and autonomic, type VII, 615548 (3); Episodic pain syndrome, familial, 3, 615552 (3)	Unknown	0.0	1.0
SCN1A[†]	NM_001165963.4	Migraine, Familial Hemiplegic, 3 (FHM3); Dravet Syndrome; Generalized Epilepsy with Febrile Seizures Plus, Type 2 (GEFSP2)	Yes	1.0	5.2
SCN1B[†]	NM_001037.5	Brugada Syndrome 5 (BRGDA5); Generalized Epilepsy with Febrile Seizures Plus, Type 1 (GEFSP1)	Unknown	0.1	1.2
SCN2A[†]	NM_001040142.2	Epileptic Encephalopathy, Early Infantile, 11 (EIEE11); Seizures, Benign Familial Infantile, 3 (BFIS3)	Yes	1.0	6.5
SCN2B	NM_004588.5	Atrial fibrillation, familial, 14, 615378 (3)	Unknown	0.0	0.3
SCN3A[†]	NM_006922.4	Epilepsy, familial focal, with variable foci 4, 617935 (3); Epileptic encephalopathy, early infantile, 62, 617938 (3)	Unknown	1.0	4.6

© 2023 Koh HY et al. *JAMA Network Open.*

SCN3B	NM_001040151.2	Brugada Syndrome 7 (BRGDA7)	Unknown	0.1	0.8
SCN4A	NM_000334.4	Myasthenic Syndrome, Congenital, Acetazolamide-Responsive; Hypokalemic Periodic Paralysis, Type 2 (HOKPP2); Myotonia, Potassium-Aggravated; Hyperkalemic Periodic Paralysis (HYPP); Paramyotonia Congenita Of Von Eulenburg (PMC)	Unknown	0.0	1.6
SCN4B	NM_174934.4	Long Qt Syndrome 10 (LQT10)	Unknown	0.0	-0.2
SCN5A	NM_000335.5	Atrial Fibrillation, Familial, 10 (ATFB10); Sick Sinus Syndrome 1, Autosomal Recessive (SSS1); Long Qt Syndrome 3 (LQT3); Ventricular Fibrillation During Myocardial Infarction, Susceptibility; Cardiomyopathy, Dilated, 1E (CMD1E); Brugada Syndrome 1 (BRGDA1); Sudden Infant Death Syndrome; Progressive Familial Heart Block, Type Ia (PFHB1A); Atrial Standstill	Unknown	0.9	2.8
SCN8A†	NM_001330260.2	Epileptic Encephalopathy, Early Infantile, 13 (EIEE13); Cognitive Impairment with or without Cerebellar Ataxia (CIAT)	Unknown	1.0	7.6
SCN9A	NM_001365536.1	Generalized Epilepsy with Febrile Seizures Plus, Type 7 (GEFSP7); Indifference to Pain, Congenital, Autosomal Recessive; Paroxysmal Extreme Pain Disorder; Erythermalgia, Primary	Unknown	0.0	1.1
SCO2	NM_005138.3	Myopia 6 (MYP6); Cardioencephalomyopathy, Fatal Infantile, Due to Cytochrome C Oxidase	Unknown	0.0	-1.6
SDHA	NM_004168.4	Paragangliomas 5 (PGL5); Cardiomyopathy, Dilated, 1Gg (CMD1GG); Leigh Syndrome (LS); Mitochondrial Complex Ii Deficiency	Unknown	0.0	0.7
SDHAF1	NM_001042631.3	Mitochondrial Complex Ii Deficiency	No	0.1	0.4
SEC23B	NM_006363.6	Anemia, Dyserythropoietic Congenital, Type Ii (CDAN2)	No	0.0	0.5
SEPSECS	NM_016955.4	Pontocerebellar Hypoplasia, Type 2D (PCH2D)	Unknown	0.0	0.0
SERAC1	NM_032861.4	3-Methylglutaconic Aciduria with Deafness, Encephalopathy, And Leigh-Like	Unknown	0.0	1.5
SERPINI1	NM_001122752.2	Encephalopathy, Familial, with Neuroserpin Inclusion Bodies (FENIB)	Unknown	0.0	0.4
SETBP1	NM_015559.3	Myelodysplastic Syndrome (MDS); Leukemia, Chronic Myeloid (CML); Juvenile Myelomonocytic Leukemia (JMML); Leukemia, Acute Myeloid (AML); Schinzel-Giedion Midface Retraction Syndrome	Yes	1.0	1.1
SETD1A†	NM_014712.3	Epilepsy, early-onset, with or without developmental delay, 618832 (3)	Yes	1.0	1.8
SETD1B†	NM_001353345.2	Intellectual developmental disorder with seizures and language delay, 619000 (3)	Unknown	1.0	4.7
SETD2	NM_014159.7	Renal Cell Carcinoma, Nonpapillary (RCC)	Unknown	1.0	3.0
SETD5†	NM_001080517.3	Intellectual Developmental Disorder, autosomal dominant 23, 615761 (3)	Yes	1.0	1.1
SGSH	NM_000199.5	Mucopolysaccharidosis type IIIA (Sanfilippo A), 252900 (3)	No	0.0	-0.1
SHANK3†	NM_001372044.2	Phelan-McDermid syndrome, 606232 (3); Schizophrenia 15, 613950 (3)	Yes	1.0	3.7
SHH	NM_000193.4	Microphthalmia, Isolated, With Coloboma 5 (MCOPCB5); Polydactyly, Preaxial Ii (PPD2); Solitary Median Maxillary Central Incisor (SMMCI); Holoprosencephaly 3 (HPE3)	Yes	1.0	2.9
SHROOM4	NM_020717.5	Stocco Dos Santos X-Linked Intellectual Developmental Disorder Syndrome; Stocco Dos Santos X-Linked Intellectual Developmental Disorder Syndrome	Unknown	1.0	1.0
SIK1†	NM_173354.5	Epileptic encephalopathy, early infantile, 30, 616341 (3)	Unknown	0.9	1.5
SIN3A	NM_001145358.2	Witteveen-Kolk syndrome, 613406 (3)	Yes	1.0	4.4
SIX3	NM_005413.4	Holoprosencephaly 2 (HPE2)	Yes	0.9	2.1
SKI†	NM_003036.4	Shprintzen-Goldberg syndrome, 182212 (3) formerly mapped to 1q22-q24	Unknown	1.0	1.5
SLC12A1	NM_000338.3	Bartter Syndrome, Antenatal, Type 1	No	0.0	1.2
SLC12A3	NM_001126108.2	Gitelman Syndrome	No	0.0	-0.9
SLC12A5	NM_020708.5	Epileptic encephalopathy, early infantile, 34, 616645 (3); Epilepsy, idiopathic generalized, susceptibility to, 14, 616685 (3)	Unknown	1.0	4.7
SLC12A6	NM_001365088.1	Agenesis of the Corpus Callosum With Peripheral Neuropathy (ACCPN)	Unknown	0.0	3.0
SLC13A5	NM_177550.5	Epileptic encephalopathy, early infantile, 25, 615905 (3)	No	0.0	1.1
SLC16A1	NM_003051.4	Hyperinsulinemic Hypoglycemia, Familial, 7 (HHF7); Erythrocyte Lactate Transporter Defect	Unknown	0.0	1.7
SLC16A2	NM_006517.5	Allan-Herndon-Dudley Syndrome (AHDS)	Yes	1.0	2.4
SLC17A5	NM_012434.5	Salla Disease (SD); Infantile Sialic Acid Storage Disease (ISSD)	No	0.0	0.7
SLC19A2	NM_006996.3	Thiamine-Responsive Megaloblastic Anemia Syndrome (TRMA)	Unknown	0.0	0.2
SLC19A3	NM_025243.4	Thiamine Metabolism Dysfunction Syndrome 2 (Biotin- Or Thiamine-Responsive	Unknown	0.1	-0.4
SLC1A2†	NM_004171.4	Epileptic encephalopathy, early infantile, 41, 617105 (3)	Unknown	0.7	2.3

SLC1A3	NM_004172.5	Episodic Ataxia, Type 6 (EA6)	Unknown	1.0	1.8
SLC1A4	NM_003038.5	Spastic tetraplegia, thin corpus callosum, and progressive microcephaly, 616657 (3)	Unknown	0.1	1.7
SLC20A2	NM_001257180.2	Basal ganglia calcification, idiopathic, 1, 213600 (3)	Unknown	1.0	2.6
SLC25A1	NM_005984.5	Combined D-2- And L-2-Hydroxyglutaric Aciduria (D2L2AD); Combined D-2- And L-2-Hydroxyglutaric Aciduria (D2L2AD)	Unknown	0.1	1.2
SLC25A12	NM_003705.5	Hypomyelination, Global Cerebral	Unknown	0.2	2.7
SLC25A15	NM_014252.4	Hyperornithinemia-Hyperammonemia-Homocitrullinuria Syndrome	Unknown	0.0	0.1
SLC25A19	NM_001126121.2	Thiamine Metabolism Dysfunction Syndrome 4 (Bilateral Striatal Degeneration; Microcephaly, Amish Type (MCPHA)	No	0.6	0.6
SLC25A20	NM_000387.6	Carnitine-Acylcarnitine Translocase Deficiency	Unknown	0.0	0.3
SLC25A22	NM_001191061.2	Epileptic Encephalopathy, Early Infantile, 3 (EIEE3)	Unknown	0.0	0.9
SLC25A4	NM_001151.4	Progressive External Ophthalmoplegia with Mitochondrial DNA Deletions,	Unknown	0.5	1.9
SLC2A1	NM_006516.4	Epilepsy, Idiopathic Generalized, Susceptibility To, 12 (EIG12); Glut1 Deficiency Syndrome 2 (GLUT1DS2); Glut1 Deficiency Syndrome 1 (GLUT1DS1); Dystonia 9 (DYT9)	Yes	1.0	2.9
SLC33A1	NM_004733.4	Congenital Cataracts, Hearing Loss, And Neurodegeneration (CCHLND); Spastic Paraplegia 42, Autosomal Dominant (SPG42)	Unknown	0.0	2.0
SLC35A1	NM_006416.5	Congenital Disorder of Glycosylation, Type Iif (CDG2F)	Unknown	0.7	1.3
SLC35A2	NM_005660.3	Congenital Disorder of Glycosylation, Type Iim (CDG2M); Congenital Disorder Of Glycosylation, Type Iim (CDG2M)	Yes	0.7	2.5
SLC35A3	NM_012243.3	Arthrogryposis, Intellectual Developmental Disorder, and seizures, 615553 (3) mutation identified in 1 family	Unknown	0.0	1.4
SLC35C1	NM_018389.5	Congenital disorder of glycosylation, type IIc, 266265 (3)	No	0.0	1.2
SLC37A4	NM_001467.6	Glycogen Storage Disease Ic; Glycogen Storage Disease Ib; Glycogen Storage Disease Ic; Glycogen Storage Disease Ib	No	0.0	0.0
SLC39A8	NM_001135146.2	Congenital disorder of glycosylation, type IIn, 616721 (3)	Unknown	0.7	1.1
SLC3A1	NM_000341.4	Hypotonia-Cystinuria Syndrome; Cystinuria	No	0.0	-3.1
SLC45A1	NM_001080397.3	Intellectual developmental disorder with neuropsychiatric features, 617532 (3)	Unknown	0.0	1.5
SLC46A1	NM_080669.6	Folate Malabsorption, Hereditary; Folate Malabsorption, Hereditary	Unknown	0.0	1.2
SLC5A6	NM_021095.4	Neurodegeneration, infantile-onset, biotin-responsive, 618973 (3)	Unknown	0.0	1.6
SLC6A1†	NM_003042.4	Myoclonic-atonic epilepsy, 616421 (3)	Yes	1.0	4.2
SLC6A19	NM_001003841.3	Iminoglycinuria; Hartnup Disorder; Hyperglycinuria	Unknown	0.0	0.0
SLC6A5	NM_004211.5	Hyperekplexia 3 (HKPX3)	Unknown	0.0	-0.1
SLC6A8	NM_005629.4	Cerebral Creatine Deficiency Syndrome 1 (CCDS1); Cerebral Creatine Deficiency Syndrome 1 (CCDS1)	Yes	1.0	3.0
SLC9A6	NM_001379110.1	Intellectual Developmental Disorder, X-Linked, Syndromic, Christianson Type (MRXSCH); Intellectual Developmental Disorder, X-Linked, Syndromic, Christianson Type (MRXSCH)	Yes	1.0	3.0
SLC9A9	NM_173653.4	Autism susceptibility 16, 613410 (3) mutation identified in 1 family	Unknown	0.0	-0.2
SMARCA2	NM_003070.5	Nicolaides-Baraitser Syndrome (NCBRS)	Unknown	1.0	5.1
SMARCA4†	NM_003072.5	Intellectual Developmental Disorder, Autosomal Dominant 16 (MRD16); Rhabdoid Tumor Predisposition Syndrome 2 (RTPS2)	Unknown	1.0	6.8
SMARCB1†	NM_003073.5	Intellectual Developmental Disorder, Autosomal Dominant 15 (MRD15); Rhabdoid Tumor Predisposition Syndrome 1 (RTPS1); Schwannomatosis	Yes	1.0	3.6
SMARCC2†	NM_001330288.2	Coffin-Siris syndrome 8, 618362 (3)	Unknown	1.0	3.9
SMARCE1	NM_003079.5	Meningioma, familial, susceptibility to, 607174 (3); Coffin-Siris syndrome 5, 616938 (3)	Unknown	1.0	2.7
SMC1A	NM_006306.4	Cornelia De Lange Syndrome 2 (CDLS2); Cornelia De Lange Syndrome 2 (CDLS2)	Yes	1.0	6.4
SMC3†	NM_005445.4	Cornelia De Lange Syndrome 3 (CDLS3)	Unknown	1.0	6.4
SMPD1	NM_000543.5	Niemann-Pick Disease, Type B; Niemann-Pick Disease, Type A	Unknown	0.0	-0.1
SMS	NM_004595.5	Intellectual Developmental Disorder, X-Linked, Syndromic, Snyder-Robinson Type (MRXSSR)	Yes	1.0	2.3
SNAP25†	NM_130811.4	Myasthenic syndrome, congenital, 18, 616330 (3) mutation identified in 1 CMS18 patient	Unknown	1.0	3.0
SNAP29	NM_004782.4	Cerebral Dysgenesis, Neuropathy, Ichthyosis, And Palmoplantar Keratoderma	Unknown	0.0	-0.9

SNIP1	NM_024700.4	Psychomotor Retardation, Epilepsy, And Craniofacial Dysmorphism	Unknown	0.4	1.2
SNRPN	NM_003097.6	Prader-Willi syndrome, 176270 (3)	Unknown	0.0	2.4
SNX14	NM_153816.6	Spinocerebellar ataxia, autosomal recessive 20, 616354 (3)	Unknown	0.0	1.8
SNX27	NM_001330723.2	Generalized hypotonia;Global developmental delay;Intellectual disability;Seizures	Unknown	1.0	2.2
SON†	NM_138927.4	ZTTK syndrome, 617140 (3)	Yes	1.0	1.5
SOX10	NM_006941.4	Waardenburg Syndrome, Type 4C (WS4C); Waardenburg Syndrome, Type 2E (WS2E); Peripheral Demyelinating Neuropathy, Central Dysmyelination, Waardenburg	Yes	1.0	2.8
SOX11	NM_003108.4	Coffin-Siris syndrome 9, 615866 (3)	Yes	0.9	2.3
SOX2	NM_003106.4	Microphthalmia, syndromic 3, 206900 (3); Optic nerve hypoplasia and abnormalities of the central nervous system, 206900 (3)	Yes	0.7	2.1
SOX3	NM_005634.3	Panhypopituitarism, X-Linked (PHPX); 46,XX Sex Reversal 3 (SRXX3); Intellectual Developmental Disorder, X-Linked, With Panhypopituitarism	Unknown	0.4	2.2
SOX5	NM_006940.6	Lamb-Shaffer syndrome, 616803 (3) some LAMSHF patients have 12p12 deletions	Yes	1.0	3.2
SPAST	NM_014946.4	Spastic Paraplegia 4, Autosomal Dominant (SPG4)	Yes	1.0	1.2
SPATA5	NM_145207.3	Epilepsy, hearing loss, and Intellectual Developmental Disorder syndrome, 616577 (3)	Unknown	0.0	0.5
SPATA7	NM_018418.5	Leber Congenital Amaurosis 3 (LCA3); Retinitis Pigmentosa (RP)	No	0.0	0.0
SPG11	NM_025137.4	Spastic Paraplegia 11, Autosomal Recessive (SPG11)	Unknown	0.0	-1.4
SPG7	NM_003119.4	Spastic Paraplegia 7, Autosomal Recessive (SPG7)	Unknown	0.0	-0.9
SPINK5	NM_006846.4	Netherton Syndrome (NETH)	Unknown	0.0	0.3
SPR	NM_003124.5	Dystonia, Dopa-Responsive, Due to Sepiapterin Reductase Deficiency	Unknown	0.0	0.7
SPRY4	NM_001127496.3	Hypogonadotropic Hypogonadism 17 with or without Anosmia (HH17)	Unknown	0.0	0.5
SPTAN1†	NM_001130438.3	Epileptic Encephalopathy, Early Infantile, 5 (EIEE5)	Unknown	1.0	5.5
SRCAP†	NM_006662.3	Floating-Harbor Syndrome (FLHS)	Uncertain	1.0	2.1
SRD5A3	NM_024592.5	Kahrizi Syndrome (KHRZ); Congenital Disorder of Glycosylation, Type Iq (CDG1Q)	Unknown	0.0	0.8
SRPX2	NM_014467.3	Rolandic Epilepsy, Intellectual Developmental Disorder, And Speech Dyspraxia, X-Linked	Unknown	0.0	0.2
SSR4	NM_006280.3	Congenital disorder of glycosylation, type Iy, 300934 (3)	Unknown	0.9	0.9
ST3GAL3	NM_006279.5	Epileptic Encephalopathy, Early Infantile, 15 (EIEE15); Intellectual Developmental Disorder, Autosomal Recessive 12 (MRT12)	Unknown	0.0	1.5
ST3GAL5	NM_003896.4	Amish Infantile Epilepsy Syndrome	No	0.0	1.0
STAG1†	NM_005862.3	Intellectual Developmental Disorder, autosomal dominant 47, 617635 (3)	Unknown	1.0	4.4
STAG2	NM_001042750.2	Mullegama-Klein-Martinez syndrome, 301022 (3); Holoprosencephaly 13, X-linked, 301043 (3)	Unknown	1.0	4.9
STAMBP	NM_213622.4	Microcephaly-Capillary Malformation Syndrome (MICCAP)	Unknown	0.0	0.9
STAR	NM_000349.3	Lipoid Congenital Adrenal Hyperplasia (LCAH)	Unknown	0.0	-0.1
STIL	NM_001048166.1	Microcephaly 7, Primary, Autosomal Recessive (MCPH7)	Unknown	0.2	1.1
STRADA	NM_001003787.4	Polyhydramnios, megalencephaly, and symptomatic epilepsy, 611087 (3)	Unknown	0.0	1.5
STS	NM_001320752.2	Ichthyosis, X-Linked (XLI)	Yes	0.8	1.1
STT3A	NM_152713.5	Congenital disorder of glycosylation, type Iw, 615596 (3)	No	0.0	3.6
STT3B	NM_178862.3	Congenital disorder of glycosylation, type Ix, 615597 (3) mutation identified in 1 family	Unknown	1.0	3.8
STX11	NM_003764.4	Hemophagocytic Lymphohistiocytosis, Familial, 4 (FHL4)	Unknown	0.0	-0.1
STX16†	NM_001001433.3	Pseudohypoparathyroidism, Type Ib (PHP1B)	Unknown	0.5	1.0
STX1B†	NM_052874.5	Generalized epilepsy with febrile seizures plus, type 9, 616172 (3)	Unknown	1.0	2.9
STXBP1†	NM_001032221.6	Epileptic Encephalopathy, Early Infantile, 4 (EIEE4)	Yes	1.0	4.3
STXBP2	NM_006949.4	Hemophagocytic Lymphohistiocytosis, Familial, 5 (FHL5)	Unknown	0.0	0.5
SUCLA2	NM_003850.3	Mitochondrial DNA Depletion Syndrome 5 (Encephalomyopathic with or without Methylmalonic Aciduria)	Unknown	0.0	1.0
SUCLG1	NM_003849.4	Mitochondrial DNA Depletion Syndrome 9 (Encephalomyopathic Type with or without Methylmalonic Aciduria)	No	0.0	-0.2
SUMF1	NM_182760.4	Multiple Sulfatase Deficiency (MSD)	No	0.0	-0.5
SUOX	NM_001032386.2	Sulfocysteinuria	Unknown	0.0	0.4
SURF1	NM_003172.4	Leigh Syndrome (LS); Leigh Syndrome (LS)	Unknown	0.0	-0.7

© 2023 Koh HY et al. *JAMA Network Open.*

SYN1	NM_006950.3	Epilepsy, X-Linked, With Variable Learning Disabilities and Behavior	Unknown	1.0	3.0
SYNGAP1†	NM_006772.3	Intellectual Developmental Disorder, Autosomal Dominant 5 (MRD5); Intellectual Developmental Disorder, Autosomal Dominant 5 (MRD5)	Yes	1.0	5.6
SYNJ1	NM_203446.3	Parkinson disease 20, early-onset, 615530 (3); Epileptic encephalopathy, early infantile, 53, 617389 (3)	Unknown	0.4	2.2
SYP	NM_003179.3	Intellectual Developmental Disorder, X-Linked 96 (MRX96); Intellectual Developmental Disorder, X-Linked 96 (MRX96)	Uncertain	0.9	1.3
SYT2	NM_177402.5	Myasthenic syndrome, congenital, 7, presynaptic, 616040 (3)	Unknown	1.0	2.0
SZT2	NM_001365999.1	Epileptic encephalopathy, early infantile, 18, 615476 (3)	Unknown	0.0	2.6
TACO1	NM_016360.4	Leigh Syndrome (LS)	Unknown	0.0	0.9
TACR3	NM_001059.3	Hypogonadotropic Hypogonadism 11 with or without Anosmia (HH11)	Unknown	0.0	0.6
TANC2†	NM_025185.4	Intellectual developmental disorder with autistic features and language delay, with or without seizures, 618906 (3)	Unknown	1.0	2.3
TANGO2	NM_152906.7	Metabolic encephalomyopathic crises, recurrent, with rhabdomyolysis, cardiac arrhythmias, and neurodegeneration, 616878 (3)	Unknown	0.0	0.4
TAT	NM_000353.3	Tyrosinemia, Type Ii	Unknown	0.0	0.8
TAZ	NM_000116.5	Barth Syndrome (BTHS); Barth Syndrome (BTHS)	Unknown	0.7	2.2
TBC1D20	NM_144628.4	Warburg micro syndrome 4, 615663 (3)	No	0.9	1.2
TBC1D24	NM_001199107.2	Myoclonic epilepsy, infantile, familial, 605021 (3); Epileptic encephalopathy, early infantile, 16, 615338 (3); DOORS syndrome, 220500 (3); Deafness, autosomal recessive 86, 614617 (3); Deafness, autosomal dominant 65, 616044 (3); Epilepsy, rolandic, with proxysmal exercise-induce dystonia and writer's cramp, 608105 (3)	Unknown	0.0	0.8
TBCD	NM_005993.5	Encephalopathy, progressive, early-onset, with brain atrophy and thin corpus callosum, 617193 (3)	Unknown	0.0	1.8
TBCE	NM_003193.5	Kenny-Caffey Syndrome, Type 1 (KCS1); Hypoparathyroidism-Retardation-Dysmorphism Syndrome (HRD)	No	0.0	0.1
TBCK	NM_001163435.3	Hypotonia, infantile, with psychomotor retardation and characteristic facies 3, 616900 (3)	Unknown	0.0	0.5
TBL1XR1†	NM_024665.7	Intellectual Developmental Disorder, autosomal dominant 41, 616944 (3); Pierpont syndrome, 602342 (3)	Yes	1.0	4.2
TBR1†	NM_006593.4	Intellectual developmental disorder with autism and speech delay, 606053 (3)	Yes	1.0	3.6
TBX1†	NM_001379200.1	Conotruncal Heart Malformations (CTHM); Velocardiofacial Syndrome; Digeorge Syndrome (DGS)	Unknown	0.8	0.7
TBX19	NM_005149.3	ACTH Deficiency, Isolated (IAD)	Unknown	0.0	0.6
TCF4†	NM_001083962.2	Pitt-Hopkins Syndrome (PTHS)	Yes	1.0	4.1
TCTN1	NM_001082538.3	Joubert Syndrome 13 (JBTS13)	No	0.0	0.6
TCTN2	NM_024809.5	Meckel Syndrome, Type 8 (MKS8)	No	0.0	0.2
TCTN3	NM_015631.6	Joubert Syndrome 18 (JBTS18); Orofaciodigital Syndrome Iv (OFD4)	Unknown	0.0	0.8
TDGF1	NM_003212.4	Teratocarcinoma-Derived Growth Factor 1 (TDGF1)	Uncertain	0.0	-0.1
TDP2	NM_016614.3	Spinocerebellar ataxia, autosomal recessive 23, 616949 (3)	Unknown	0.0	0.1
TECPR2	NM_014844.5	Spastic Paraplegia 49, Autosomal Recessive (SPG49)	No	0.6	1.7
TECR	NM_138501.6	Intellectual Developmental Disorder, Autosomal Recessive 14 (MRT14)	Unknown	0.1	1.8
TELO2	NM_016111.4	You-Hoover-Fong syndrome, 616954 (3)	Unknown	0.0	-0.4
TET3	NM_001287491.2	Beck-Fahrner syndrome, 618798 (3)	Unknown	1.0	2.9
TGDS	NM_014305.4	Catel-Manzke syndrome, 616145 (3)	Unknown	0.0	0.3
TGFB1	NM_000660.7	Camurati-Engelmann Disease (CAEND)	Unknown	0.0	1.9
TGFBR2†	NM_003242.6	Colorectal Cancer, Hereditary Nonpolyposis, Type 6 (HNPCC6); Loeys-Dietz Syndrome, Type 2B (LDS2B); Loeys-Dietz Syndrome, Type 1B (LDS1B); Esophageal Cancer	Unknown	0.1	2.2
TGIF1	NM_003244.4	Holoprosencephaly 4 (HPE4)	Yes	0.0	0.2
THOC2	NM_001081550.2	Intellectual Developmental Disorder, X-linked 12/35, 300957 (3)	Unknown	1.0	5.5
TIMM50	NM_001001563.5	3-methylglutaconic aciduria, type IX, 617698 (3)	Unknown	0.0	0.0
TK2	NM_004614.5	Mitochondrial DNA Depletion Syndrome 2 (Myopathic Type) (MTDPS2)	Unknown	0.0	0.2
TMEM138	NM_016464.5	Joubert Syndrome 16 (JBTS16)	Unknown	0.0	0.5

© 2023 Koh HY et al. *JAMA Network Open.*

TMEM165	NM_018475.5	Congenital Disorder of Glycosylation, Type Iik (CDG2K)	Unknown	0.3	1.4
TMEM216	NM_001173990.3	Joubert syndrome 2, 608091 (3); Meckel syndrome 2, 603194 (3)	Unknown	0.0	0.5
TMEM231	NM_001077418.3	Joubert Syndrome 20 (JBTS20)	No	0.0	-0.4
TMEM237	NM_001044385.3	Joubert Syndrome 14 (JBTS14)	No	0.0	0.1
TMEM67	NM_153704.6	Nephronophthisis 11 (NPHP11); Joubert Syndrome 6 (JBTS6); Meckel Syndrome, Type 3 (MKS3); Coach Syndrome; Bardet-Biedl Syndrome (BBS)	No	0.0	0.5
TMEM70	NM_017866.6	Mitochondrial Complex V (Atp Synthase) Deficiency, Nuclear Type 2	Unknown	0.0	0.1
TMLHE	NM_018196.4	Epsilon-Trimethyllysine Hydroxylase Deficiency (TMLHED); Epsilon-Trimethyllysine Hydroxylase Deficiency (TMLHED)	Uncertain	0.0	0.6
TMX2	NM_015959.4	Neurodevelopmental disorder with microcephaly, cortical malformations, and spasticity, 618730 (3)	Unknown	0.6	0.2
TNK2	NM_001382273.1	severe autosomal recessive infantile onset epilepsy;EE	Unknown	0.0	-0.2
TPK1	NM_022445.4	Thiamine Metabolism Dysfunction Syndrome 5 (Episodic Encephalopathy	No	0.1	0.3
TPP1	NM_000391.4	Ceroid Lipofuscinosis, Neuronal, 2 (CLN2)	Unknown	0.0	-0.2
TRAF3IP2	NM_147686.4	Psoriasis Susceptibility 13 (PSORS13)	Unknown	0.0	1.6
TRAF7†	NM_032271.3	Cardiac, facial, and digital anomalies with developmental delay, 618164 (3)	Unknown	0.0	3.3
TRAK1	NM_001042646.3	Epileptic encephalopathy, early infantile, 68, 618201 (3)	Unknown	0.0	1.1
TRAPPC12	NM_016030.6	Encephalopathy, progressive, early-onset, with brain atrophy and spasticity, 617669 (3)	Unknown	0.0	0.2
TRAPPC4	NM_016146.6	Neurodevelopmental disorder with epilepsy, spasticity, and brain atrophy, 618741 (3)	Unknown	0.0	0.4
TRAPPC6B	NM_001079537.2	Neurodevelopmental disorder with microcephaly, epilepsy, and brain atrophy, 617862 (3)	Unknown	0.0	0.1
TRAPPC9	NM_001160372.4	Intellectual Developmental Disorder, Autosomal Recessive 13 (MRT13)	No	0.0	1.5
TREM2	NM_018965.4	Polycystic Lipomembranous Osteodysplasia with Sclerosing Leukoencephalopathy	Unknown	0.0	0.2
TREX1	NM_033629.6	Chilblain Lupus 1 (CHBL1); Aicardi-Goutieres Syndrome 1 (AGS1); Vasculopathy, Retinal, With Cerebral Leukodystrophy (RVCL); Systemic Lupus Erythematosus (SLE)	Unknown	0.6	-0.8
TRIM8	NM_030912.3	Early-onset epileptic encephalopathy (EOEE);EE;Seizures	Unknown	1.0	2.7
TRIO†	NM_007118.4	Intellectual developmental disorder, autosomal dominant 44, with microcephaly, 617061 (3); Intellectual developmental disorder, autosomal dominant 63, with macrocephaly, 618825 (3)	Yes	1.0	5.3
TRIP13	NM_004237.4	Mosaic variegated aneuploidy syndrome 3, 617598 (3); Oocyte maturation defect 9, 619011 (3)	Unknown	1.0	2.3
TRIT1	NM_017646.6	Combined oxidative phosphorylation deficiency 35, 617873 (3)	Unknown	0.0	0.8
TRMT10A	NM_001134665.3	Microcephaly, short stature, and impaired glucose metabolism 1	No	0.0	0.4
TRNT1	NM_182916.3	Sideroblastic anemia with B-cell immunodeficiency, periodic fevers, and developmental delay, 616084 (3); Retinitis pigmentosa and erythrocytic microcytosis, 616959 (3) pseudogenes on chromosomes 1 and 22	Unknown	0.0	-1.1
TRPM3	NM_001366145.2	Generalized hypotonia;Global developmental delay;Intellectual disability;Seizures;Autistic behavior	Unknown	0.0	3.2
TRPM6	NM_017662.5	Hypomagnesemia 1, Intestinal (HOMG1)	Unknown	0.0	1.6
TRRAP	NM_001375524.1	Developmental delay with or without dysmorphic facies and autism, 618454 (3); Deafness, autosomal dominant 75, 618778 (3) mutation identified in 1 DFNA75 family	Uncertain	1.0	8.2
TSC1†	NM_000368.5	Focal Cortical Dysplasia of Taylor (FCDT); Tuberous Sclerosis 1 (TSC1)	Unknown	1.0	2.3
TSC2†	NM_000548.5	Tuberous sclerosis 2, 613254 (3); Lymphangioleiomyomatosis, somatic, 606690 (3); Focal cortical dysplasia, type II, somatic, 607341 (3) somatic mutation identified in 1 FCORD2 patient	Yes	1.0	-0.3
TSEN15	NM_052965.4	Pontocerebellar hypoplasia, type 2F	Unknown	0.3	1.2
TSEN2	NM_025265.4	Pontocerebellar Hypoplasia, Type 2B (PCH2B)	No	0.0	-1.0
TSEN34	NM_001077446.4	Pontocerebellar Hypoplasia, Type 2C (PCH2C);	Unknown	0.4	-1.0
TSEN54	NM_207346.3	Pontocerebellar Hypoplasia, Type 2A (PCH2A); Pontocerebellar Hypoplasia, Type 4 (PCH4)	Unknown	0.0	-0.1
TSFM	NM_005726.6	Combined Oxidative Phosphorylation Deficiency 3 (COXPD3)	Unknown	0.0	-0.2
TSPAN7	NM_004615.4	Intellectual Developmental Disorder, X-Linked 58 (MRX58)	Unknown	0.7	1.9
TUBA1A	NM_006009.4	Lissencephaly 3 (LIS3)	Unknown	1.0	5.6
TUBA3E	NM_207312.3	Global developmental delay, primary microcephaly, lissencephaly, epilepsy	Unknown	0.0	-0.3

© 2023 Koh HY et al. *JAMA Network Open.*

TUBB2A	NM_001069.3	Cortical dysplasia, complex, with other brain malformations 5, 615763 (3)	Unknown	0.9	5.3
TUBB2B	NM_178012.5	Polymicrogyria, Symmetric or Asymmetric (PMGYSA)	Unknown	1.0	5.1
TUBB3	NM_006086.4	Cortical Dysplasia, Complex, With Other Brain Malformations 1 (CDCBM1); Fibrosis Of Extraocular Muscles, Congenital, 3A, With Or Without Extraocular	Unknown	1.0	4.6
TUBB4A	NM_006087.4	Leukodystrophy, Hypomyelinating, 6 (HLD6); Dystonia 4, Torsion, Autosomal Dominant (DYT4)	Unknown	0.1	4.3
TUBG1	NM_001070.5	Cortical dysplasia, complex, with other brain malformations 4, 615412 (3)	Unknown	0.1	4.2
TUBGCP4	NM_014444.5	Microcephaly and chorioretinopathy, autosomal recessive, 3, 616335 (3)	Unknown	0.0	2.6
TUBGCP6	NM_020461.4	Microcephaly And Chorioretinopathy with or without Impaired Intellectual Development	No	0.0	-0.9
TUSC3	NM_006765.4	Intellectual Developmental Disorder, Autosomal Recessive 7 (MRT7)	Unknown	0.0	0.0
TXN2	NM_012473.4	Combined oxidative phosphorylation deficiency 29, 616811 (3) mutation identified in 1 COXPD29 patient	Unknown	0.0	0.6
TYROBP	NM_003332.4	Polycystic Lipomembranous Osteodysplasia with Sclerosing Leukoencephalopathy	No	0.2	-0.3
UBA5	NM_024818.6	Epileptic encephalopathy, early infantile, 44, 617132 (3); Spinocerebellar ataxia, autosomal recessive 24, 617133 (3) mutation identified in 1 SCAR24 family	Unknown	0.0	1.3
UBE2A	NM_003336.4	Intellectual Developmental Disorder, X-Linked, Syndromic, Nascimento Type (MRXSN)	Yes	0.8	2.5
UBE3A†	NM_130839.5	Angelman Syndrome (AS)	Yes	1.0	4.4
UBTF	NM_014233.4	Neurodegeneration, childhood-onset, with brain atrophy, 617672 (3)	Unknown	1.0	4.7
UFC1	NM_016406.4	Neurodevelopmental disorder with spasticity and poor growth, 618076 (3)	Unknown	0.0	0.2
UFM1	NM_016617.4	Leukodystrophy, hypomyelinating, 14	Unknown	0.2	0.6
UGDH	NM_003359.4	Epileptic encephalopathy, early infantile, 84, 618792 (3)	Unknown	0.0	2.3
UGP2	NM_006759.4	Epileptic encephalopathy, early infantile, 83, 618744 (3)	Unknown	0.0	1.9
UNC13D	NM_199242.3	Hemophagocytic Lymphohistiocytosis, Familial, 3 (FHL3)	Unknown	0.0	-0.2
UNC80	NM_001371986.1	Hypotonia, infantile, with psychomotor retardation and characteristic facies 2, 616801 (3)	Unknown	0.1	5.5
UPB1	NM_016327.3	Beta-Ureidopropionase Deficiency	Unknown	0.0	-0.9
UPF3B	NM_080632.3	Intellectual Developmental Disorder, X-Linked, Syndromic 14 (MRXS14)	Yes	1.0	1.8
UQCC2	NM_032340.4	Mitochondrial complex III deficiency, nuclear type 7, 615824 (3)	Unknown	0.0	0.5
USP7	NM_003470.3	Hao-Fountain syndrome, 616863 (3)	Unknown	1.0	5.6
USP9X	NM_001039591.3	Intellectual Developmental Disorder, X-linked 99, 300919 (3); Intellectual Developmental Disorder, X-linked 99, syndromic, female-restricted, 300968 (3)	Yes	1.0	6.4
VAMP2	NM_014232.3	Neurodevelopmental disorder with hypotonia and autistic features with or without hyperkinetic movements, 618760 (3)	Unknown	0.9	1.4
VARS	NM_006295.3	Neurodevelopmental Disorder with Microcephaly, Seizures, and Cortical Atrophy, 617802 (3)	Unknown	0.0	2.7
VARS1	NM_006295.3	Neurodevelopmental Disorder with Microcephaly, Seizures, and Cortical Atrophy	Unknown	N/A	N/A
VCP	NM_007126.5	Inclusion body myopathy with early-onset Paget disease and frontotemporal dementia 1, 167320 (3); Amyotrophic lateral sclerosis 14, with or without frontotemporal dementia, 613954 (3); Charcot-Marie-Tooth disease, type 2Y, 616687 (3)	Unknown	1.0	5.4
VDR	NM_000376.3	Mycobacterium Tuberculosis, Susceptibility To; Vitamin D-Dependent Rickets, Type 2A (VDDR2A)	Unknown	0.0	1.1
VLDLR	NM_003383.5	Cerebellar Hypoplasia and Intellectual Developmental Disorder with or without quadrupedal locomotion 1, 224050 (3)	Unknown	0.0	-1.1
VPS11	NM_021729.6	Leukodystrophy, hypomyelinating, 12, 616683 (3)	Unknown	0.0	2.3
VPS13A	NM_033305.3	Choreoacanthocytosis (CHAC)	Unknown	0.0	1.8
VPS13B	NM_152564.5	Cohen syndrome, 216550 (3)	No	0.0	1.0
VPS53	NM_001128159.3	Pontocerebellar hypoplasia, type 2E, 615851 (3)	Unknown	0.0	1.6
WAC	NM_016628.5	Desanto-Shinawi syndrome, 616708 (3)	Yes	1.0	1.5
WARS2	NM_015836.4	Neurodevelopmental disorder, mitochondrial, with abnormal movements and lactic acidosis, with or without seizures, 617710 (3)	Unknown	0.1	0.9
WASF1†	NM_003931.3	Neurodevelopmental disorder with absent language and variable seizures, 618707 (3)	Unknown	1.0	2.5
WASHC4	NM_015275.3	Intellectual Developmental Disorder, Autosomal Recessive 43	Unknown	N/A	N/A
WDR11	NM_018117.12	Hypogonadotropic Hypogonadism 14 with or without Anosmia (HH14)	Unknown	0.0	1.8
WDR26†	NM_001379403.1	Skraban-Deardorff syndrome, 617616 (3)	Unknown	1.0	3.6

© 2023 Koh HY et al. *JAMA Network Open.*

WDR37	NM_014023.4	Neurooculocardiogenitourinary syndrome, 618652 (3)	Unknown	0.6	2.3
WDR4	NM_018669.6	Microcephaly, growth deficiency, seizures, and brain malformations, 618346 (3); Galloway-Mowat syndrome 6, 618347 (3)	Unknown	0.0	-1.0
WDR45	NM_001029896.2	Neurodegeneration With Brain Iron Accumulation 5 (NBIA5); Neurodegeneration with Brain Iron Accumulation 5 (NBIA5)	Yes	1.0	2.0
WDR45B	NM_019613.4	Neurodevelopmental disorder with spastic quadriplegia and brain abnormalities with or without seizures, 617977 (3)	Unknown	0.2	1.0
WDR62	NM_001083961.2	Microcephaly 2, Primary, Autosomal Recessive, with or without Cortical	Unknown	0.0	0.7
WDR73	NM_032856.5	Galloway-Mowat syndrome 1, 251300 (3)	Unknown	0.0	-0.5
WDR81	NM_001163809.2	Cerebellar ataxia, Intellectual Developmental Disorder, and dysequilibrium syndrome 2, 610185 (3); Hydrocephalus, congenital, 3, with brain anomalies, 617967 (3)	Unknown	0.0	2.1
WFS1	NM_006005.3	Wolfram-Like Syndrome, Autosomal Dominant (WFSL); Deafness, Autosomal Dominant 6 (DFNA6); Wolfram Syndrome 1 (WFS1)	Unknown	0.0	-4.7
WWOX	NM_016373.4	Esophageal Cancer	No	0.0	-4.4
XK	NM_021083.4	Mcleod Syndrome (MCLDS); Mcleod Syndrome (MCLDS)	Unknown	1.0	2.1
XPA	NM_000380.4	Xeroderma Pigmentosum, Complementation Group A (XPA)	No	0.0	0.3
XPC	NM_004628.5	Xeroderma pigmentosum, group C, 278720 (3)	Unknown	0.0	0.7
XPNPEP3	NM_022098.4	Nephronophthisis-Like Nephropathy 1 (NPHPL1)	No	0.0	-0.1
YWHAG†	NM_012479.4	Epileptic encephalopathy, early infantile, 56, 617665 (3)	Unknown	1.0	3.0
ZBTB18†	NM_205768.3	Intellectual Developmental Disorder, autosomal dominant 22, 612337 (3)	Yes	1.0	3.4
ZC3H14	NM_024824.5	Intellectual Developmental Disorder, autosomal recessive 56, 617125 (3)	Unknown	0.9	1.5
ZC4H2	NM_018684.4	Wieacker-Wolff syndrome, 314580 (3); Wieacker-Wolff syndrome, female-restricted, 301041 (3)	Yes	0.9	1.5
ZDHHC9	NM_016032.4	Intellectual Developmental Disorder, X-Linked, Syndromic, Raymond Type (MRXSR)	Unknown	0.7	2.6
ZEB2†	NM_014795.4	Mowat-Wilson Syndrome (MOWS)	Yes	1.0	3.9
ZFP57	NM_001109809.5	Diabetes Mellitus, Transient Neonatal, 1	Unknown	0.0	1.4
ZIC2	NM_007129.5	Holoprosencephaly 5 (HPE5)	Yes	1.0	3.2
ZMIZ1†	NM_020338.4	Neurodevelopmental disorder with dysmorphic facies and distal skeletal anomalies, 618659 (3)	Unknown	1.0	3.4
ZMYND11†	NM_001370100.5	Intellectual Developmental Disorder, autosomal dominant 30, 616083 (3)	Yes	1.0	3.7
ZNF142	NM_001379659.1	Neurodevelopmental disorder with impaired speech and hyperkinetic movements, 618425 (3)	Unknown	0.0	0.2
ZNF335	NM_022095.4	Microcephaly 10, Primary, Autosomal Recessive (MCPH10)	Unknown	0.0	0.5
ZNF41	NM_001324144.2	Intellectual Developmental Disorder, X-Linked 89 (MRX89)	Uncertain	0.0	1.3
ZNF592	NM_014630.3	Spinocerebellar Ataxia, Autosomal Recessive 5 (SCAR5)	No	1.0	0.8
ZNF674	NM_001190417.2	Intellectual Developmental Disorder, X-Linked 92 (MRX92)	Uncertain	0.0	1.1
ZNF711	NM_001330574.2	Intellectual Developmental Disorder, X-Linked 97 (MRX97)	Unknown	1.0	2.4
ZNF81	NM_007137.5	Intellectual Developmental Disorder, X-Linked 45 (MRX45)	Unknown	0.6	1.4
ZSWIM6†	NM_020928.2	Acromelic frontonasal dysostosis, 603671 (3); Neurodevelopmental disorder with movement abnormalities, abnormal gait, and autistic features, 617865 (3)	Unknown	1.0	4.2

© 2023 Koh HY et al. *JAMA Network Open.*

eTable 3. Nondiagnostic VUS in Epilepsy-Related Genes

Loss-of-function variants

Gene	DNA	Protein	Consequence/*In silico* prediction	Cases	Seizure onset	Sex	Epilepsy	ID	ASD
CACNA1B	c.925C>T	p.Gln309Ter	Stop gained	0316	5y	male	DEE	Mild	No
CACNA1B	c.4711C>T	p.Arg1571Ter	Stop gained	0736	3y	male	GGE	N	No
CCM2	c.632dupT	p.Glu212GlyfsTer45	Frameshift	0724	2y11m	male	GGE	N	No
POGZ	c.885A>G		Splice acceptor	0847	infantile onset	male	DEE	N	No
PUM1	c.1253-4G>T		Splice acceptor	0852	2y	female	NAFE	N	No
PUM1	c.3552delT	p.Asn1185MetfsTer34	Frameshift	0833	7y	male	NAFE	Moderate	Yes
TANC2	c.211+1G>A		Splice donor	0499	2y6m	female	Combined	Borderline	Yes
SMARCC2	c.2217delC	p.Thr740ArgfsTer17	Frameshift	0590	2y	female	NAFE	N	No

Missense variants

Gene	DNA	Protein	Consequence/*In silico* prediction	Cases	Seizure onset	Sex	Epilepsy	ID	ASD
ADNP	c.1668G>C	p.Gln556His	CADD (23.1), SIFT (D), PolyPhen (P), M-CAP (T), REVEL (0.2)	0607	7y	female	NAFE	N	No
AKT1	c.250A>T	p.Ile84Phe	CADD (25.8), SIFT (D), PolyPhen (D), M-CAP (D), REVEL (0.8)	0469	3y	male	GGE	Borderline	No
ALX4	c.409C>T	p.Leu137Phe	CADD (23.9), SIFT (D), PolyPhen (B), M-CAP (D), REVEL (0.3)	0565	1w (at birth)	male	DEE	Profound	No
ANKRD11	c.6437A>C	p.Lys2146Thr	CADD (22.8), SIFT (D), PolyPhen (B), M-CAP (D), REVEL (0.2)	0491	1y6m	male	DEE	Mild	No
ANKRD11	c.6950C>T	p.Pro2317Leu	CADD (22.7), SIFT (D), PolyPhen (B), M-CAP (D), REVEL (0.1)	0929	7y	male	NAFE	N	No
ANKRD11	c.6970C>G	p.Pro2324Ala	CADD (22.5), SIFT (D), PolyPhen (B), M-CAP (D), REVEL (0.1)	0753	2y	female	Combined	N	No
ARID1A	c.208G>A	p.Gly70Arg	CADD (23.9), SIFT (T), PolyPhen (P), M-CAP (D), REVEL (0.1)	1018	3m	male	DEE	Severe	Yes
ARID1A	c.3727G>A	p.Asp1243Asn	CADD (26.8), SIFT (D), PolyPhen (B), M-CAP (T), REVEL (0.2)	0600	2y	female	Combined	N	No
ARID1A	c.3974C>G	p.Pro1325Arg	CADD (25.1), SIFT (D), PolyPhen (D), M-CAP (T), REVEL (0.2)	0627	2y6m	male	NAFE	Borderline	No
ARID1B	c.1337C>T	p.Ala446Val	CADD (23.1), SIFT (T), PolyPhen (B), M-CAP (D), REVEL (0)	1092	4y	male	GGE	N	No
ARID1B	c.6064G>A	p.Glu2022Lys	CADD (24), SIFT (D), PolyPhen (B), M-CAP (T), REVEL (0.1)	0455	7y	female	IGE	N	No
ATN1	c.689G>A	p.Gly230Glu	CADD (22.8), SIFT (D), PolyPhen (B), M-CAP (D), REVEL (0.4)	0714	6y	female	IGE	N	No
ATP1A2	c.1283G>A	p.Arg428His	CADD (31), SIFT (D), PolyPhen (D), M-CAP (D), REVEL (0.8)	0513	11y	female	GGE	N	No
ATP1A3	c.2564T>G	p.Met855Arg	CADD (25), SIFT (T), PolyPhen (P), M-CAP (D), REVEL (0.9)	0721	1y	male	DEE	Moderate	Yes
ATP1A3	c.488C>A	p.Ser163Tyr	CADD (27.2), SIFT (D), PolyPhen (D), M-CAP (D), REVEL (0.9)	0973	9y	female	IGE	N	No
ATXN10	c.1115C>T	p.Ser372Phe	CADD (26.6), SIFT (D), PolyPhen (D), M-CAP (D), REVEL (0.3)	0541	7y	male	GGE	N	No
AUTS2	c.2956C>T	p.Arg986Trp	CADD (25.3), SIFT (D), PolyPhen (D), M-CAP (D), REVEL (0.3)	0474	11m	male	GGE	Borderline	No
CACNA1B	c.4384T>C	p.Tyr1462His	CADD (26.6), SIFT (D), PolyPhen (D), M-CAP (D), REVEL (0.9)	0962	12y	male	GGE	N	No
CACNA1E	c.5810C>T	p.Ser1937Leu	CADD (24.8), SIFT (D), PolyPhen (B), M-CAP (D), REVEL (0.4)	0890	13y	male	Combined	N	No
CACNA1G	c.2486G>A	p.Gly829Asp	CADD (28.7), SIFT (D), PolyPhen (D), M-CAP (D), REVEL (0.9)	0431	9m	male	DEE	Severe	No
CCM2	c.1206C>G	p.Asp402Glu	CADD (25.2), SIFT (D), PolyPhen (D), M-CAP (D), REVEL (0.2)	0871	2y	male	Combined	N	No
CDH15	c.1943G>A	p.Arg648Gln	CADD (32), SIFT (D), PolyPhen (D), M-CAP (D), REVEL (0.7)	0918	5y	male	NAFE	N	No
CDON	c.2242G>A	p.Gly748Ser	CADD (25.9), SIFT (D), PolyPhen (D), M-CAP (D), REVEL (0.5)	0702	6y	female	NAFE	Borderline	No

© 2023 Koh HY et al. *JAMA Network Open.*

CDON	c.3208T>C	p.Tyr1070His	CADD (23.4), SIFT (D), PolyPhen (P), M-CAP (D), REVEL (0.1)	0808	3y	female	NAFE	N	No
CHAMP1	c.2159A>G	p.Lys720Arg	CADD (26.2), SIFT (D), PolyPhen (D), M-CAP (T), REVEL (0.3)	1030	3m	female	DEE	N	No
CHD1	c.117T>G	p.Ser39Arg	CADD (23.6), SIFT (), PolyPhen (), M-CAP (D), REVEL (0.5)	0458	11y	female	NAFE	Mild	Yes
CHD2	c.5211C>G	p.His1737Gln	CADD (24.5), SIFT (D), PolyPhen (D), M-CAP (D), REVEL (0.5)	0701	14y	female	GGE	N	No
CHRNA2	c.710C>T	p.Thr237Met	CADD (26.7), SIFT (D), PolyPhen (P), M-CAP (D), REVEL (0.4)	0643	2y	female	NAFE	N	No
CHRNA4	c.869T>A	p.Leu290Gln	CADD (29.2), SIFT (D), PolyPhen (D), M-CAP (D), REVEL (0.9)	0691	2w	male	DEE	Severe	No
CIC	c.6272C>G	p.Ala2091Gly	CADD (25), SIFT (D), PolyPhen (D), M-CAP (D), REVEL (0.1)	0847	infantile onset	male	IGE	N	No
CLTC	c.1108C>T	p.Arg370Trp	CADD (27.8), SIFT (D), PolyPhen (D), M-CAP (D), REVEL (0.5)	0807	10m	female	GGE	Borderline	No
COL4A2	c.2453G>A	p.Gly818Glu	CADD (25.2), SIFT (D), PolyPhen (D), M-CAP (D), REVEL (0.9)	1019	9m	female	NAFE	Moderate	No
COL4A2	c.2498G>T	p.Gly833Val	CADD (24.5), SIFT (D), PolyPhen (D), M-CAP (D), REVEL (1)	0792	2y	female	NAFE	N	No
CREBBP	c.2810C>G	p.Pro937Arg	CADD (23.3), SIFT (D), PolyPhen (B), M-CAP (T), REVEL (0.3)	1036	3y	male	NAFE	N	No
CREBBP	c.6423C>G	p.Asn2141Lys	CADD (23), SIFT (D), PolyPhen (B), M-CAP (T), REVEL (0.3)	0884	4y	male	NAFE	Borderline	No
CTNNB1	c.1760G>A	p.Arg587Gln	CADD (32), SIFT (D), PolyPhen (D), M-CAP (D), REVEL (0.6)	0643	2y	female	NAFE	N	No
DEPDC5	c.1720A>G	p.Arg574Gly	CADD (24.4), SIFT (D), PolyPhen (D), M-CAP (D), REVEL (0.2)	0571	1y2m	male	NAFE	N	No
DEPDC5	c.4675C>T	p.Arg1559Cys	CADD (28.4), SIFT (D), PolyPhen (D), M-CAP (D), REVEL (0.4)	0676	1y	male	NAFE	N	No
DIAPH1	c.1409A>G	p.Asp470Gly	CADD (32), SIFT (D), PolyPhen (D), M-CAP (D), REVEL (0.5)	0809	1y	male	DEE	Borderline	No
DIAPH1	c.2378C>T	p.Ser793Phe	CADD (29.3), SIFT (D), PolyPhen (D), M-CAP (T), REVEL (0.4)	0432	14y	male	DEE	Moderate	Yes
DLL1	c.105G>C	p.Lys35Asn	CADD (24.8), SIFT (T), PolyPhen (D), M-CAP (D), REVEL (0.6)	0991	16y	male	GGE	N	No
DNMT3A	c.625C>T	p.Arg209Cys	CADD (29.1), SIFT (D), PolyPhen (P), M-CAP (D), REVEL (0.6)	0622	6m	male	NAFE	N	No
DYNC1H1	c.5417G>A	p.Arg1806Gln	CADD (23.9), SIFT (T), PolyPhen (B), M-CAP (T), REVEL (0.2)	0668	10y	female	NAFE	N	No
DYNC1H1	c.6616A>G	p.Lys2206Glu	CADD (32), SIFT (D), PolyPhen (D), M-CAP (D), REVEL (0.7)	0603	9y	female	GGE	N	No
EEF1A2	c.358G>A	p.Val120Met	CADD (24.4), SIFT (D), PolyPhen (P), M-CAP (D), REVEL (0.4)	0927	1y	female	GGE	N	Yes
EFTUD2	c.658C>T	p.Arg220Cys	CADD (32), SIFT (D), PolyPhen (D), M-CAP (D), REVEL (0.7)	1039	6y	female	IGE	N	No
EHMT1	c.110G>A	p.Gly37Asp	CADD (21.3), SIFT (T), PolyPhen (B), M-CAP (D), REVEL (0.1)	0506	4y6m	male	DEE	Mild	No
EPB41L1	c.1479G>C	p.Lys493Asn	CADD (24.8), SIFT (D), PolyPhen (P), M-CAP (D), REVEL (0.3)	0438	8m	male	DEE	Moderate	No
FBXO11	c.173C>T	p.Pro58Leu	CADD (22), SIFT (T), PolyPhen (B), M-CAP (D), REVEL (0.3)	0968	5m	male	GGE	Mild	No
FGFR1	c.1486C>T	p.Leu496Phe	CADD (28.4), SIFT (D), PolyPhen (D), M-CAP (D), REVEL (0.9)	0919	10y	female	IGE	N	No
GABBR2	c.2737A>G	p.Ser913Gly	CADD (24.2), SIFT (D), PolyPhen (B), M-CAP (D), REVEL (0.3)	0731	5y	female	NAFE	Mild	No
GABRB2	c.577C>T	p.Arg193Cys	CADD (25.5), SIFT (D), PolyPhen (B), M-CAP (D), REVEL (0.7)	0525	5y	male	NAFE	N	No
GABRB2	c.1372C>G	p.Arg458Gly	CADD (26.8), SIFT (D), PolyPhen (D), M-CAP (D), REVEL (0.5)	0465	4y	female	Combined	Borderline	No
GABRB3	c.296A>G	p.Tyr99Cys	CADD (29.4), SIFT (D), PolyPhen (D), M-CAP (D), REVEL (0.9)	0909	1y6m	female	DEE	Moderate	Yes
GABRB3	c.428T>G	p.Leu143Arg	CADD (28.7), SIFT (D), PolyPhen (D), M-CAP (D), REVEL (0.9)	0536	2y	male	GGE	N	No
GFAP	c.836A>G	p.Lys279Arg	CADD (26.2), SIFT (T), PolyPhen (P), M-CAP (D), REVEL (0.8)	1030	3m	female	DEE	N	No
GLI3	c.440A>G	p.His147Arg	CADD (23.6), SIFT (T), PolyPhen (P), M-CAP (D), REVEL (0.4)	0542	4y	female	EE	Borderline	No
GLI3	c.4595C>T	p.Ser1532Phe	CADD (29.2), SIFT (D), PolyPhen (D), M-CAP (D), REVEL (0.5)	0962	12y	male	GGE	N	No
GLUD1	c.709G>T	p.Ala237Ser	CADD (27.8), SIFT (D), PolyPhen (P), M-CAP (D), REVEL (0.8)	0660	5y	male	NAFE	N	No
GPHN	c.2114C>T	p.Pro705Leu	CADD (25.9), SIFT (D), PolyPhen (D), M-CAP (T), REVEL (0.2)	0920	1y	male	GGE	Mild	Yes
GPHN	c.623C>T	p.Pro208Leu	CADD (25), SIFT (D), PolyPhen (D), M-CAP (D), REVEL (0.4)	0875	4y	female	NAFE	N	No

© 2023 Koh HY et al. *JAMA Network Open.*

GRIA4	c.1018G>C	p.Gly340Arg	CADD (22.6), SIFT (T), PolyPhen (B), M-CAP (D), REVEL (0.3)	0669	3y	male	GGE	Borderline	No
GRIN2A	c.1663G>A	p.Ala555Thr	CADD (25.9), SIFT (D), PolyPhen (D), M-CAP (D), REVEL (0.2)	0592	4y	female	NAFE	N	No
GRIN2D	c.2800C>T	p.Pro934Ser	CADD (23.5), SIFT (D), PolyPhen (P), M-CAP (D), REVEL (0.1)	0426	13y	female	DEE	Mild	No
HMBS	c.61A>T	p.Ile21Phe	CADD (24.6), SIFT (T), PolyPhen (P), M-CAP (D), REVEL (0.9)	0587	8y	female	Combined	N	No
IFIH1	c.2891G>T	p.Cys964Phe	CADD (26.2), SIFT (D), PolyPhen (D), M-CAP (D), REVEL (0.8)	0523	7m	male	DEE	Severe	No
IFIH1	c.77T>A	p.Ile26Asn	CADD (31), SIFT (D), PolyPhen (P), M-CAP (D), REVEL (0.4)	0844	3y	female	NAFE	N	No
KAT6A	c.4058A>G	p.Asp1353Gly	CADD (23.4), SIFT (T), PolyPhen (P), M-CAP (D), REVEL (0.2)	0791	3y	female	DEE	Mild	No
KAT6B	c.2992T>A	p.Ser998Thr	CADD (25), SIFT (D), PolyPhen (D), M-CAP (D), REVEL (0.3)	0920	1y	male	GGE	Mild	Yes
KAT6B	c.786A>T	p.Leu262Phe	CADD (25), SIFT (D), PolyPhen (D), M-CAP (D), REVEL (0.5)	0503	5m	female	DEE	Severe	No
KCNA1	c.1126G>A	p.Gly376Ser	CADD (29.2), SIFT (D), PolyPhen (D), M-CAP (D), REVEL (0.97)	0638	4y	male	NAFE	N	No
KCNA1	c.1217T>A	p.Val406Asp	CADD (29.5), SIFT (D), PolyPhen (D), M-CAP (D), REVEL (1)	1043	11m	male	DEE	Severe	Yes
KCNH2	c.1979C>T	p.Ser660Leu	CADD (32), SIFT (D), PolyPhen (D), M-CAP (D), REVEL (0.9)	0627	2y6m	male	NAFE	Borderline	No
KCNH2	c.788C>G	p.Ser263Cys	CADD (23.4), SIFT (D), PolyPhen (B), M-CAP (D), REVEL (0.6)	0701	14y	female	GGE	N	No
KCNT1	c.2131G>A	p.Ala711Thr	CADD (24.0), SIFT (D), PolyPhen (D), M-CAP (T), REVEL (0.2)	0713	1y5m	male	DEE	Mild	Yes
KCNQ2	c.200C>G	p.Pro67Arg	CADD (26.3), SIFT (), PolyPhen (), M-CAP (D), REVEL (0.7)	1050	6m	male	DEE	Severe	No
KCNQ5	c.767G>C	p.Gly256Ala	CADD (26.3), SIFT (D), PolyPhen (P), M-CAP (D), REVEL (0.8)	0566	2m	male	DEE	Moderate	No
KDM4B	c.2360G>A	p.Arg787Gln	CADD (27.8), SIFT (D), PolyPhen (D), M-CAP (D), REVEL (0.8)	0710	8y	female	GGE	N	No
KDM4B	c.2755G>A	p.Val919Met	CADD (23.5), SIFT (T), PolyPhen (B), M-CAP (T), REVEL (0.1)	0475	2y	male	DEE	Moderate	No
KIF5A	c.2695C>T	p.Arg899Cys	CADD (31), SIFT (D), PolyPhen (D), M-CAP (D), REVEL (0.7)	0548	7y6m	male	Combined	Borderline	No
KMT2A	c.10700C>T	p.Ser3567Phe	CADD (24.5), SIFT (T), PolyPhen (D), M-CAP (D), REVEL (0.3)	1006	14y	male	GGE	Borderline	No
KMT2A	c.2614A>C	p.Lys872Gln	CADD (25.1), SIFT (D), PolyPhen (P), M-CAP (T), REVEL (0.2)	0551	1y6m	female	DEE	Mild	No
KMT2A	c.8065A>G	p.Arg2689Gly	CADD (24.7), SIFT (D), PolyPhen (D), M-CAP (D), REVEL (0.8)	0531	5y	female	NAFE	Mild	No
KMT2A	c.8149A>G	p.Ile2717Val	CADD (24.9), SIFT (D), PolyPhen (D), M-CAP (D), REVEL (0.5)	1050	6m	male	DEE	Severe	No
KMT2A	c.9137A>C	p.Asn3046Thr	CADD (22.8), SIFT (D), PolyPhen (B), M-CAP (D), REVEL (0.3)	0636	1y3m	female	DEE	Moderate	No
KMT2D	c.10471C>T	p.Arg3491Cys	CADD (25.2), SIFT (D), PolyPhen (B), M-CAP (D), REVEL (0.4)	0889	4y	female	GGE	N	No
KMT2D	c.3077C>T	p.Pro1026Leu	CADD (21.8), SIFT (D), PolyPhen (B), M-CAP (D), REVEL (0.2)	0605	2y	female	NAFE	N	No
KMT2D	c.5899G>A	p.Gly1967Ser	CADD (23.1), SIFT (T), PolyPhen (P), M-CAP (D), REVEL (0.4)	0715	6y	female	GGE	N	No
KMT2D	c.9815C>T	p.Pro3272Leu	CADD (22), SIFT (T), PolyPhen (B), M-CAP (D), REVEL (0.4)	1063	9y	female	GGE	N	No
KMT2E	c.3955A>C	p.Ser1319Arg	CADD (22.2), SIFT (T), PolyPhen (B), M-CAP (D), REVEL (0.2)	0933	8y	female	NAFE	N	No
KMT2E	c.4879C>T	p.Pro1627Ser	CADD (22.8), SIFT (D), PolyPhen (D), M-CAP (D), REVEL (0.4)	0779	1y1m	male	Combined	N	No
MACF1	c.14124C>A	p.Phe4708Leu	CADD (27.8), SIFT (D), PolyPhen (D), M-CAP (D), REVEL (0.6)	0845	12y	female	Combined	N	No
MACF1	c.16030C>T	p.Arg5344Trp	CADD (26), SIFT (T), PolyPhen (D), M-CAP (D), REVEL (0.2)	0962	12y	male	GGE	N	No
MACF1	c.4171C>T	p.Arg1391Cys	CADD (31), SIFT (D), PolyPhen (D), M-CAP (D), REVEL (0.4)	0829	infantile	male	DEE	Moderate	Yes
MAF	c.343G>C	p.Glu115Gln	CADD (26.4), SIFT (D), PolyPhen (D), M-CAP (D), REVEL (0.6)	0592	4y	female	NAFE	N	No
MAST1	c.4180G>T	p.Val1394Leu	CADD (23.2), SIFT (D), PolyPhen (B), M-CAP (D), REVEL (0.1)	0592	4y	female	NAFE	N	No
MED13L	c.2054A>G	p.Asp685Gly	CADD (24), SIFT (D), PolyPhen (B), M-CAP (T), REVEL (0.1)	0876	2y6m	male	GGE	N	No
MYH3	c.2147A>G	p.Tyr716Cys	CADD (31), SIFT (D), PolyPhen (D), M-CAP (D), REVEL (0.9)	0989	5y	female	IGE	N	No
MYT1L	c.1810G>A	p.Val604Met	CADD (25), SIFT (D), PolyPhen (D), M-CAP (D), REVEL (0.1)	0543	12y	male	GGE	N	No

© 2023 Koh HY et al. *JAMA Network Open.*

NBEA	c.197C>T	p.Pro66Leu	CADD (28.9), SIFT (D), PolyPhen (D), M-CAP (D), REVEL (0.4)	0974	9y	male	GGE	N	No
NEDD4L	c.1682A>G	p.Asp561Gly	CADD (33), SIFT (D), PolyPhen (D), M-CAP (D), REVEL (0.9)	0522	10y	male	IGE	Borderline	Yes
NEDD4L	c.1708A>T	p.Asn570Tyr	CADD (32), SIFT (D), PolyPhen (D), M-CAP (D), REVEL (0.9)	0923	5y	female	IGE	N	No
NF1	c.241C>A	p.Leu81Ile	CADD (23), SIFT (D), PolyPhen (B), M-CAP (D), REVEL (0.1)	0923	5y	female	IGE	N	No
NF1	c.373C>T	p.Arg125Cys	CADD (24), SIFT (T), PolyPhen (P), M-CAP (D), REVEL (0.5)	0866	4y	female	NAFE	N	No
NOTCH3	c.2981G>T	p.Gly994Val	CADD (25), SIFT (D), PolyPhen (D), M-CAP (D), REVEL (0.8)	1118	7y	female	NAFE	N	No
NOTCH3	c.739A>C	p.Asn247His	CADD (25.5), SIFT (D), PolyPhen (D), M-CAP (D), REVEL (0.9)	0912	8m	female	Combined	Borderline	No
NRXN1	c.1970G>T	p.Gly657Val	CADD (25.9), SIFT (D), PolyPhen (D), M-CAP (D), REVEL (0.9)	0730	5y	male	GGE	N	No
NRXN1	c.2293A>G	p.Met765Val	CADD (25.1), SIFT (D), PolyPhen (P), M-CAP (D), REVEL (0.5)	0869	6m	male	DEE	Borderline	No
NRXN1	c.2558G>C	p.Arg853Pro	CADD (27.6), SIFT (D), PolyPhen (D), M-CAP (D), REVEL (0.8)	0763	6m	male	DEE	Borderline	No
NSD1	c.1567G>T	p.Asp523Tyr	CADD (22.7), SIFT (D), PolyPhen (B), M-CAP (D), REVEL (0.3)	0600	2y	female	Combined	N	No
NSD1	c.3500G>T	p.Arg1167Leu	CADD (26.4), SIFT (D), PolyPhen (P), M-CAP (D), REVEL (0.4)	0865	1y4m	female	DEE	Borderline	Yes
OPA1	c.1933C>G	p.Arg645Gly	CADD (26.3), SIFT (D), PolyPhen (D), M-CAP (D), REVEL (0.9)	0650	3y	female	GGE	N	No
PDCD10	c.148A>C	p.Lys50Gln	CADD (23.6), SIFT (T), PolyPhen (B), M-CAP (D), REVEL (0.1)	0712	7y	male	NAFE	N	No
PHOX2B	c.137C>T	p.Pro46Leu	CADD (28.4), SIFT (D), PolyPhen (P), M-CAP (D), REVEL (0.8)	1114	12y	male	DEE	Severe	Yes
PPP3CA	c.1194C>A	p.Asn398Lys	CADD (23.1), SIFT (D), PolyPhen (B), M-CAP (T), REVEL (0.1)	0559	5y	female	IGE	N	Yes
PSEN1	c.752C>T	p.Ala251Val	CADD (24.6), SIFT (T), PolyPhen (D), M-CAP (D), REVEL (0.7)	0885	16y	female	GGE	N	No
PTCH1	c.3926A>G	p.Glu1309Gly	CADD (24.4), SIFT (D), PolyPhen (B), M-CAP (D), REVEL (0.5)	0823	2y	female	NAFE	N	No
PTCH1	c.655A>G	p.Ile219Val	CADD (21.3), SIFT (T), PolyPhen (B), M-CAP (D), REVEL (0.4)	0813	2y	female	Combined	N	No
PUM1	c.731A>G	p.Asp244Gly	CADD (24.9), SIFT (T), PolyPhen (B), M-CAP (D), REVEL (0.2)	1099	3y	female	GGE	Mild	No
RAI1	c.5546T>G	p.Met1849Arg	CADD (27.4), SIFT (D), PolyPhen (P), M-CAP (D), REVEL (0.7)	0856	2y	female	GGE	N	No
RERE	c.2161A>G	p.Asn721Asp	CADD (26.5), SIFT (D), PolyPhen (D), M-CAP (D), REVEL (0.2)	0633	6y	male	NAFE	Mild	No
RERE	c.3092A>C	p.Gln1031Pro	CADD (23.9), SIFT (D), PolyPhen (P), M-CAP (D), REVEL (0.2)	0665	8y	female	IGE	N	No
RYR2	c.2603A>G	p.Asp868Gly	CADD (31), SIFT (D), PolyPhen (D), M-CAP (D), REVEL (1)	0952	infantile onset	female	DEE	N	No
RYR2	c.6742A>G	p.Met2248Val	CADD (23.9), SIFT (D), PolyPhen (B), M-CAP (D), REVEL (0.8)	0930	7y	male	Combined	N	No
RYR2	c.8768A>G	p.Tyr2923Cys	CADD (24.2), SIFT (D), PolyPhen (B), M-CAP (D), REVEL (0.7)	1018	3m	male	DEE	Severe	Yes
SCN1A	c.1850G>A	p.Arg617Lys	CADD (23.9), SIFT (T), PolyPhen (P), M-CAP (D), REVEL (0.8)	0527	5y	female	GGE	N	No
SCN3A	c.5147C>T	p.Ala1716Val	CADD (23.7), SIFT (T), PolyPhen (P), M-CAP (D), REVEL (0.7)	0462	12y	male	Combined	N	No
SCN8A	c.4715A>C	p.Lys1572Thr	CADD (28.5), SIFT (D), PolyPhen (D), M-CAP (D), REVEL (0.9)	0438	8m	male	DEE	Moderate	No
SETD1A	c.224C>T	p.Ser75Phe	CADD (24), SIFT (T), PolyPhen (D), M-CAP (T), REVEL (0.2)	1027	4y	female	DEE	Mild	No
SETD1A	c.4594G>T	p.Val1532Leu	CADD (23.8), SIFT (T), PolyPhen (P), M-CAP (D), REVEL (0.5)	0699	11y	male	NAFE	Moderate	Yes
SLC6A1	c.650C>T	p.Thr217Met	CADD (25.5), SIFT (D), PolyPhen (D), M-CAP (D), REVEL (0.7)	0629	3y	female	Combined	N	No
SMARCA2	c.2915C>T	p.Ala972Val	CADD (32), SIFT (D), PolyPhen (D), M-CAP (D), REVEL (0.7)	0862	1y6m	male	DEE	Mild	No
SMARCC2	c.747T>A	p.Asn249Lys	CADD (23.3), SIFT (D), PolyPhen (D), M-CAP (D), REVEL (0.4)	1010	1w	female	NAFE	N	No
SMC3	c.556C>T	p.Arg186Trp	CADD (24.7), SIFT (T), PolyPhen (D), M-CAP (D), REVEL (0.6)	0815	2y	female	GGE	N	No
SON	c.2038G>A	p.Ala680Thr	CADD (25.6), SIFT (D), PolyPhen (D), M-CAP (T), REVEL (0.2)	0792	2y	female	NAFE	N	No
SON	c.2188A>G	p.Thr730Ala	CADD (22.8), SIFT (D), PolyPhen (P), M-CAP (T), REVEL (0.1)	0433	6y	female	IGE	N	No
SON	c.2878C>G	p.Pro960Ala	CADD (23.7), SIFT (D), PolyPhen (D), M-CAP (T), REVEL (0.2)	0499	2y6m	female	Combined	Borderline	Yes

© 2023 Koh HY et al. *JAMA Network Open.*

SON	c.5968C>T	p.Arg1990Cys	CADD (24.8), SIFT (D), PolyPhen (D), M-CAP (D), REVEL (0.3)	0431	9m	male	DEE	Severe	No
SRCAP	c.1361C>T	p.Ala454Val	CADD (25.8), SIFT (D), PolyPhen (D), M-CAP (D), REVEL (0.6)	0902	3y	female	IGE	Borderline	No
SRCAP	c.6056G>A	p.Arg2019His	CADD (22.9), SIFT (T), PolyPhen (B), M-CAP (T), REVEL (0.4)	0902	3y	female	IGE	Borderline	No
SRCAP	c.8513G>T	p.Gly2838Val	CADD (20.9), SIFT (D), PolyPhen (B), M-CAP (D), REVEL (0.3)	0548	7y6m	male	Combined	Borderline	No
SRCAP	c.9368G>A	p.Arg3123His	CADD (27.7), SIFT (D), PolyPhen (D), M-CAP (D), REVEL (0.4)	0527	5y	female	GGE	N	No
STX16	c.53T>C	p.Ile18Thr	CADD (24.3), SIFT (D), PolyPhen (B), M-CAP (T), REVEL (0.2)	0981	1y	male	DEE	Mild	Yes
SYNGAP1	c.2435C>T	p.Pro812Leu	CADD (23.4), SIFT (T), PolyPhen (P), M-CAP (T), REVEL (0.2)	0568	16y	male	GGE	N	No
TANC2	c.2189G>T	p.Gly730Val	CADD (25.8), SIFT (D), PolyPhen (P), M-CAP (D), REVEL (0.7)	0454	16y	male	GGE	Moderate	Yes
TANC2	c.3761T>A	p.Phe1254Tyr	CADD (27.7), SIFT (D), PolyPhen (P), M-CAP (D), REVEL (0.3)	1098	2y	male	GGE	Borderline	No
TBX1	c.391G>A	p.Glu131Lys	CADD (25.3), SIFT (T), PolyPhen (P), M-CAP (D), REVEL (0.6)	0539	9y	male	NAFE	N	No
TCF4	c.1052C>T	p.Pro351Leu	CADD (25.9), SIFT (T), PolyPhen (D), M-CAP (T), REVEL (0.3)	0916	1y3m	male	NAFE	N	No
TGFBR2	c.1546G>A	p.Val516Met	CADD (31), SIFT (D), PolyPhen (D), M-CAP (D), REVEL (0.6)	0594	9y	male	NAFE	N	No
TGFBR2	c.1621A>T	p.Thr541Ser	CADD (23.6), SIFT (T), PolyPhen (P), M-CAP (T), REVEL (0.3)	0596	2y6m	male	DEE	Moderate	Yes
TRAF7	c.1829C>T	p.Thr610Met	CADD (26.4), SIFT (D), PolyPhen (D), M-CAP (D), REVEL (0.3)	0680	7m	male	NAFE	Borderline	Yes
TRIO	c.2632G>T	p.Val878Phe	CADD (28), SIFT (D), PolyPhen (D), M-CAP (D), REVEL (0.4)	0010	5y	male	IGE	Mild	No
TRIO	c.7543C>G	p.Arg2515Gly	CADD (24.2), SIFT (D), PolyPhen (D), M-CAP (D), REVEL (0.1)	0578	9y	female	NAFE	N	No
TRIO	c.7853T>A	p.Ile2618Asn	CADD (25.9), SIFT (D), PolyPhen (P), M-CAP (T), REVEL (0.2)	0559	5y	female	IGE	N	Yes
TRIO	c.8513C>T	p.Thr2838Ile	CADD (24.1), SIFT (T), PolyPhen (D), M-CAP (T), REVEL (0.3)	0791	3y	female	DEE	Mild	No
TRRAP	c.7478T>C	p.Met2493Thr	CADD (25.9), SIFT (D), PolyPhen (P), M-CAP (T), REVEL (0.4)	1081	14y	female	GGE	N	No
TRRAP	c.842T>C	p.Ile281Thr	CADD (24.3), SIFT (T), PolyPhen (P), M-CAP (T), REVEL (0.4)	1105	2.5y	male	NAFE	Borderline	No
TRRAP	c.8644A>G	p.Ser2882Gly	CADD (22.7), SIFT (T), PolyPhen (B), M-CAP (T), REVEL (0.4)	0509	5m	male	DEE	Severe	No
TRRAP	c.9196G>A	p.Val3066Ile	CADD (27.1), SIFT (D), PolyPhen (P), M-CAP (T), REVEL (0.3)	0632	3y	female	GGE	N	No
TSC1	c.2707G>C	p.Asp903His	CADD (26.1), SIFT (D), PolyPhen (D), M-CAP (D), REVEL (0.5)	0730	5y	male	GGE	N	No
TSC2	c.1694T>C	p.Leu565Pro	CADD (25.6), SIFT (T), PolyPhen (D), M-CAP (D), REVEL (0.6)	1092	4y	male	GGE	N	No
UBE3A	c.2411T>A	p.Leu804His	CADD (25.9), SIFT (T), PolyPhen (D), M-CAP (D), REVEL (0.4)	0710	8y	female	GGE	N	No
ZBTB18	c.674G>A	p.Cys225Tyr	CADD (23.8), SIFT (D), PolyPhen (B), M-CAP (T), REVEL (0.1)	0871	2y	male	Combined	N	No
ZEB2	c.26G>A	p.Gly9Asp	CADD (26.3), SIFT (D), PolyPhen (D), M-CAP (D), REVEL (0.7)	1067	11y	male	NAFE	N	No
ZEB2	c.272G>A	p.Gly91Asp	CADD (21.3), SIFT (T), PolyPhen (B), M-CAP (T), REVEL (0.2)	0904	1y6m	female	GGE	Moderate	No

Cryptic splicing variants

Gene	DNA	Protein	Consequence*In silico* prediction	Cases	Seizure onset	Sex	Epilepsy	ID	ASD
ATP1A3	c.1669+5G>T		Splice donor loss at +5	0775	8y	female	GGE	N	No
CHD8	c.1969-9_1969-6delTTTT		Splice acceptor loss at -6	0454	16y	male	GGE	Moderate	Yes
COL4A1	c.858+4A>T		Splice donor loss at +4	0631	5m	female	DEE	Moderate	No
CYFIP2	c.2660+7G>T		Splice donor gain at -2	0661	4y	female	NAFE	Borderline	No
KIF11	c.2922+7T>G		Splice donor gain at -1	0875	4y	female	NAFE	N	No
KMT2D	c.5643A>G		Splice donor gain at -1	0639	4m	female	GGE	N	No
SETD5	c.567+3A>T		Splice donor gain at +6	0879	1w	female	NAFE	N	No

© 2023 Koh HY et al. *JAMA Network Open.*

SHANK3	c.289-5T>A		Splice acceptor gain at +2	1071	3y6m	female	DEE	Moderate	Yes
SNAP25	c.407+5G>A		Splice donor loss at -5	0509	5m	male	DEE	Severe	No
TBX1	c.54C>G	p.Asp18Glu	Splice acceptor loss at +8	0769	1y5m	male	NAFE	Moderate	No

In-frame indels

**Gene**	**DNA**	**Protein**	**Consequence*In silico* prediction**	**Cases**	**Seizure onset**	**Sex**	**Epilepsy**	**ID**	**ASD**
ARID1A	c.375_380dupTGGCGG	p.Gly126_Gly127dup	Inframe deletion	0810	2y	male	GGE	N	No
CACNA1A			Inframe deletion	0951	11y	female	NAFE	N	No
CHRNA2	c.887_889delAGA	p.Lys296del	Inframe deletion	0699	11y	male	NAFE	Moderate	Yes
DNMT1			Inframe deletion	0981	1y	male	DEE	Mild	Yes
EHMT1			Inframe deletion	1114	12y	male	DEE	Severe	Yes
EZH2	c.1156_1161delAAAGAA	p.Lys386_Glu387del	Inframe deletion	0580	2y	male	GGE	N	No
FOXG1	c.170_172dupACC	p.His57dup	Inframe deletion	0724	2y11m	male	GGE	N	No
KANSL1			Inframe deletion	0933	8y	female	NAFE	N	No
SETD1B			Inframe deletion	1054	4y	male	IGE	Mild	No
SMARCA2			Inframe deletion	0811	2y	male	Combined	Moderate	No
SMARCA2	c.685_686insCGCAGCAGC	p.Gln228_Gln229insProGlnGln	Inframe deletion	0609	5y	female	IGE	N	No
SKI	c.249_251dupGCC	p.Pro84dup	Inframe deletion	0841	6y	male	NAFE	N	No
TANC2	c.4125_4133dupGCAGCAGCC	p.Gln1379_PRO1381dup	Inframe deletion	0462	12y	male	Combined	N	No
TBX1	c.55_63delAGCAGCCTG	p.Ser19_Leu21del	Inframe deletion	0519	5y	female	IGE	N	No
ZSWIM6			Inframe deletion	0691	2w	male	DEE	Severe	No

VUS, variant(s) of uncertain significance; ID, intellectual disability; ASD, autism spectrum disorder; DEE, developmental and epileptic encephalopathies; GGE, genetic generalized epilepsy; IGE, idiopathic generalized epilepsy; NAFE, non-acquired focal epilepsy; Y, yes; N, none; CADD, combined annotation dependent depletion; SIFT, sorting intolerant from tolerant; M-CAP, Mendelian clinically applicable pathogenicity; REVEL, rare exome variant ensemble learner; D, damaging; T; tolerable; B, benign; P, possibly damaging.

© 2023 Koh HY et al. *JAMA Network Open.*

eTable 4. Novel Candidate Epilepsy Genes and Their Associated Phenotypes

Epilepsy	Cases	Seizure onset	Sex	ID	ASD	Syndrome	Gene	Expressed in brain	Variant	Inheritance	Zygosity	AF	*In silico* prediction
DEE	0763	6m	male	Borderline	N	IESS	*ANKRD27*	Y	c.2942_2943del p.Leu981GlnfsTer4	both	homo	0.000	LoF
	0814	2y	male	Moderate	Y		*ANKRD34B*	Y	c.1454C>T p.Pro485Leu	*de novo*	hetero	N/A	CADD (25), SIFT (D), PolyPhen (D), M-CAP (D), REVEL (0.5)
	0659	7m	female	Mild	Y	IESS	*CA5A*	Y	c.287G>C p.Trp96Ser	both	homo	0.000	CADD (25), SIFT (T), PolyPhen (D), M-CAP (D), REVEL (0.4)
	0565	1w	male	Profound	N		*CELSR3*	Y	c.4352C>T p.Ala1451Val	unknown	homo	0.000	CADD (22), SIFT (T), PolyPhen (B), M-CAP (D), REVEL (0.3)
	0662	3m	female	Severe	N		*CLDN5*	Y	c.360C>G p.Phe120Leu	*de novo*	hetero	N/A	CADD (30), SIFT (D), PolyPhen (D), M-CAP (D), REVEL (0.8)
	0981	1y	male	Mild	Y		*CTXN1*	Y	c.247T>A p.Ter83ArgextTer109	*de novo*	hetero	N/A	-
	0825	1y10m	male	Borderline	N	IESS	*FZD5*	Y	c.1024C>A p.Leu342Met	*de novo*	hetero	N/A	CADD (21), SIFT (D), PolyPhen (D), M-CAP (D), REVEL (0.6)
	0747	4m	female	Profound	N	IESS	*GNAZ*	Y	c.49C>T p.Arg17Trp	*de novo*	hetero	0.000	CADD (24), SIFT (D), PolyPhen (B), M-CAP (D), REVEL (0.6)
	0747	4m	female	Profound	N	IESS	*GRID2IP*	Y	c.1269-2A>G	*de novo*	hetero	N/A	LoF
	0686	4m	male	Severe	Y	IESS	*HTR3A*	Y	c.347T>A p.Ile116Asn	paternal	hetero	0.000	CADD (28), SIFT (D), PolyPhen (D), M-CAP (D), REVEL (0.8)
							HTR3A	Y	c.932T>C p.Ile311Thr	unknown	hetero	0.000	CADD (25), SIFT (D), PolyPhen (D), M-CAP (D), REVEL (0.9)
	0435	7y	female	Borderline	Y	LGS	*LYL1*	Y	c.529_558del p.Leu177_Leu186del	*de novo*	hetero	N/A	Conserved region
	1050	6m	male	Severe	N		*MICALL2*	Y	c.599G>A p.Arg200Gln	both	homo	0.000	CADD (28), SIFT (D), PolyPhen (D), M-CAP (D), REVEL (0.7)
	0723	5y	male	Moderate	Y		*MORC4*	Y	c.307A>C p.Ser103Arg	unknown	hemi	N/A	CADD (32), SIFT (D), PolyPhen (D), M-CAP (D), REVEL (0.9)
	0429	7m	male	Moderate	Y	IESS, LGS	*NCOR1*	Y	c.1765G>A p.Gly589Arg	*de novo*	hetero	N/A	CADD (32), SIFT (D), PolyPhen (D), M-CAP (D), REVEL (0.4)
	0770	4m	female	Moderate	N	IESS	*NLRX1*	Y	c.1066C>T p.Arg356Trp	both	homo	0.000	CADD (26), SIFT (D), PolyPhen (P), M-CAP (D), REVEL (0.4)
	0898	3m	female	Moderate	N	IESS, LGS	*PPARD*	Y	c.424G>T p.Ala142Ser	*de novo*	hetero	N/A	CADD (34), SIFT (D), PolyPhen (B), M-CAP (D), REVEL (0.7)
	0542	4y	female	Borderline	N	EE-SWAS	*ROBO1*	Y	c.595delG p.Glu199SerfsTer20	*de novo*	hetero	N/A	LoF
	0561	7y	male	Moderate	Y	LGS	*TCF12*	Y	c.82del p.Ser28ProfsTer59	*de novo*	hetero	N/A	LoF

© 2023 Koh HY et al. *JAMA Network Open.*

	0896	2y	female	Mild	N	LGS	*TMEM57*	Y	c.988A>C p.Asn330His	*de novo*	hetero	N/A	CADD (24), SIFT (T), PolyPhen (P), M-CAP (T), REVEL (0.2)
	0747	4m	female	Profound	N	IESS	*TRPC7*	Y	c.1229T>G p.Phe410Cys	*de novo*	hetero	N/A	CADD (27), SIFT (D), PolyPhen (D), M-CAP (D), REVEL (0.7)
	1089	1y6m	male	Moderate	Y		*TRPM7*	Y	c.3137G>A p.Gly1046Asp	*de novo*	hetero	N/A	CADD (26), SIFT (D), PolyPhen (D), M-CAP (D), REVEL (1)
	0794	4m	female	Severe	N	IESS, LGS	*TWIST1*	Y	c.68A>G p.Glu23Gly	*de novo*	hetero	N/A	CADD (24), SIFT (D), PolyPhen (B), M-CAP (D), REVEL (0.4)
	0691	2w	male	Severe	N	IESS	*ZNF729*	Y	c.2957A>T p.His986Leu	unknown	homo	N/A	CADD (21), SIFT (D), PolyPhen (D), M-CAP (T), REVEL (0.3)
	0911	8y	male	Mild	N	FIRES	*ZNF879*	Y	c.59C>T p.Ala20Val	*de novo*	hetero	N/A	CADD (22), SIFT (T), PolyPhen (B), M-CAP (T), REVEL (0.2)
GGE	0812	2y	male	N/A	N		*ACAD9*	Y	c.359del p.Phe120SerfsTer9	maternal	hetero	0.000	LoF
									c.1684G>A p.Asp562Asn	paternal	hetero	0.000	CADD (24), SIFT (T), PolyPhen (D), M-CAP (D), REVEL (0.4)
	0568	16y	male	N/A	N		*AGBL2*	Y	c.725C>A p.Ser242Tyr	*de novo*	hetero	N/A	CADD (28), SIFT (D), PolyPhen (D), M-CAP (D), REVEL (0.4)
	0568	16y	male	N/A	N		*ATXN2*	Y	c.950C>G p.Ser317Trp	*de novo*	hetero	N/A	CADD (25), SIFT (D), PolyPhen (P), M-CAP (D), REVEL (0.2)
	0472	1y2m	female	N/A	N	MEI	*BRINP1*	Y	c.1565_1570del p.Lys522_Arg523del	*de novo*	hetero	N/A	Conserved region
	0891	9y	male	N/A	Y		*BIRC6*	Y	c.14086C>A p.Gln4696Lys	*de novo*	hetero	N/A	-
	0521	5y2m	male	N/A	N		*CAMSAP3*	Y	c.3507del p.Lys1170ArgfsTer17	*de novo*	hetero	N/A	LoF
	0968	5m	male	Mild	N		*CDH2*	Y	c.1161T>G p.Phe387Leu	*de novo*	hetero	N/A	CADD (25), SIFT (D), PolyPhen (B), M-CAP (D), REVEL (0.5)
	0748	12y	male	Moderate	Y		*DDX47*	Y	c.334_342dup p.Phe112_Ala114dup	*de novo*	hetero	N/A	Conserved region
	0819	6y	female	N/A	N		*FBXL18*	Y	c.1946dup p.Thr650HisfsTer89	*de novo*	hetero	N/A	LoF
	0724	2y11m	male	N/A	N		*FBXO44*	Y	c.146del p.Arg49ProfsTer50	paternal (mosaic)	hetero	N/A	LoF
	0962	12y	male	N/A	N		*GLI3*	Y	c.4595C>T p.Ser1532Phe	*de novo*	hetero	N/A	CADD (29), SIFT (D), PolyPhen (D), M-CAP (D), REVEL (0.5)
	1092	4y	male	N/A	N	FIRES	*GLRA4*	Y	c.384G>C p.Trp128Cys	maternal	hemi	N/A	-
	0876	2y6m	male	N/A	N		*HDAC3*	Y	c.601C>T p.Pro201Ser	*de novo*	hetero	N/A	-
	1003	4y	male	Mild	N		*KHSRP*	Y	c.1138G>A p.Glu380Lys	*de novo*	hetero	N/A	CADD (25), SIFT (D), PolyPhen (B), M-CAP (D), REVEL (0.1)
	0560	3y	female	N/A	N	EEM	*LMTK3*	Y	c.655C>T p.His219Tyr	*de novo*	hetero	N/A	CADD (26), SIFT (D), PolyPhen (D), M-CAP (D), REVEL (0.8)

© 2023 Koh HY et al. *JAMA Network Open.*

	0626	1y6m	female	N/A	N		*MAOB*	Y	c.206T>G p.Leu69Trp	*de novo*	hetero	N/A	CADD (26), SIFT (D), PolyPhen (D), M-CAP (D), REVEL (0.9)
	0748	12y	male	Moderate	Y		*NXT1*	Y	c.376_377del p.Trp126GlufsTer5	*de novo*	hetero	N/A	LoF
	0762	1y	female	N/A	N	EEM	*PAM*	Y	c.1790A>G p.Tyr597Cys	*de novo*	hetero	N/A	CADD (27), SIFT (D), PolyPhen (D), M-CAP (D), REVEL (0.7)
	1093	15y	male	N/A	N		*PLXNB3*	Y	c.4592G>C p.Arg1531Pro	maternal	hemi	N/A	CADD (29), SIFT (D), PolyPhen (D), M-CAP (D), REVEL (0.6)
	0454	16y	male	Moderate	Y		*PRPS1*	Y	c.590A>C p.Lys197Thr	maternal	hemi	N/A	CADD (25), SIFT (T), PolyPhen (P), M-CAP (D), REVEL (0.8)
	0493	4y	female	Borderline	N		*SEMA4B*	Y	c.499A>G p.Thr167Ala	*de novo*	hetero	N/A	CADD (24), SIFT (T), PolyPhen (B), M-CAP (T), REVEL (0.1)
	0867	11y	female	N/A	N		*SLC12A4*	Y	c.2026C>T p.Arg676Cys	both	homo	0.000	CADD (26), SIFT (D), PolyPhen (D), M-CAP (D), REVEL (0.9)
	1074	6y	male	N/A	N		*TCEAL3*	Y	c.536G>A p.Arg179Gln	maternal	hemi	N/A	CADD (24), SIFT (D), PolyPhen (P), M-CAP (T), REVEL (0.1)
	1001	6y	male	N/A	N		*USP51*	Y	c.1472G>A p.Gly491Asp	maternal	hemi	N/A	CADD (23), SIFT (T), PolyPhen (D), M-CAP (T), REVEL (0.2)
	0706	2y	male	Borderline	N		*ZDHHC15*	Y	c.450-6T>G	maternal	hemi	N/A	Acceptor loss at -6
	0812	2y	male	N/A	N		*ZNF488*	Y	c.945G>T p.Glu315Asp	*de novo*	hetero	N/A	-
IGE	0722	13y	female	N/A	N	JME	*ARIH2*	Y	c.475C>T p.Gln159Ter	*de novo*	hetero	N/A	LoF
	0989	5y	female	N/A	N	CAE	*INO80*	Y	c.679A>G p.Lys227Glu	*de novo*	hetero	0.000	CADD (23), SIFT (T), PolyPhen (B), M-CAP (D), REVEL (0.4)
	0579	6y	female	N/A	N	CAE	*KIAA1211*	Y	c.2576A>G p.Asn859Ser	*de novo*	hetero	N/A	CADD (23), SIFT (D), PolyPhen (B), M-CAP (T), REVEL (0.1)
	0549	7y	female	N/A	N	JME	*KIAA1324L*	Y	c.1372G>A p.Gly458Arg	*de novo*	mosaic	N/A	CADD (24), SIFT (T), PolyPhen (P), M-CAP (D), REVEL (0.3)
	0010	5y	male	Mild	N		*SLITRK2*	Y	c.1276C>T p.Arg426Cys	maternal	hemi	N/A	CADD (26), SIFT (D), PolyPhen (D), M-CAP (D), REVEL (0.4)
	0902	3y	female	Borderline	N	CAE	*THAP11*	Y	c.790C>T p.Gln264Ter	*de novo*	hetero	N/A	LoF
	0919	10y	female	N/A	N	JME	*TNIP1*	Y	c.698G>T p.Arg233Leu	*de novo*	hetero	N/A	CADD (27), SIFT (D), PolyPhen (D), M-CAP (D), REVEL (0.2)
NAFE	0532	15y	male	Mild	N		*ACOT1*	Y	c.570del p.Tyr191IlefsTer22	unknown	homo	N/A	LoF
	1105	2.5y	male	Borderline	N		*ATP6V1B2*	Y	c.361A>T p.Thr121Ser	*de novo*	hetero	N/A	CADD (23), SIFT (T), PolyPhen (B), M-CAP (D), REVEL (0.5)
	0712	7y	male	N/A	N		*BNC2*	Y	c.1616T>G p.Met539Arg	*de novo*	hetero	N/A	CADD (22), SIFT (T), PolyPhen (B), M-CAP (T), REVEL (0.2)
	0502	6y	male	N/A	Y	SeLECTS	*C12orf45*	Y	c.527dupT p.Leu176PhefsTer7	*de novo*	hetero	N/A	LoF

© 2023 Koh HY et al. *JAMA Network Open.*

	1102	14y	male	N/A	N		*CACNA1F*	Y	c.4715C>A p.Pro1572Gln	maternal	hemi	N/A	CADD (26), SIFT (D), PolyPhen (D), M-CAP (D), REVEL (0.8)
	0928	5y	male	N/A	N	SHE	*CAMKK1*	Y	c.515G>A p.Arg172His	*de novo*	hetero	N/A	CADD (33), SIFT (D), PolyPhen (D), M-CAP (D), REVEL (0.5)
	0852	2y	female	N/A	N		*CHRM2*	Y	c.101G>A p.Ser34Asn	*de novo*	hetero	N/A	CADD (26), SIFT (D), PolyPhen (D), M-CAP (T), REVEL (0.3)
	0879	1w	female	N/A	N		*CPSF1*	Y	c.4102A>C p.Thr1368Pro	*de novo*	hetero	N/A	CADD (24), SIFT (D), PolyPhen (B), M-CAP (D), REVEL (0.2)
	0703	4y	female	N/A	N	SeLFE	*CSMD1*	Y	c.8009G>T p.Gly2670Val	*de novo*	hetero	N/A	CADD (24), SIFT (D), PolyPhen (D), M-CAP (T), REVEL (0.5)
	0978	10y	male	N/A	N	SeLECTS	*CUL3*	Y	c.535C>T p.Arg179Trp	*de novo*	hetero	N/A	CADD (27), SIFT (D), PolyPhen (D), M-CAP (D), REVEL (0.9)
	0929	7y	male	N/A	N	SeLECTS	*DPYSL2*	Y	c.1144G>A p.Glu382Lys	*de novo*	hetero	N/A	CADD (32), SIFT (D), PolyPhen (D), M-CAP (D), REVEL (0.9)
	0418	3y	male	Borderline	N		*GNL3L*	Y	c.965G>A p.Cys322Tyr	maternal	hemi	N/A	CADD (25), SIFT (D), PolyPhen (D), M-CAP (D), REVEL (0.6)
	0868	5y	female	Mild	N		*ITM2C*	Y	c.802T>C p.Ter268ArgextTer37	*de novo*	hetero	N/A	-
	0525	5y	male	N/A	N	SHE	*KLHL13*	Y	c.1321G>A p.Ala441Thr	maternal	hemi	N/A	CADD (26), SIFT (D), PolyPhen (D), M-CAP (D), REVEL (0.6)
	0928	5y	male	N/A	N	SHE	*MAP3K15*	Y	c.1843A>T p.Arg615Ter	maternal	hemi	N/A	LoF
	0607	7y	female	N/A	N	RS	*MEST*	Y	c.437A>C p.His146Pro	*de novo*	hetero	N/A	CADD (27), SIFT (D), PolyPhen (D), M-CAP (D), REVEL (0.9)
	0495	1y1m	male	Borderline	N		*MXRA5*	Y	c.6578G>A p.Ser2193Asn	maternal	hemi	N/A	CADD (28), SIFT (D), PolyPhen (D), M-CAP (D), REVEL (0.4)
	0423	1y	male	Borderline	N	SHE	*NOTCH2*	Y	c.2502G>C p.Leu834Phe	*de novo*	hetero	N/A	CADD (24), SIFT (D), PolyPhen (D), M-CAP (D), REVEL (0.5)
	0676	1y	male	N/A	N		*OTUD6A*	Y	c.52G>C p.Glu18Gln	maternal	hemi	N/A	CADD (23), SIFT (D), PolyPhen (D), M-CAP (D), REVEL (0.3)
	0570	2y	male	Moderate	N		*PPFIA3*	Y	c.115C>T p.Arg39Cys	*de novo*	hetero	N/A	CADD (32), SIFT (D), PolyPhen (D), M-CAP (D), REVEL (0.3)
	0617	2y	male	N/A	N		*PRPF31*	Y	c.1106C>T p.Thr369Met	*de novo*	mosaic	N/A	CADD (28), SIFT (D), PolyPhen (D), M-CAP (D), REVEL (0.8)
	0918	5y	male	N/A	N		*RPS4X*	Y	c.703T>C p.Trp235Arg	maternal	hemi	N/A	CADD (24), SIFT (T), PolyPhen (B), M-CAP (D), REVEL (0.4)
	0780	14y	male	N/A	N		*SCUBE1*	Y	c.1064A>G p.Asp355Gly	*de novo*	hetero	N/A	CADD (27), SIFT (D), PolyPhen (P), M-CAP (D), REVEL (1)
	0539	9y	male	N/A	N		*SIPA1L1*	Y	c.4754C>T p.Ser1585Leu	*de novo*	hetero	N/A	CADD (28), SIFT (D), PolyPhen (D), M-CAP (D), REVEL (0.5)
	0540	8y	female	N/A	N		*STK3*	Y	c.976A>G p.Lys326Glu	*de novo*	hetero	N/A	CADD (23), SIFT (T), PolyPhen (B), M-CAP (D), REVEL (0.4)
	0502	6y	male	N/A	Y	SeLECTS	*SYTL5*	Y	c.278G>C p.Arg93Pro	maternal	hemi	N/A	CADD (24), SIFT (D), PolyPhen (D), M-CAP (D), REVEL (0.6)

© 2023 Koh HY et al. *JAMA Network Open.*

	0745	7m	female	N/A	N		*TMEM151A*	Y	c.1173dup p.Ala392ArgfsTer53	both	homo	0.000	LoF
	1105	2.5y	male	Borderline	N		*TNRC6A*	Y	c.5015C>T p.Ala1672Val	*de novo*	hetero	N/A	CADD (28), SIFT (D), PolyPhen (D), M-CAP (D), REVEL (0.3)
	0933	8y	female	N/A	N		*YBX3*	Y	c.311A>G p.Tyr104Cys	*de novo*	hetero	N/A	CADD (31), SIFT (D), PolyPhen (D), M-CAP (T), REVEL (0.5)
	0929	7y	male	N/A	N	SeLECTS	*ZBTB38*	Y	c.1121G>A p.Cys374Tyr	*de novo*	hetero	N/A	CADD (28), SIFT (D), PolyPhen (D), M-CAP (D), REVEL (0.9)
Combined	0845	12y	female	N/A	N		*ALKBH1*	Y	c.869C>T p.Pro290Leu	*de novo*	hetero	N/A	CADD (30), SIFT (D), PolyPhen (D), M-CAP (D), REVEL (0.5)
	0871	2y	male	N/A	N	SWAS	*ANLN*	Y	c.1285C>T p.Gln429Ter	*de novo*	hetero	N/A	LoF
	0831	6y	male	N/A	N		*DNAJC4*	Y	c.691del p.Gln231ArgfsTer73	*de novo*	hetero	N/A	LoF
	0779	1y1m	male	N/A	N	GEFS**+**	*FAF1*	Y	c.3G>A p.Met1?	*de novo*	hetero	N/A	LoF
	0587	8y	female	N/A	N	LGS	*FIZ1*	Y	c.1403G>T p.Arg468Leu	*de novo*	hetero	N/A	CADD (25), SIFT (D), PolyPhen (D), M-CAP (D), REVEL (0.2)
							FIZ1	Y	c.997T>G p.Cys333Gly	*de novo*	hetero	N/A	CADD (24), SIFT (D), PolyPhen (D), M-CAP (D), REVEL (0.4)
	0931	1y6m	female	Borderline	N		*HELB*	Y	c.662T>C p.Leu221Pro	*de novo*	hetero	N/A	CADD (27), SIFT (D), PolyPhen (D), M-CAP (D), REVEL (0.4)
	0753	2y	female	N/A	N	LKS	*MCTP1*	Y	c.1688A>C p.Gln563Pro	*de novo*	hetero	N/A	CADD (25), SIFT (D), PolyPhen (B), M-CAP (D), REVEL (0.1)
	0789	1y	female	Borderline	N		*NEURL4*	Y	c.2262G>C p.Arg754=	*de novo*	hetero	N/A	Acceptor loss at 0
	0644	11m	male	N/A	N		*PDE4C*	Y	c.1329_1330+2delCAGT	*de novo*	hetero	N/A	LoF
	1044	11y	male	Moderate	Y		*PLXNB3*	Y	c.5008C>A p.Leu1670Met	maternal	hemi	N/A	CADD (22), SIFT (D), PolyPhen (D), M-CAP (D), REVEL (0.2)
	0519	5y	female	N/A	N		*RYK*	Y	c.6_7insA p.Gly3ArgfsTer78	*de novo*	hetero	N/A	LoF
	0499	2y6m	female	Borderline	Y		*SLC2A8*	Y	c.802C>T p.Gln268Ter	both	homo	0.001	LoF
	0813	2y	female	N/A	N		*SNRNP200*	Y	c.3388C>T p.Arg1130Cys	*de novo*	hetero	0.000	CADD (32), SIFT (D), PolyPhen (D), M-CAP (D), REVEL (0.7)
	0811	2y	male	Moderate	N		*TUBA8*	Y	c.4C>T p.Arg2Trp	both	homo	N/A	CADD (26), SIFT (D), PolyPhen (D), M-CAP (D), REVEL (0.7)
	0983	6y	female	Borderline	N		*XKR6*	Y	c.1577_1587del p.Val526GlyfsTer3	*de novo*	hetero	N/A	LoF

ID, intellectual disability; ASD, autism spectrum disorder; AF, allele frequency; DEE, developmental and epileptic encephalopathies; GGE, genetic generalized epilepsy; IGE, idiopathic generalized epilepsy; NAFE, non-acquired focal epilepsy; N/A, not applicable; Y, yes; N, none; IESS, infantile epileptic spasm syndrome; LGS, Lennox-Gastaut syndrome; EE-SWAS, epileptic encephalopathy with spike-and-wave activation in sleep; FIRES, febrile infection-related epilepsy syndrome; MEI, myoclonic epilepsy of infancy; EEM, epilepsy with eyelid myoclonia; JME, juvenile myoclonic epilepsy; CAE, childhood absence epilepsy; SeLECTS, self-limited epilepsy with centrotemporal spikes; SHE, sleep-related hypermotor (hyperkinetic) epilepsy; SeLFE, self-limited focal epilepsy; RS, Rasmussen syndrome; SWAS, spike- and-wave activation in sleep; GEFS+, generalized epilepsy with febrile seizure plus; LKS, Landau–Kleffner syndrome; CADD, combined annotation dependent depletion; SIFT, sorting intolerant from tolerant; M-CAP, Mendelian clinically applicable pathogenicity; REVEL, rare exome variant ensemble learner; D, damaging; T; tolerable; B, benign; P, possibly damaging; LoF, loss of function.

© 2023 Koh HY et al. *JAMA Network Open.*

eTable 5. Clinical and Diagnostic Genetic Findings in 11 Cases With CNVs

Coordination (hg19)	Outcomes and involved genes	Zygosity	AF	Cases	Seizure onset	Sex	Seizure type(s)	ID	ASD	ASM response
chr2:166847505-167334456	*SCN1A*	hetero	N/A	0625	1y	male	Generalized tonic, GTCS	Borderline	N	Refractory
chr3:11058648-11060634	*SLC6A1*	hetero	N/A	0507	1y10m	female	Generalized absence, typical	N/A	N	Refractory
chr16:138446-140150	*NPRL3*	hetero	N/A	0696	4m	male	Infantile spasm (4m), focal	Borderline	N	Refractory
chr22:32121274-32302733	*DEPDC5*	hetero	N/A	0739	6y	male	Focal, tonic	Borderline	Y	Refractory
chr22:32193336-32194893	*DEPDC5*	hetero	N/A	0760	3y	male	Focal motor to bilateral tonic-clonic	N/A	N	Controlled
chr1:146630894-147415874	1q21.1 recurrent microdeletion	hetero	5x in gnomAD	0816	5y	female	Generalized absence, typical; GTCS	N/A	N	Controlled
chr15:30896079-32404350	15q13.3 recurrent microdeletion	hetero	1x in gnomAD	0547	6y	female	GTCS, generalized absence, typical with eyelid myoclonia	Mild	N	Controlled
chr16:29674800-30199626	16p11.2 recurrent microduplication	hetero	1x in gnomAD	0848	8m	male	Focal motor to bilateral tonic-clonic	N/A	Y	Controlled
chr16:21964495-22385880	16p12.1 recurrent microdeletion	hetero	5x in gnomAD	0419	4y	male	GTC, absence	Mild	Y	Controlled
chr16:14960162-16297720	16p13.11 recurrent microdeletion	hetero	3x in gnomAD	0936	14y	male	Focal to bilateral tonic-clonic	Moderate	N	Refractory
chr22:18893638-21386351	22q11 deletion (DiGeorge) syndrome	hetero	N/A	0947	6y	female	GTCS	Borderline	N	Controlled

CNV, copy number variant; AF, allele frequency; N/A, not available; ID, intellectual disability; ASD, autism spectrum disorder; ASM, anti-seizure medication; N/A, not applicable; GTCS, generalized tonic-clonic seizure; GTC, generalized tonic seizure; Y, yes; N, none.

© 2023 Koh HY et al. *JAMA Network Open.*

eTable 6. Bivariate and Multivariate Regression Model of Phenotypic Variables to Diagnostic Yield by ES

	model 1 (bivariate)			model 2 (multivariate)		
	Coefficient	*P* value	OR (95% CI)	Coefficient	*P* value	adjusted OR (95% CI)
Sex	0.02	0.92	1.02 (0.66 – 1.58)	-0.16	0.49	0.85 (0.53 – 1.36)
Age of seizure onset	-0.13	**0.15×10^{-3}**	**0.88 (0.82 – 0.94)**	-0.08	**0.03**	**0.93 (0.87 – 0.99)**
DEE or ID	1.31	**0.27×10^{-6}**	**3.69 (2.24 – 6.06)**	0.89	**0.00**	**2.44 (1.40 – 4.26)**
ASD	0.53	0.06	1.70 (0.98 – 2.97)	0.10	0.76	1.10 (0.60 – 2.02)
ADHD	0.04	0.89	1.04 (0.57 – 1.93)	0.02	0.95	1.02 (0.54 – 1.95)
Motor impairment	1.19	**0.20×10^{-6}**	**3.29 (2.10 – 5.15)**	0.79	**0.00**	**2.19 (1.34 - 3.58)**
History of afebrile seizure in a parent	-0.27	0.58	0.76 (0.29 – 2.03)	-0.22	0.68	0.80 (0.29 - 2.26)

Bold numbers indicate $p < 0.05$. ID, intellectual disability; DEE, developmental and epileptic encephalopathies; ASD, autism spectrum disorder; ADHD, attention deficit hyperactivity disorder.

© 2023 Koh HY et al. *JAMA Network Open.*

References

1. Brownstein CA, Goldstein RD, Thompson CH, et al. SCN1A variants associated with sudden infant death syndrome. *Epilepsia*. Apr 2018;59(4):e56-e62. doi:10.1111/epi.14055
2. Rockowitz S, LeCompte N, Carmack M, et al. Children's rare disease cohorts: an integrative research and clinical genomics initiative. *NPJ Genom Med*. 2020;5:29. doi:10.1038/s41525-020-0137-0
3. Guethbjartsson H, Georgsson GF, Guethjonsson SA, et al. GORpipe: a query tool for working with sequence data based on a Genomic Ordered Relational (GOR) architecture. *Bioinformatics*. Oct 15 2016;32(20):3081-3088. doi:10.1093/bioinformatics/btw199
4. Jiang H, Lei R, Ding SW, Zhu S. Skewer: a fast and accurate adapter trimmer for next-generation sequencing paired-end reads. *BMC Bioinformatics*. Jun 12 2014;15:182. doi:10.1186/1471-2105-15-182
5. Andrews S. FastQC: a quality control tool for high throughput sequence data. Babraham Bioinformatics, Babraham Institute, Cambridge, United Kingdom; 2010.
6. Bushnell B. *BBMap: a fast, accurate, splice-aware aligner*. 2014.
7. Kendig KI, Baheti S, Bockol MA, et al. Sentieon DNASeq Variant Calling Workflow Demonstrates Strong Computational Performance and Accuracy. *Front Genet*. 2019;10:736. doi:10.3389/fgene.2019.00736
8. Jun G, Flickinger M, Hetrick KN, et al. Detecting and estimating contamination of human DNA samples in sequencing and array-based genotype data. *Am J Hum Genet*. Nov 2 2012;91(5):839-48. doi:10.1016/j.ajhg.2012.09.004
9. McLaren W, Gil L, Hunt SE, et al. The Ensembl Variant Effect Predictor. *Genome Biol*. Jun 6 2016;17(1):122. doi:10.1186/s13059-016-0974-4
10. Landrum MJ, Lee JM, Benson M, et al. ClinVar: public archive of interpretations of clinically relevant variants. *Nucleic Acids Res*. Jan 4 2016;44(D1):D862-8. doi:10.1093/nar/gkv1222
11. Lek M, Karczewski KJ, Minikel EV, et al. Analysis of protein-coding genetic variation in 60,706 humans. *Nature*. Aug 18 2016;536(7616):285-91. doi:10.1038/nature19057
12. Rentzsch P, Witten D, Cooper GM, Shendure J, Kircher M. CADD: predicting the deleteriousness of variants throughout the human genome. *Nucleic Acids Res*. Jan 8 2019;47(D1):D886-D894. doi:10.1093/nar/gky1016
13. Jaganathan K, Kyriazopoulou Panagiotopoulou S, McRae JF, et al. Predicting Splicing from Primary Sequence with Deep Learning. *Cell*. Jan 24 2019;176(3):535-548 e24. doi:10.1016/j.cell.2018.12.015
14. Robinson JT, Thorvaldsdottir H, Winckler W, et al. Integrative genomics viewer. *Nat Biotechnol*. Jan 2011;29(1):24-6. doi:10.1038/nbt.1754
15. Babadi M, Lee SK, Smirnov A, et al. Precise common and rare germline CNV calling with GATK. *Cancer Research*. 2018;78(13_Supplement):2287-2287.
16. Firth HV, Richards SM, Bevan AP, et al. DECIPHER: Database of Chromosomal Imbalance and Phenotype in Humans Using Ensembl Resources. *Am J Hum Genet*. Apr 2009;84(4):524-33. doi:10.1016/j.ajhg.2009.03.010

© 2023 Koh HY et al. *JAMA Network Open.*

eFigures

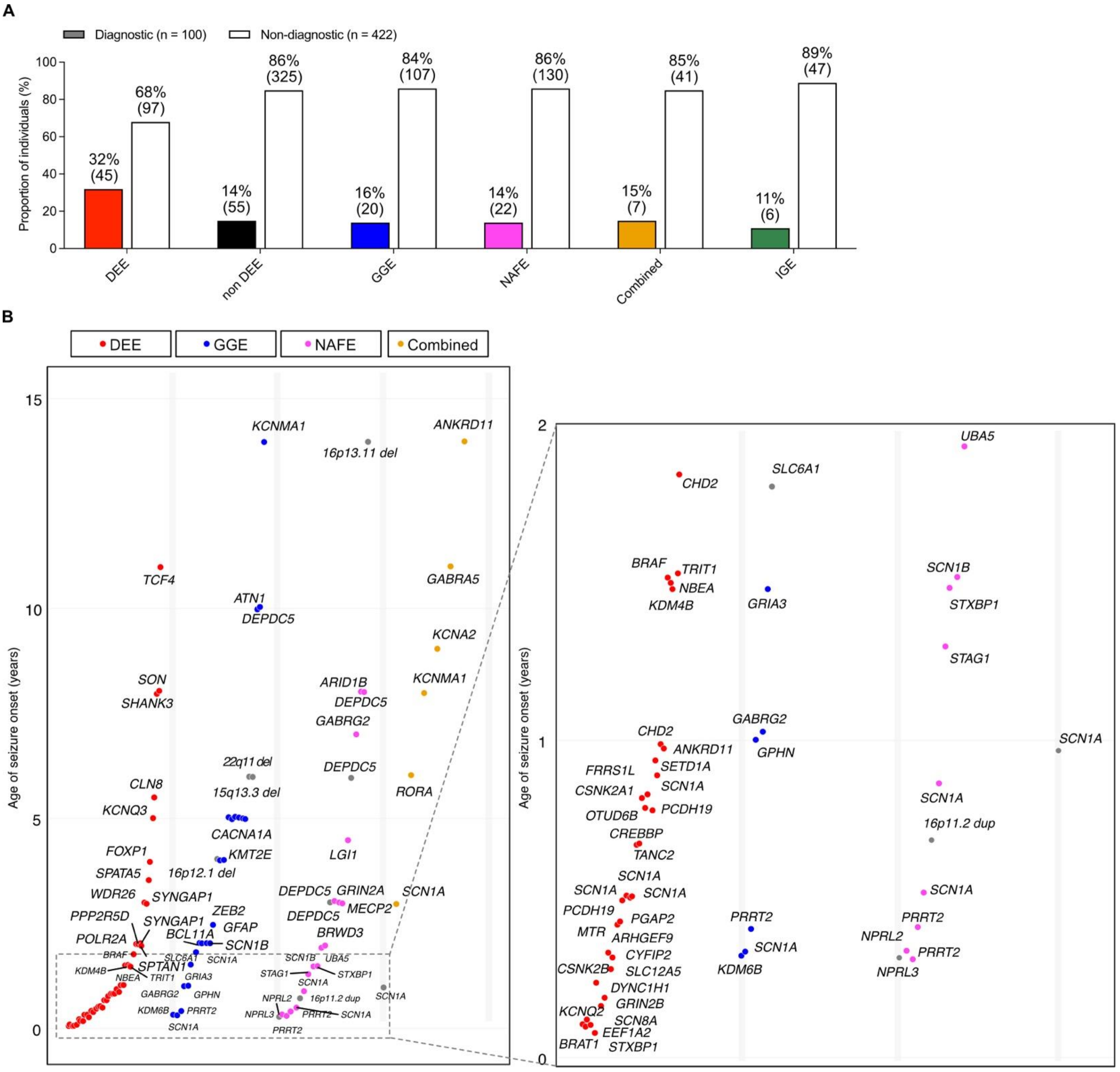

eFigure 1. Diagnostic Yield and Age of Seizure Onset According to Epilepsy Type

(**A**) We demonstrate the proportion of individuals with each epilepsy type with respect to whether they had diagnostic vs. non-diagnostic findings. DEE was significantly associated with the presence of diagnostic variants. The percentage of diagnostic yield and number of individuals in each group is noted at the top of the columns. (**B**) We display the genes responsible for epilepsy in DEE, GGE, NAFE, and combined generalized and focal epilepsy in the 100 patients with diagnostic findings (89 SNVs, 11 CNVs). Findings of IGE are involved in GGE category. Each circle represents a diagnostic variant, either P/LP or diagnostic VUS, presented according to the age of seizure onset (Y axis) and epilepsy types (top labels). The box on the right details the genes identified in individuals under 2 years of age. Diagnostic CNVs are indicated by gray circles. (DEE: developmental and epileptic encephalopathy, GGE: genetic generalized epilepsy, NAFE: non-acquired focal epilepsy)

© 2023 Koh HY et al. *JAMA Network Open.*

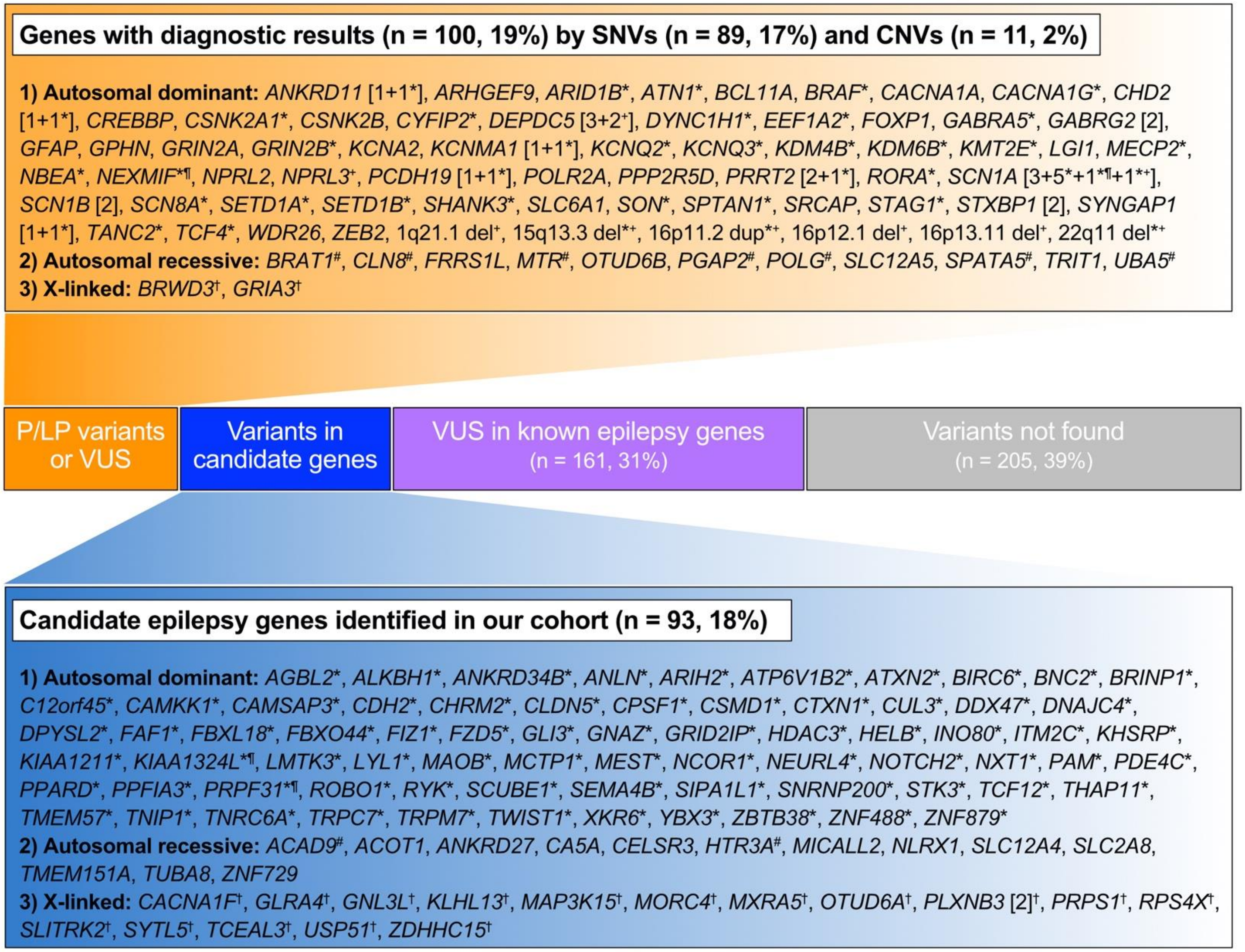

eFigure 2. Established and Candidate Epilepsy Genes in the Study Cohort

Analyzing exome sequencing data from 522 patients with unexplained epilepsy, we identified diagnostic variants in 89 cases, 81 with P/LP variants and 15 individuals with VUS, 8 of which we determined to be diagnostic. These variants involve 69 genes (orange, details in Table 2 and eTable 1) associated with autosomal dominant, autosomal recessive, and X-linked conditions. Additionally, diagnostic CNVs were identified in 11 patients (details in Table 2 and eTable 5). In total, we identified diagnostic results for 100 patients. VUS in known epilepsy genes (in eTable2) were present in 161 individuals (purple, detailed information on variants provided in eTable 3). We identified rare, predicted damaging variants in candidate genes for epilepsy (genes of uncertain significance) in 93 cases, most of them apparently *de novo* variants (blue, detailed information on variants provided in eTable 4), and no explanatory or candidate variants for 205 cases (gray). The boxes list the identified genes (with the number of cases per gene shown in brackets if greater than 1) and, if known, their associated modes of inheritance (**de novo*; #compound heterozygous; †X-linked hemizygous; ¶mosaic; +CNV). (SNV: single nucleotide variant, CNV: copy number variant, P: pathogenic, LP: likely pathogenic, VUS: variant(s) of uncertain significance)

© 2023 Koh HY et al. *JAMA Network Open.*

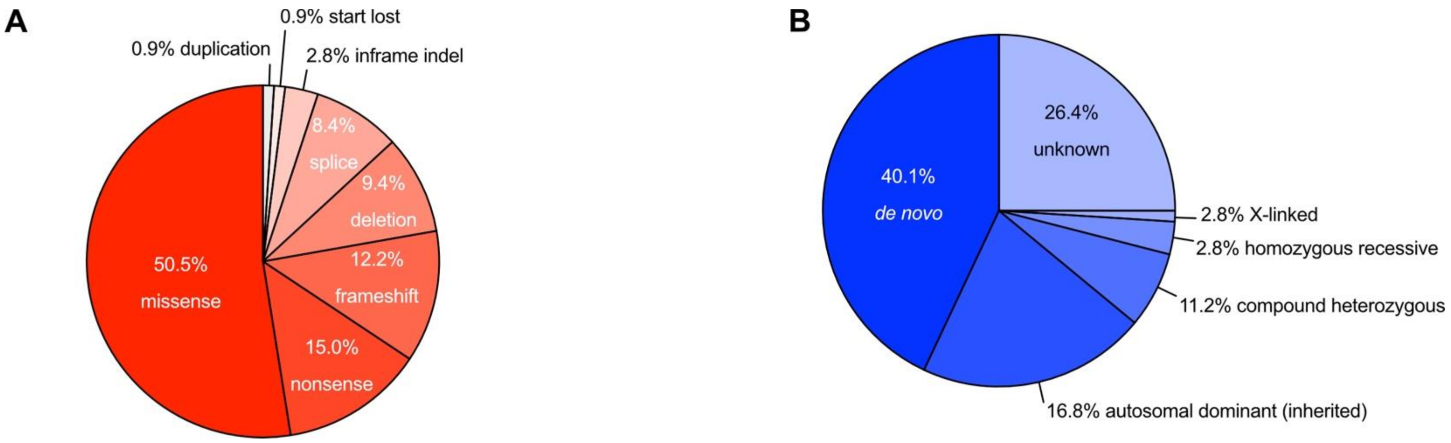

eFigure 3. Variant Types Among the Diagnostic SNVs and CNVs

We depict the predicted variant effects (**A**) and variant inheritance or *de novo* status (**B**). (SNV: single nucleotide variant, CNV: copy number variant, P: pathogenic, LP: likely pathogenic, VUS: variant(s) of uncertain significance)

© 2023 Koh HY et al. *JAMA Network Open.*

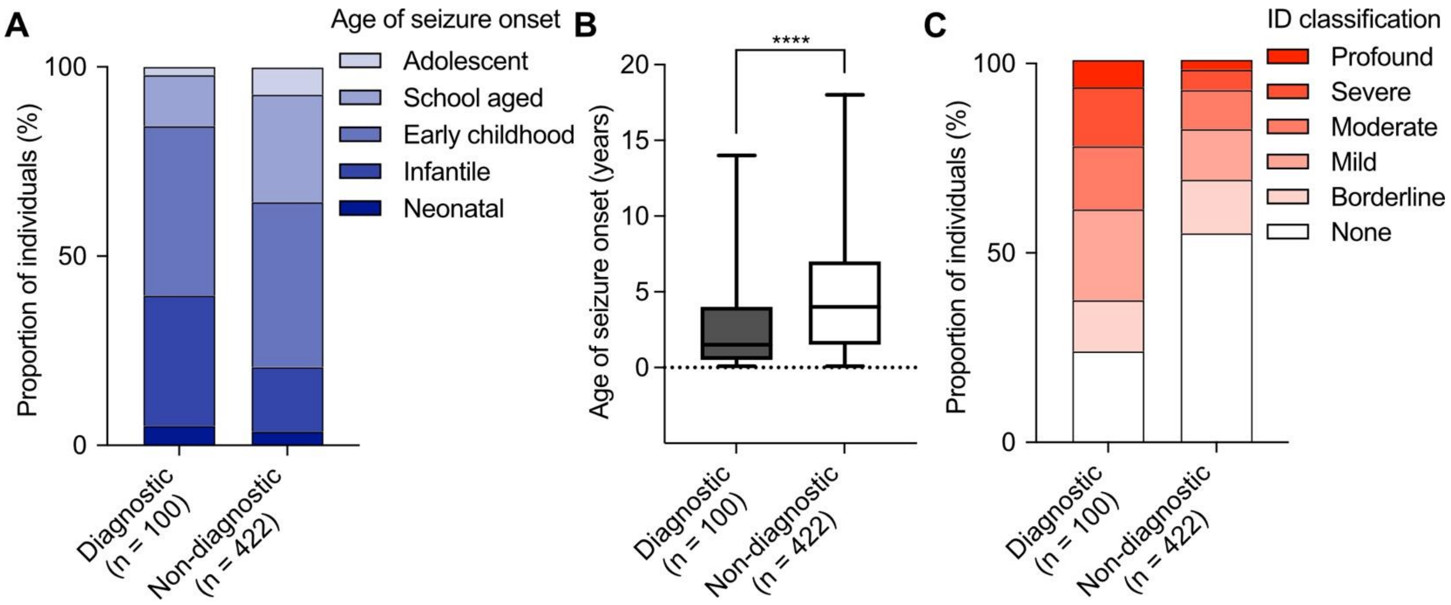

eFigure 4. Clinical Features in Patients With Epilepsy Associated With Diagnostic Variants
(**A-B**) We evaluated the age ranges (represented in the **A**) and age of seizure onset (**B**) in diagnostic vs. non-diagnostic children with epilepsy. (**C**) Proportions of individuals with ID in the diagnostic vs. non-diagnostic groups are shown. ****$P < 0.0001$. (ID: intellectual disability)

© 2023 Koh HY et al. *JAMA Network Open.*

Appendix

Working group authors in BCH Neurology Referral and Phenotyping Group:

Elizabeth Barkoudah, MD
Ann M. Bergin, MD
Miya Bernson-Leung, MD, EdM
Elizabeth Binney, MD
Jeffrey Bolton, MD
Stephanie Donatelli, MD
Darius Ebrahimi-Fakhari, MD, PhD
Mark P. Gorman, MD
Chellamani Harini, MD
Divya Jayaraman, MD, PhD
Agnieszka A. Kielian, MD
Lauren LaFortune, MD
Kerri Larovere, MD
Mark Libenson, MD
David N. Lieberman, MD, PhD
Tobias Loddenkemper, MD
Candice E. Marti, CPNP
Anna Minster, MD
Kate Mysak, MS, CPNP
Ann Paris, MS, CPNP, CNRN
Archana A. Patel, MD, MPH
Phillip L. Pearl, MD
Jurriaan M. Peters, MD, PhD
Anna Pinto, MD, PhD
Peter Raffalli, MD
Alexander Rotenberg, MD, PhD
Catherine Salussolia, MD, PhD
Rebecca Sarvendram, CPNP
Hannah Shapiro, MD
Janet Soul, MD
Sarah Spence, MD, PhD
Karen Spencer, MD, MS, MPH
Robert C. Stowe, MD
Coral M. Stredny, MD
Masanori Takeoka, MD
Molly Tracy, MD
Sara K. Trowbridge, MD
Melissa Tsuboyama, MD
David K. Urion, MD

© 2023 Koh HY et al. *JAMA Network Open.*
