# Supplementary material for: Utility of Exome Sequencing for Diagnosis in Unexplained Pediatric-Onset Epilepsy
Source: JAMA Netw Open. 2023 Jul 20;6(7):e2324380. doi: 10.1001/jamanetworkopen.2023.24380 (PMC10359957; doi:10.1001/jamanetworkopen.2023.24380)
Supplement: Supplement 2. — Members of the BCH Neurology Referral and Phenotyping Group [file jamanetwopen-e2324380-s002.pdf]

Supplemental Online Content: Nonauthor Collaborators
*First name, last name, and suffix (if applicable) are required and will appear in PubMed.

*Group Name(s): BCH Neurology Referral and Phenotyping Group							
***First Name and Middle Initial(s)**	***Last Name**	***Suffix (eg, Jr, III)**	Academic Degrees	Institution	Location (city, state/province, country)	Role or Contribution, eg, chair, principal investigator	Group (if more than 1 Group listed in the byline) and/or Subgroup (eg, Steering Committee)
Elizabeth	Barkoudah						
Ann M.	Bergin						
Miya	Bernson-Leung						
Elizabeth	Binney						
Jeffrey	Bolton						
Stephanie	Donatelli						
Darius	Ebrahimi-Fakhari						
Mark P.	Gorman						
Chellamani	Harini						
Divya	Jayaraman						
Agnieszka A.	Kielian						
Lauren	LaFortune						
Kerri	Larovere						
Mark	Libenson						
David N.	Lieberman						
Tobias	Loddenkemper						
Candice E.	Marti						
Anna	Minster						
Kate	Mysak						
Ann	Paris						
Archana A.	Patel						
Phillip L.	Pearl						
Jurriaan M.	Peters						
Anna	Pinto						
Peter	Raffalli						
Alexander	Rotenberg						
Catherine	Salussolia						
Rebecca	Sarvendram						
Hannah	Shapiro						
Janet	Soul						
Sarah	Spence						

Supplemental Online Content: Nonauthor Collaborators
*First name, last name, and suffix (if applicable) are required and will appear in PubMed.

***First Name and Middle Initial(s)**	***Last Name**	***Suffix (eg, Jr, III)**	Academic Degrees	Institution	Location (city, state/province, country)	Role or Contribution, eg, chair, principal investigator	Group (if more than 1 Group listed in the byline) and/or Subgroup (eg, Steering Committee)
Karen	Spencer						
Robert C.	Stowe						
Coral M.	Stredny						
Masanori	Takeoka						
Molly	Tracy						
Sara K.	Trowbridge						
Melissa	Tsuboyama						
David K.	Urion						
