# Supplementary material for: Utility of Exome Sequencing for Diagnosis in Unexplained Pediatric-Onset Epilepsy
Source: JAMA Netw Open. 2023 Jul 20;6(7):e2324380. doi: 10.1001/jamanetworkopen.2023.24380 (PMC10359957; doi:10.1001/jamanetworkopen.2023.24380)
Supplement: Supplement 3. — Data Sharing Statement [file jamanetwopen-e2324380-s003.pdf]

Data Sharing Statement

Koh. Utility of Exome Sequencing for Diagnosis in Unexplained Pediatric-Onset Epilepsy. *JAMA Netw Open*. Published July 20, 2023. doi:10.1001/jamanetworkopen.2023.24380

Data
Data available: Yes
Data types: Deidentified participant data
How to access data: Data will be made available on request from annapurna.poduri@childrens.harvard.edu.
When available: With publication

Supporting Documents
Document types: None

Additional Information
Who can access the data: anyone requesting the data whose protocols allow use of deidentified phenotypic and genomic data
Types of analyses: for research purposes
Mechanisms of data availability: with a signed data use agreement
Any additional restrictions: n/a
